# New insights into functional regulation in MS-based drug profiling

**DOI:** 10.1038/srep18826

**Published:** 2016-01-08

**Authors:** Ana Sofia Carvalho, Henrik Molina, Rune Matthiesen

**Affiliations:** 1 Computational and Experimental Biology Group, National Health Institute Dr. Ricardo Jorge, IP, Av. Padre Cruz, 1649-016, Lisbon, Portugal; 2 Proteomics Resource Center, The Rockefeller University, 1230 York Avenue, New York, New York 10065-6399.

## Abstract

We present a novel data analysis strategy which combined with subcellular fractionation and liquid chromatography–mass spectrometry (LC-MS) based proteomics provides a simple and effective workflow for global drug profiling. Five subcellular fractions were obtained by differential centrifugation followed by high resolution LC-MS and complete functional regulation analysis. The methodology combines functional regulation and enrichment analysis into a single visual summary. The workflow enables improved insight into perturbations caused by drugs. We provide a statistical argument to demonstrate that even crude subcellular fractions leads to improved functional characterization. We demonstrate this data analysis strategy on data obtained in a MS-based global drug profiling study. However, this strategy can also be performed on other types of large scale biological data.

Currently employed approaches for global drug profiling include methods based on epigenomics by next generation sequencing^1^, transcriptomics using either microarrays or next generation sequencing^2^ and mass spectrometry for profiling proteins^3^ and metabolites^4^. Global proteome MS-based drug profiling was originally grounded on 2D gel electrophoresis for separation and quantitation followed by mass spectrometry based identification^5^. With the latest generation of sensitive and high resolution accurate mass spectrometers, new methods are emerging which can be divided into two main methodologies: (1) pre-fractionation of peptides and/or (2) pre-fractionation of proteins previous to LC-MS. Multi-dimensional liquid chromatography^6^,^7^ and isoelectric focusing^8^ are examples of peptide pre-fractionation methods. One-dimensional SDS-polyacrylamide gel electrophoresis^9^,^10^, size exclusion chromatography^11^ and to a less extent subcellular fractionation^5^,^10^ have been used to resolve protein mixtures prior to LC-MS analysis.

State-of-art LC-MS instruments produce large quantities of spectral data. Further, relative quantitative data can be obtained based on label free or stable isotope labelling methods. Interpretation of LC-MS spectra across samples in bottom-up proteomics leads to two types of quantitative matrices, irrespectively of the strategy or labelling methods used for data collection. One matrix contains quantitative information on the peptide level across samples and the other contains protein quantitation information. A key challenge is to extract biological relevant information from the two matrices. A common strategy can be outlined as following: (1) replace missing values (e.g. using the average or the median values within a sample group), (2) log transform the quantitative data, (3) normalize the data across samples, 4) apply statistical analysis (such as ANOVA to compare multiple sample groups followed by a post hoc test, Significance Analysis of Microarrays (SAM) and t test to compare two sample groups, and (5) define groups of significant regulated proteins which are subjected to functional enrichment analysis. In general significant regulated proteins are defined by applying filters to log ratios and P values followed by functional enrichment analysis using tools such as bioinformatics server DAVID^12^ (i.e. Individual Entity Analysis, see Fig. 1A). However, such methods are sensitive to the applied P value and log ratio thresholds. Consequently, several alternative approaches have been proposed in which the statistical analysis is performed on quantitative data for each functional group (Entity Set Analysis, see Fig. 1B). Different statistical methods for functional analysis of large scale biological data based on the statistical strategies, outlined in Fig. 1A,B, have been reviewed by Nam *et al.*^13^. Traditionally these statistical methods were developed for technologies that collect gene data such as e.g. microarray platforms. Nevertheless, this methodology can be explored successfully for proteins and in theory for metabolites as well. We consequently have renamed these methodologies by replacing “gene” with “entity” (Fig. 1).

We applied a novel concept, designated “Complete Functional Regulation Analysis”, which results from combining “Entity Set Analysis” and “Complete Entity Analysis” (Fig. 1). “Complete Entity Analysis” has to the best of our knowledge not been previously described. “Complete Entity Analysis” is useful for the characterization of the overall identified or detected entities in a given sample using a specific method. We provide a detailed theoretical basis for calculating the P value for “Complete Entity Analysis” in the methods section “Complete Functional Regulation Analysis”. The concept of “Complete Entity Analysis” can in principle be accomplished by “Individual Entity Analysis” using standard software, such as the online tool DAVID^14^, by excluding any pre-filtering steps. The upper limit of entities that can be submitted to DAVID is 3000, though. DAVID is therefore not compatible with the analysis performed here on all identified proteins in different subcellular fractions. The P values calculated by “Complete Entity Analysis” are used as a measure of how well specific functional categories are detected in a given sample. We here demonstrate that a standard subcellular fractionation method^15^, combined with LC-MS followed by a novel “Complete Functional Regulation Analysis” provides an effective and powerful technology for gaining functional insight into drug effects. “Complete Functional Regulation Analysis” condenses the statistical significant results into a single heatmap for each type of functional annotation (e.g. cellular component, biological process, molecular function, KEGG, etc).

## Results

**Figure 2:**
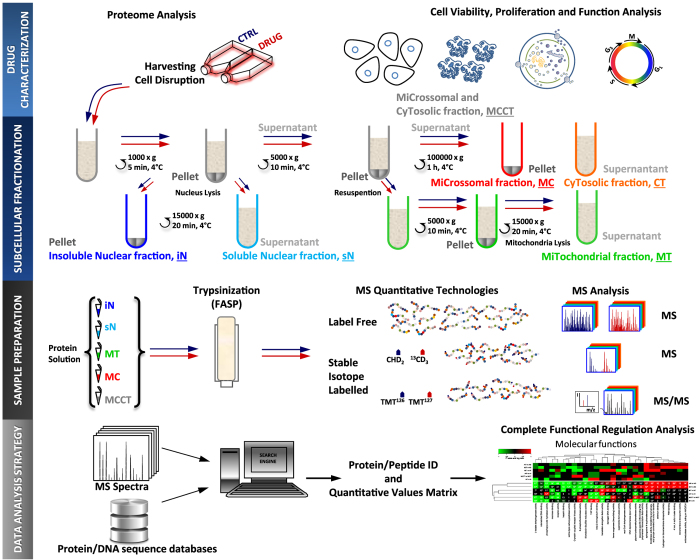
Overview of global MS-based drug profiling using five subcellular fractions and complete regulative functional analysis. The strategy constitutes a novel MS-based drug profiling and data analysis workflow.

For proof of concept of “Complete Functional Regulation Analysis” we have prepared five subcellular fractions using previously described standard methodologies^15^ (Fig. 2) for both untreated and glucosamine treated cancer cells.

**Figure 3:**
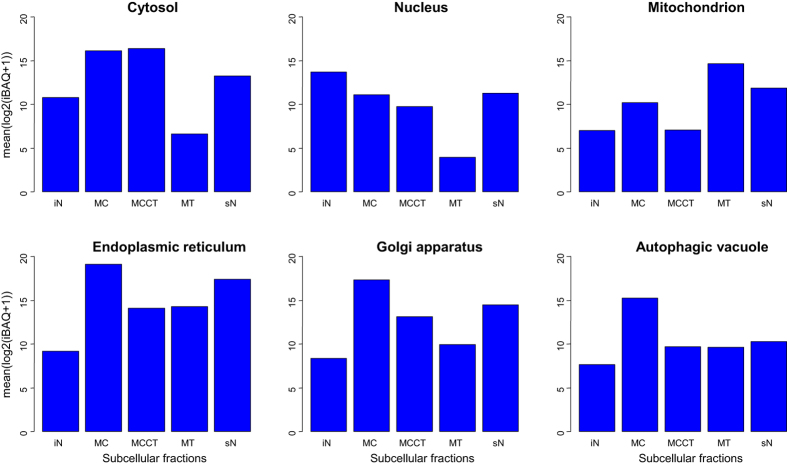
Mean log2 iBAQ values for each of the five subcellular fractions.

Previously, we have characterized the response of a cancer cell line to glucosamine treatment. Therein we analyzed distinctively the MS data from the MiCrossomal and CyTosolic (MCCT), Soluble Nuclear (sN) and MiTochondrial (MT) crude fractions (Fig. 2). Additionally, MS data for the Insoluble Nuclear (iN) and MiCrossomal (MC) crude fractions were obtained for the analysis presented herein, using fractions obtained simultaneously with our previous study. Thus, the total data set consists of 30 LC-MS runs: 3 (replicas) x 5 (subcellular fractions) x 2 (control and treated). The subcellular fractions are designated MCCT/MCCTT, sN/sNT, MT/MTT, MC/MCT and iN/iNT, where the absence or presence of ending “T” indicate control or treated, respectively. The fractions MCCT, MT and sN were previously validated by Western blot which confirmed that the expected proteins were indeed enriched^5^. The fractions MCCT, MT and sN were also found, based on the identified proteins, to mainly contain cytosolic, mitochondrial and nuclear proteins, respectively. For quantification intensity Based Absolute Quantitation^16^ (iBAQ) were estimated. To further characterize the five subcellular fractions we additionally calculated the mean log2(iBAQ+1) values for each of the five subcellular fractions over six major subcellular compartments (Fig. 3).

For cytosol annotated proteins the log2 difference in mean log2 iBAQ values between MCCT/MC and sN is approximately two which correspond to a fourfold difference on average. For nucleus annotated proteins, the mean log2 iBAQ abundance values are higher for iN and sN as expected. We also observed that the sN fraction has a relative high content of proteins annotated to endoplasmic reticulum and Golgi apparatus but not to autophagic vacuoles. The iN fraction on the other hand showed higher mean log2 iBAQ abundance values for nucleus annotated proteins compared to the sN fraction. Furthermore, the iN fraction displayed less cross contamination from endoplasmic reticulum and Golgi apparatus compared to the sN fraction. The MC fraction was found to have high mean log2 iBAQ abundance values for the cytosol, endoplasmic reticulum, Golgi apparatus and autophagic vacuole.

Following we have applied the above discussed “Complete Functional Regulation Analysis” on the quantitative data obtained by LC-MS label free quantitation to the samples described in Fig. 2. As mentioned above, applying filters to log ratios and P values followed by Individual Entity Analysis (IEA) leads to results which are sensitive to the applied P value and log ratio thresholds.

**Figure 4:**
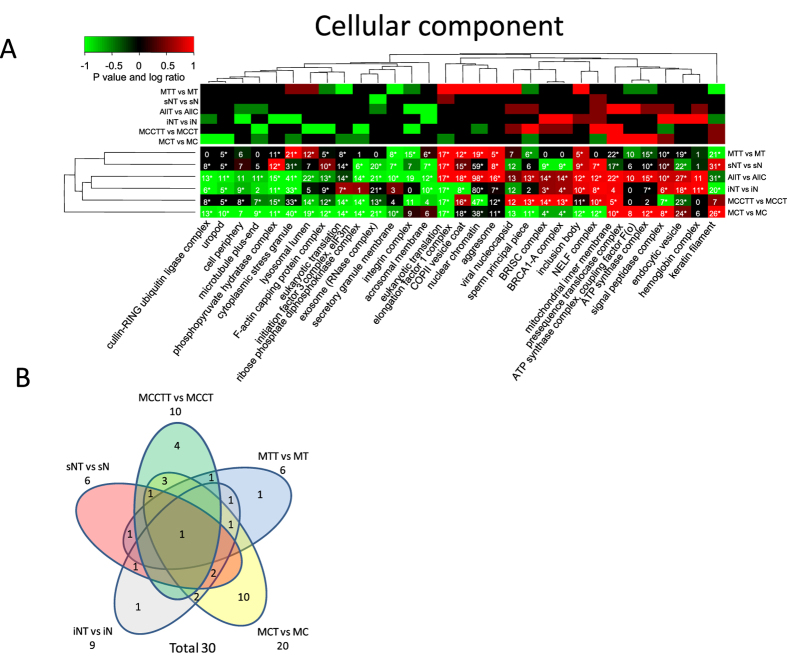
Functional regulation analysis filtered for minimum 10 proteins per category. (**A**) The lower heatmap panel indicates significant up- or down- regulated cellular component from gene ontology categories. P values are log ratio encoded (see methods sections for details). The upper panel indicates the log ratios where color code for log ratio 1 indicates a log ratio > = 1 and for −1 indicates log ratio < = −1. The numbers in each cell indicate the maximum number of proteins identified across all replicas for a specific protein category and subcellular fraction.*) indicates that the functional enrichment analysis is significant (P value <0.05 after FDR correction for multiple testing). (**B**) Venn diagram of the overlap of significant regulated cellular component categories. Overlaps with no numbers indicate zero overlap.

**Figure 5:**
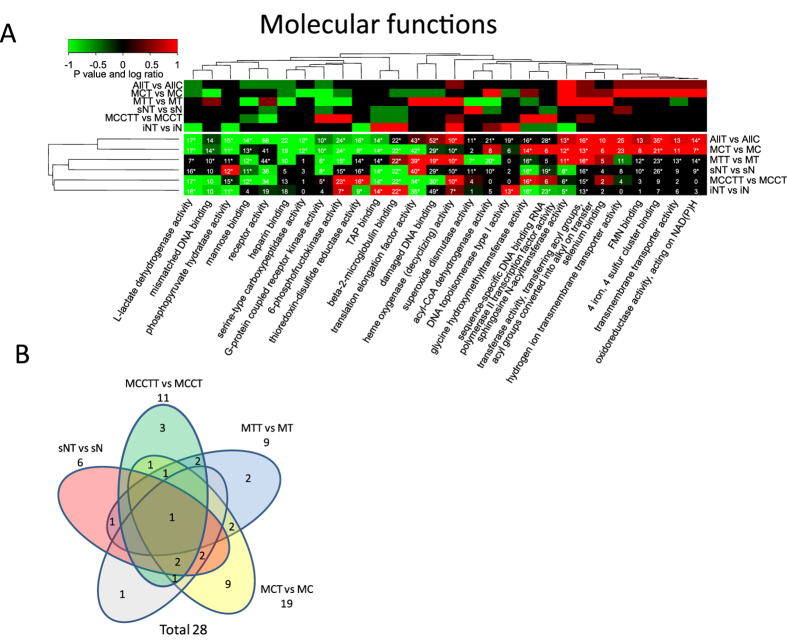
Functional regulation analysis. (**A**) The lower heatmap panel indicates significant up- or down- regulated molecular function from gene ontology categories. P values are log ratio encoded (see methods sections for details). The upper panel indicates the log ratios where color code for log ratio 1 indicates a log ratio > = 1 and for −1 indicates log ratio < = −1. The numbers in each cell indicates the maximum number of proteins identified across all replicas for a specific protein category and subcellular fraction. *) indicate that the functional enrichment analysis is significant (P value <0.05 after FDR correction for multiple testing). (**B**) Venn diagram of the overlap of significant regulated molecular function categories. Overlaps with no numbers indicate zero overlap.

**Figure 6:**
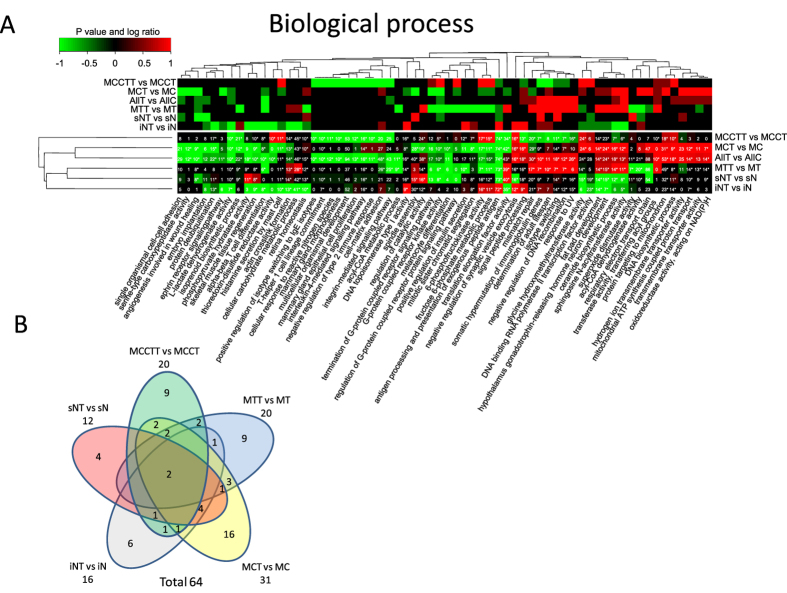
Functional regulation analysis. (**A**) The lower heatmap panel indicates significant up- or down- regulated biological process from gene ontology categories. P values are log ratio encoded (see methods sections for details). The upper panel indicates the log ratios where color code for log ratio 1 indicates a log ratio > = 1 and for −1 indicates log ratio < = −1. (**B**) Venn diagram of the overlap of significant regulated biological process categories. Overlaps with no numbers indicate zero overlap.

Therefore, as an alternative strategy proteins for each functional category can be selected, extracted and the quantitative values for each specific subset of proteins tested for significant regulation by calculating a paired t test or permutation tests (ESA). This leads to fewer statistical tests and subsequently less correction for multiple testing but on the other hand does not capture the enrichment of the functional category. For example, a significant regulated functional category with only two proteins out of ten identified possess less impact compared to a significant regulated functional category with eight out of ten proteins identified. In a similar way a significant regulated functional category with eight out of two hundred proteins does not have the same importance as a significant regulated category with eight out of ten proteins identified. The hypergeometric density function, e.g. using R’s dhyper function or one-tailed Fisher’s exact test, can capture the significance of enrichment of a functional category and takes as input four parameters which are: 1) the number of proteins identified in a functional category, 2) the total number of background proteins in the category, 3) the total number of background proteins outside the category and 4) the total number of identified proteins. We therefore define complete functional enrichment analysis by combining significance testing of regulation within a specific functional category (ESA) with testing for overall functional enrichment (CEA, see methods section for details). Additionally, a small enriched functional category does not have as much impact as a large functional category. For example, consider two proteins out of three in contrast to eleven out of twelve. We therefore also report the maximum number of proteins across all replicas. This combined statistical analysis is then summarized in a single heatmap. The final heatmap displays the P values for entity regulation (ESA), the P values for functional enrichment of all detected proteins in each sample group (CEA), the maximum number of proteins identified across all replicas and the log ratios. The heatmaps in Figs 4, 5, 6A display log ratio encoded P values (P > = 0.95 is significantly upregulated and P < = −0.95 is significantly down regulated molecular functions). The P value is based on a paired t test that compares expression values for all proteins in a specific functional category before and after treatment. The P values are subsequently corrected for multiple testing by FDR and log ratio encoded (see experimental section for details). The integer values in the heatmaps indicate the maximum number of identified proteins for a specific category across all replicas. Stars indicate protein categories that are functional enriched as estimated by the hypergeometric distribution^17^,^18^ (see also methods section).

The resulting heatmap provides a rather informative and concise functional summary of the data. It therefore serves as a starting point to formulate novel hypothesis for further experimental validation. We previously demonstrated cell cycle arrest and to some extent apoptosis upon glucosamine treatment of KMH2 cells^5^. This is well reflected in the significant regulated functional categories e.g. up regulation of “NELF complex” in cytoplasmic and nuclear fractions (causing down-regulation of transcription), general up-regulation of “aggresome” and “inclusion body” (Fig. 4).

However, most of the significant functional categories identified were not detected by the standard functional analysis applied in our previous study where we first identified significant up and down regulated proteins from each fraction followed by functional enrichment analysis^5^. For example, the significant regulation of the functional categories “damaged DNA binding” (Fig. 5), “mismatch repair” (Fig. 6), “negative regulation of DNA recombination” (Fig. 6) and functional categories related to “membrane trafficking” (Figs 5) were not previously identified as significant regulated.

**Figure 7:**
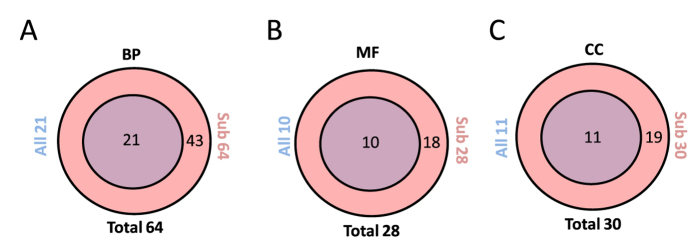
Venn diagrams comparing significant regulated gene ontology categories for all data versus total significant regulated categories for all subcellular fractions. The Venn comparisons were made for (**A**) biological process (BP), (**B**) molecular function (MF) and (**C**) cellular component (CC).

These newly discovered significant regulated functional categories could potentially be involved in the observed glucosamine protection against bortezomib that we have previously reported^5^. With this newly proposed analysis we additionally demonstrated that the outcome of subcellular fractionation results in higher number of significant regulated functional groups compared to the case where all peptides are analyzed globally as one group (Fig. 7).

Merging peptide quantitative data from all subcellular fractions corresponds to what would have been obtained if only peptide fractionation methods were used before LC-MS analysis. It is evident that there are in general small or no overlap between significant regulated functional categories of cellular component from the different subcellular fractions (Figs 4).

To test the robustness of this observation different threshold values for the minimum number of proteins per functional category and the FDR threshold for protein identification were adjusted. More strict as well as less strict criteria resulted in a similar overlap in significant regulated functional categories.

The functional category “mismatch repair” is down regulated in MCCTT and MCT fractions whereas it is significantly up regulated in the MTT fraction (Fig. 6A). These observations can be attributed to the fact that subsets of functional gene ontology groups are compartmentalized. If all peptide data is merged in one large and complex mixture, functional regulation occurring in specific cell compartments would in some cases be averaged out. Comparisons of significant regulated functional categories obtained from all merged data versus all subcellular fractions by Venn diagrams reveals that the methodology based on subcellular fractions identifies a significant number of additional functional regulated categories (Fig. 7).

## Discussion

The above mentioned discoveries provides a good argument for performing subcellular fractionation of protein mixtures in global drug profiling studies with or without subsequent peptide fractionation before LC-MS. It further questions the meaning of quantitative single shotgun proteomics on a whole cell lysate basis. It is well established that cells possess mechanisms to locally regulate protein levels by protein trafficking^19^ and by local translation of mRNAs^20^, a highly dynamic cellular process. Furthermore, Boisvert *et al.*^21^ recently demonstrated that large multiprotein complexes that are assembled in one cellular compartment and function in another, are degraded significantly faster in the assembly compartment than in the functional compartment. Mixing proteins from different cellular compartments will obscure the detection of functional pathways that are regulated at a subcellular level. Moreover, even if mass spectrometry based proteomics reach sensitivity levels sufficient to profile the full proteome of a cell in a single LC-MS analysis the biological inference will suffer from lack of the proteome dynamics at the organelle level.

**Figure 8:**
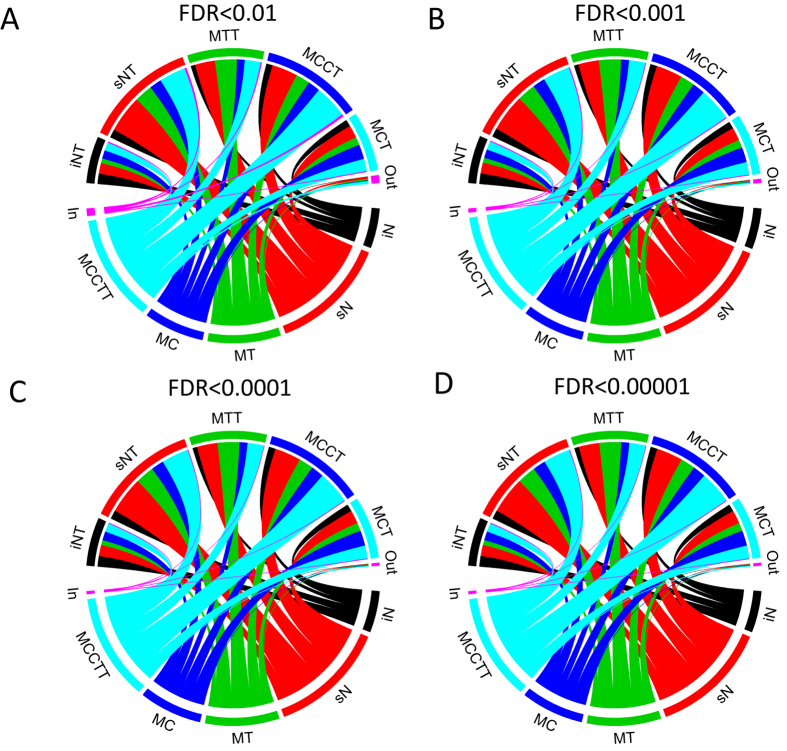
Overlap in identified proteins from the five subcellular fractions before and after exposure to GlcN. “In” indicates proteins identified in the five treated subcellular fractions but not in any of the five untreated subcellular fractions. “Out” indicates proteins identified only in the five untreated fractions but not in any of the five treated subcellular fractions. FDR indicate the false discovery threshold used for protein identification.

We used here as proof of concept subcellular fractionation combined with MS-based label free quantitative proteomics and functional regulation analysis. The method enabled deep proteome coverage, identifying 18889 human protein isoforms which can be collapsed into 6279 unique coding genes. A total of 123836 peptides with unique amino-acid sequence were identified at 1% FDR. Supplementary Table S1 compares these values with a deep profiling approach by Nagaraj *et al.* using both protein and peptide fractionation^11^. Nagaraj *et al.* obtained a deeper profiling by using 72–126 fractions compared to our five subcellular fractions. Our proposed method demonstrates only slightly lower coverage (Supplementary Table S1). Furthermore, the strategy by Nagaraj *et al.* is not compatible with the functional regulation analysis since the fractions created do not reflect subcellular compartments. Nevertheless, the comparison demonstrates that further work is needed to optimize the proteome coverage by subcellular fractionation preferably by a minimal number of fractions. For example, 72 fractions over time and different drug concentrations will be timely and costly. Moreover, the five subcellular fractions resulted in large overlap in identified proteins (Fig. 8).

Four different FDR thresholds for protein identifications were applied to test if these overlaps were a result of low level cross contamination. Yet, the overlap patterns were evident for all FDR thresholds applied (Fig. 8). This result confirms previous findings using three human cell lines where 40% of 4000 genes/proteins were found to localize to multiple cellular compartments^22^. Despite the large overlap in protein content in different subcellular compartments subcellular proteomics were shown to provide more significant regulated functional categories compared to simulated single shotgun proteomics. Moreover, regulation of proteins, participating in multiprotein complexes, common among cellular compartments might constitute distinct processes. Our results presented in Figs 4 supports local regulation of at least a subset of cellular processes. Therefore deep insight into cellular mechanisms in different biological sets such as cancer, infection or response to drugs requires multidimensional approaches (spatial and temporal proteomics) complemented by new computational biological tools^23^.

In conclusion, subcellular fractionation combined with state of art LC-MS and complete functional regulation analysis provides a more detailed insight into functional regulation compared to using current established methodologies. Furthermore, subcellular localization does not in general share significant functional regulation with other subcellular localizations. Moreover, our results indicate that quantification by iBAQ^24^ results in more significant regulated functional categories compared to using spectral counting (result not shown). The proteome coverage by using five subcellular fractions, as outlined here, profiles 31% fewer protein encoding genes compare to previous described deep LC-MS profiling but using only five versus 72 fractions^25^. We envisage that further improvements can be achieved by minimizing the identified protein overlap between subcellular fractions and by improving duty cycle and sensitivity of future MS instruments. Chromatographic separation can be further optimized to obtain deeper protein coverage of each of the subcellular fractions^25^. Efforts on optimizing and comparing subcellular fractionation methods combined with LC-MS are required. Finally, the criteria for defining an optimal subcellular fractionation method will depend on the cell type and the aim of the study. The data analysis strategy demonstrated here could also be used for such comparative studies with the aim to optimize subcellular fractionation for a specific cell type and biological study.

## Methods

### Cell lines and culture conditions

The human Hodgkin Lymphoma derived cell line KMH2 was obtained from the German Collection of Microorganisms and Cell Cultures, Department of Human and Animal Cell Cultures. KMH2 was cultured in Gibco RPMI medium 1640 GlutaMAX™ (Gibco, Invitrogen) supplemented with 10% heat-inactivated FBS (Gibco, Invitrogen) in a humid environment of 5% CO_2_ at 37 °C. For Glucosamine (GlcN) treatment cells were cultured for 24 h and replated at 5 × 10^5^ cells/ml with or without GlcN at 20 mM for 24 h.

### Subcellular Fractionation

Cells were disrupted in ice-cold cell homogenization medium (10 mM Tris, pH 6.7, 150 M MgCl_2_, 10 mM KCl) by passing through a 20G syringe. Cell breakage was examined under a phase-contrast microscope. After addition of ice-cold cell homogenization medium containing 1 M sucrose (final 0.25 M) to the disrupted cells, nuclei were pellet by centrifuging 5 min at 1000× g, 4 °C. To obtain mitochondria the remaining supernatant was centrifuged 10 min at 5000× g, 4 °C and the pellet resuspended in ice-cold sucrose/Mg^2+^ medium (10 mM Tris, pH 6.7, 150 mM MgCl_2_, 0.25 M sucrose). Mitochondria were pellet by recentrifuging the suspension at 5000× g, 10 min, 4 °C. The supernatant was designated MCCT fraction and was further fractionated by ultracentrifugation 60 min at 100000× g, 4 °C. The pellet was used to prepare the microsomal fraction (MC) and the supernatant the cytosolic fraction (CT). MCCT fraction was analyzed without further processing. All samples were stored at −80 °C until use. For further electrophoresis and MS/MS analysis, nuclei pellet was lysed using RIPA lysis buffer at 4 °C for 20 min on ice and the nuclear lysate centrifuged for 20 min at 15000× g, 4 °C. The supernatant constituted the soluble nuclear fraction (sN) and the pellet the insoluble nuclear fraction (iN) which was resuspend in RIPA buffer, incubated 30 minutes on ice, vortexed every 5 min and finally centrifuged 20 minutes, at 15000g, 4 °C. Mitochondria were lysed using RIPA lysis buffer at 4 °C for 20 min and the lysate cleared by centrifuging 20 min at 15000× g, 4 °C, constituting the mitochondrial fraction (MT). The microsomal fraction (MC) was prepared by lysis of the pellet obtained by ultracentrifugation and prepared as described above for the mitochondrial fraction. We used in this study as a proof of concept the five subcellular fractions iN, sN, MC, MT and MCCT. Alternatively, the CT fraction instead of the MCCT fraction and a high salt buffer to prepare the iN fraction could be attempted.

### Peptide sample preparation

Protein solution containing SDS and DTT were loaded into filtering columns and washed exhaustively with 8M urea in HEPES buffer^26^. Proteins were reduced with DTT and alkylated with IAA. Protein digestion was performed by overnight digestion with trypsin sequencing grade (Promega).

### Mass spectrometry

Generated peptides as described above were desalted and concentrated^27^ prior to analysis by nano LC-MS/MS using an Q-Exactive (Thermo, San Jose, CA, USA) mass spectrometer coupled to a Dionex NCP3200RS HPLC setup (Thermo, Sunnyvale, CA, USA). A 75 μm ID, 15 cm in length home build reversed phase column (Reprosil-pur 3um C18-AQ, Ammerbuch-Entringen, Germany) was used to separate peptides. The analytical gradient was generated at 200 nL/min increasing from 5% Buffer B (0.1% formic acid in acetonitrile)/95% Buffer A (0.1% formic acid) to 35% Buffer B/65% Buffer A over 110 minutes followed by an increase to 90% Buffer B/10% Buffer A in 10 minutes.

MS survey scans were scanned from m/z 350 to m/z 1400 at 70.000 resolution (AGC: 1e6 and Maximum IT: 120 ms). An upper limit of 20 most abundant ions was subjected to MS/MS and measured at a resolution of 35.000 (AGC: 5e4 and Maximum IT: 120 ms) with lowest mass set to m/z 100.

### Preprocessing of MS data

Q-Exactive data were calibrated using polycyclodi-methylsiloxane (PCMs—outgassed material from semiconductors) present in the ambient air and Bis(2-Ethylhexyl)(Phthalate) (DEHP—from plastic)^28^,^29^ by modular VEMS^30^. Modular VEMS further allows alternative parent ion annotations for each MS/MS spectrum which is needed if two peptide elution profiles overlap in the m/z and retention time dimension. By allowing alternative parent ion annotation for each MS/MS spectrum, provides a storage space efficient data format. Furthermore these alternative parent ion annotations were taken into account during the database dependent search.

### MS database dependent search

A customized sequence database was established, which includes all common contaminants^31^, genomic variation described by Liu *et al.*^32^ and permutated protein sequences keeping Arg and Lys in place.

All data were searched with VEMS^33^. Mass accuracy was set to 5 ppm for peptides and 10 mDa for peptide fragments. Gaussian weight for fragment ions was set to 5 and the six most intense fragment ions per 100 Da was used for scoring fragment ions. Four missed cleavages were specified and the database UniProtKB/TrEMBL (Release 2015_02) were used including permutated protein sequences, leaving Lys and Arg in place, together with common contaminants such as human keratins, bovine serum proteins and proteases^31^. The total number of protein entries searched was 136314. Fixed modification of carbamidomethyl cysteine was included in the search parameters. A list of 12 variable modifications (Supplementary Table S3) was considered for all data searched against the full protein database. Protein N-terminal Met-loss is not specified for VEMS searches since VEMS by default checks N-terminal Met-loss. The false discovery rate (FDR) for protein identification was set to 1% for peptide and protein identifications unless otherwise specified. No restriction was applied for minimal peptide length. Identified proteins were divided into evidence groups as defined by Matthiesen *et al.*^34^.

Our data were also analyzed using a proteogenomics^35^ strategy by first constructing a protein sequence database which contains genomic variations observed in a recent whole-exome sequencing (WES) publication targeting the Hodgkin lymphoma cell line KMH2^32^. We identified 113 mutated proteins by global MS based profiling out of 376 non redundant genes found to have mutations, insertion or deletions by WES (Supplementary Table S2). A subset of these was identified with peptides that covered the WES observed mutation, insertion or deletion (Supplementary Fig. S1).

### Quantitative proteome analysis

Proteins were quantified by spectral counting^36^ and mziXIC^30^ followed by iBAQ^16^,^24^ estimation. We present only the result from the iBAQ quantitation. The quantitative values were added one and log two transformed. A paired t test was used to test for significant regulation of proteins for each functional category. We used functional categories rather than individual proteins to test for significant regulation. This results in fewer hypothesis tests and consequently less correction of p values. Correction for multiple testing was done by the FDR method^37^ and no imputation for missing values was used. P values were log ratio encoded for heatmap visualization. P values for up regulated functional categories were transformed as 1-p whereas p values for down regulated functional categories were transformed as p-1.

### Complete functional regulation analysis

For simplicity we have used the gene ontology functional categories obtained from UniProt (Release 2015_02): 1) cellular component, 2) biological process and 3) molecular function. We used R hyper geometric functions to estimate significance enrichment of identified proteins by “Complete Entity Analysis”. IEA is frequently used to test for significant enrichment or depletion among regulated genes or proteins. CEA on the other hand is a useful alternative for proteomics studies where sub cellular compartments frequently are biochemically enriched. CEA can for example test significantly enriched entities among the mass spectrometry identified proteins from a subcellular fraction. We have previously provided a more detailed discussion on enrichment analysis based on the hyper geometric function^38^. Briefly, the hyper geometric function’s probability mass function (Eq. 1) is the exact null distribution^39^.


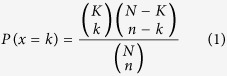


In equation 1, *K* is the number of success states in the population. *K* translates to the number of proteins assigned to a given GO annotation in the sequence database used for searching MSMS data. There is a subtle difference here from IEA since the proteins identified or genes on an array are often used as a reference list for IEA. However, for CEA the most reasonable choice as a reference list are the proteins (entities) in the sequence database used for searching the MSMS data. *n* is the number of draws and *k* is the number of observed successes. *n* translates to the total number of proteins identified in a specific subcellular fraction. *k* translates to proteins assigned to a given GO annotation among the proteins identified in a specific subcellular fraction. *N* is the population size. *N* translates to the total number of entities in the reference list that is the total number of proteins in the sequence database used for searching the MSMS data. Again note the subtle difference in the definition of the reference list between IEA and CEA.

For the presented analysis a one-sided test was calculated by the cumulative density function of the hyper geometric function (Eq. 2). However, other statistical tests have been proposed and a two sided test for both depletion and enrichment could also have been calculated (see Hackenberg *et al.*^38^ for more detailed discussion). The cumulative density function of the hyper geometric function can conveniently be calculated by R’s dhyper function. The cumulative density function of the hyper geometric function in Equation 2 calculates the probability of obtaining between 0 and x proteins in a specific GO category by chance. We define P values for enrichment as the probability to obtain more than x proteins assigned to a specific GO annotation by chance as 1-CDF_x_^40^.


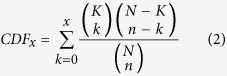


We observed that CEA frequently found GO categories with many entities significant. However, if the significant GO categories found by CEA were filtered by significant regulated GO annotations by “Entity Set Analysis” an informative list of GO categories would be extracted. A paired t test using iBAQ or log transformed spectral counts as input were used for “Entity Set Analysis”. We have previously published more extensive approaches using large database of functional categories and combination of categories^18^. Furthermore, functional analysis can be extended to include not only qualitative analysis but also quantitative analysis^17^. Heatmaps were generated by using the R package “heatmap.3”. Venn diagrams were created using R package “VennDiagram”. Chord diagrams were created with the R package “circlize”.

## Additional Information

**How to cite this article**: Sofia Carvalho, A. *et al.* New insights into functional regulation in MS-based drug profiling. *Sci. Rep.* **6**, 18826; doi: 10.1038/srep18826 (2016).

## Supplementary Material

Supplementary Information

## Figures and Tables

**Figure 1:**
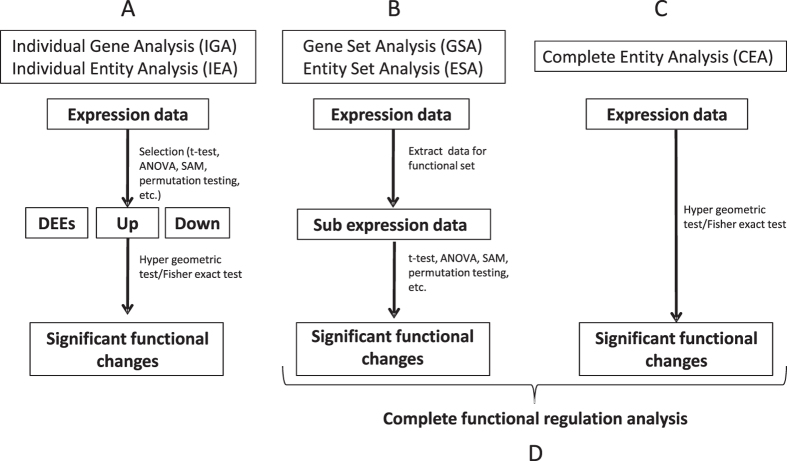
Overview of basic types of functional analysis. (**A**) Individual Entity Analysis (IEA) uses firstly a statistical test to define a set of up- and/or down-regulated entities (differentially expressed entities, DEEs). In the second step a hyper geometric or Fisher Exact test is used to test for over or under representation of entities for each functional set. (**B**) Entity Set Analysis (ESA) for each functional set a sub expression data matrix for only the entities in the set is created. Next a statistical test applied to the sub-matrix enquires if the functional set is significantly regulated across sample groups. (**C**) Complete Entity Analysis (CEA) extracts entities identified (positive expression detected) across samples. For each sample type a Hypergeometric test or Fischer Exact test is used to test for over or under representation of entities for each functional set. (**D**) Complete functional regulation analysis performs the analysis in B and C to generate a visual display that illustrates and summarize information from both types of methods.

## References

[b1] Zentner G. E. & Henikoff S. High-resolution digital profiling of the epigenome. Nature reviews. Genetics 15, 814–827 (2014). 10.1038/nrg3798 25297728

[b2] Rodriguez R. & Miller K. M. Unravelling the genomic targets of small molecules using high-throughput sequencing. Nature reviews. Genetics 15, 783–796 (2014). 10.1038/nrg3796 25311424

[b3] Schirle M., Bantscheff M. & Kuster B. Mass spectrometry-based proteomics in preclinical drug discovery. Chemistry & biology 19, 72–84 (2012). 22284356 10.1016/j.chembiol.2012.01.002

[b4] Watkins S. M. & German J. B. Metabolomics and biochemical profiling in drug discovery and development. Current opinion in molecular therapeutics 4, 224–228 (2002). 12139307

[b5] Carvalho A. S. *et al.* Global mass spectrometry and transcriptomics array based drug profiling provides novel insight into glucosamine induced endoplasmic reticulum stress. Molecular & cellular proteomics: MCP 13, 3294–3307 (2014). 25128556 10.1074/mcp.M113.034363 PMC4256484

[b6] Graumann J. *et al.* Applicability of tandem affinity purification MudPIT to pathway proteomics in yeast. Molecular & cellular proteomics: MCP 3, 226–237 (2004). 14660704 10.1074/mcp.M300099-MCP200

[b7] Schirmer E. C., Yates J. R. 3rd & Gerace L. MudPIT: A powerful proteomics tool for discovery. Discovery medicine 3, 38–39 (2003). 20704860

[b8] Branca R. M. *et al.* HiRIEF LC-MS enables deep proteome coverage and unbiased proteogenomics. Nature methods 11, 59–62 (2014). 24240322 10.1038/nmeth.2732

[b9] Lasonder E. *et al.* Analysis of the Plasmodium falciparum proteome by high-accuracy mass spectrometry. Nature 419, 537–542 (2002). 12368870 10.1038/nature01111

[b10] Ahmad Y., Boisvert F. M., Lundberg E., Uhlen M. & Lamond A. I. Systematic analysis of protein pools, isoforms, and modifications affecting turnover and subcellular localization. Molecular & cellular proteomics: MCP 11, M111 013680 (2012). 22002106 10.1074/mcp.M111.013680 PMC3316725

[b11] Nagaraj N. *et al.* Deep proteome and transcriptome mapping of a human cancer cell line. Molecular systems biology 7, 548 (2011). 22068331 10.1038/msb.2011.81 PMC3261714

[b12] Huang D. W. *et al.* DAVID Bioinformatics Resources: expanded annotation database and novel algorithms to better extract biology from large gene lists. Nucleic acids research 35, W169–175 (2007). 17576678 10.1093/nar/gkm415 PMC1933169

[b13] Nam D. & Kim S. Y. Gene-set approach for expression pattern analysis. Briefings in bioinformatics 9, 189–197 (2008). 18202032 10.1093/bib/bbn001

[b14] Huang da W., Sherman B. T.& Lempicki R. A. Systematic and integrative analysis of large gene lists using DAVID bioinformatics resources. Nature protocols 4, 44–57 (2009). 19131956 10.1038/nprot.2008.211

[b15] Graham J. M. Isolation of mitochondria from tissues and cells by differential centrifugation. Current protocols in cell biology. (2001). 10.1002/0471143030.cb0303s04 18228355

[b16] Schwanhausser B. *et al.* Global quantification of mammalian gene expression control. Nature 473, 337–342 (2011). 21593866 10.1038/nature10098

[b17] Hackenberg M., Lasso G. & Matthiesen R. ContDist: a tool for the analysis of quantitative gene and promoter properties. BMC bioinformatics 10, 7 (2009). 19128472 10.1186/1471-2105-10-7 PMC2631519

[b18] Hackenberg M. & Matthiesen R. Annotation-Modules: a tool for finding significant combinations of multisource annotations for gene lists. Bioinformatics 24, 1386–1393 (2008). 18434345 10.1093/bioinformatics/btn178

[b19] Flintoft L. Signalling: Transcription factors tune In. Nature reviews. Genetics 14, 154–155 (2013). 10.1038/nrg3431 23381118

[b20] Kim-Ha J., Kerr K. & Macdonald P. M. Translational regulation of oskar mRNA by bruno, an ovarian RNA-binding protein, is essential. Cell 81, 403–412 (1995). 7736592 10.1016/0092-8674(95)90393-3

[b21] Boisvert F. M. *et al.* A quantitative spatial proteomics analysis of proteome turnover in human cells. Molecular & cellular proteomics: MCP 11, M111 011429 (2012). 21937730 10.1074/mcp.M111.011429 PMC3316722

[b22] Fagerberg L. *et al.* Mapping the subcellular protein distribution in three human cell lines. Journal of proteome research 10, 3766–3777 (2011). 21675716 10.1021/pr200379a

[b23] Larance M. & Lamond A. I. Multidimensional proteomics for cell biology. Nature reviews. Molecular cell biology 16, 269–280 (2015). 25857810 10.1038/nrm3970

[b24] Mann K. & Edsinger E. The Lottia gigantea shell matrix proteome: re-analysis including MaxQuant iBAQ quantitation and phosphoproteome analysis. Proteome science 12, 28 (2014). 25018669 10.1186/1477-5956-12-28 PMC4094399

[b25] Thakur S. S. *et al.* Deep and highly sensitive proteome coverage by LC-MS/MS without prefractionation. Molecular & cellular proteomics: MCP 10, M110 003699 (2011). 21586754 10.1074/mcp.M110.003699 PMC3149084

[b26] Wisniewski J. R., Zougman A., Nagaraj N. & Mann M. Universal sample preparation method for proteome analysis. Nature methods 6, 359–362 (2009). 19377485 10.1038/nmeth.1322

[b27] Rappsilber J., Mann M. & Ishihama Y. Protocol for micro-purification, enrichment, pre-fractionation and storage of peptides for proteomics using StageTips. Nature protocols 2, 1896–1906 (2007). 17703201 10.1038/nprot.2007.261

[b28] Schlosser A. & Volkmer-Engert R. Volatile polydimethylcyclosiloxanes in the ambient laboratory air identified as source of extreme background signals in nanoelectrospray mass spectrometry. J. Mass Spectrom. 38, 523–525 (2003). 12794873 10.1002/jms.465

[b29] Olsen J. V. *et al.* Parts per million mass accuracy on an Orbitrap mass spectrometer via lock mass injection into a C-trap. Molecular & cellular proteomics: MCP 4, 2010–2021 (2005). 16249172 10.1074/mcp.T500030-MCP200

[b30] Matthiesen R. LC-MS spectra processing. Methods Mol Biol 1007, 47–63 (2013). 23666721 10.1007/978-1-62703-392-3_2

[b31] Bunkenborg J., Garcia G. E., Paz M. I., Andersen J. S. & Molina H. The minotaur proteome: avoiding cross-species identifications deriving from bovine serum in cell culture models. Proteomics 10, 3040–3044 (2010). 20641139 10.1002/pmic.201000103

[b32] Liu Y. *et al.* The mutational landscape of Hodgkin lymphoma cell lines determined by whole-exome sequencing. Leukemia 28, 2248–2251 (2014). 24947018 10.1038/leu.2014.201

[b33] Matthiesen R. Algorithms for database-dependent search of MS/MS data. Methods Mol Biol 1007, 119–138 (2013). 23666724 10.1007/978-1-62703-392-3_5

[b34] Matthiesen R. *et al.* SIR: Deterministic protein inference from peptides assigned to MS data. Journal of proteomics 75, 4176–4183 (2012). 22626983 10.1016/j.jprot.2012.05.010

[b35] Nesvizhskii A. I. Proteogenomics: concepts, applications and computational strategies. Nature methods 11, 1114–1125 (2014). 25357241 10.1038/nmeth.3144 PMC4392723

[b36] Matthiesen R. & Carvalho A. S. Methods and algorithms for quantitative proteomics by mass spectrometry. Methods Mol Biol 1007, 183–217 (2013). 23666727 10.1007/978-1-62703-392-3_8

[b37] Benjamini Y. & Hochberg Y. Controlling the false discovery rate: a practical and powerful approach to multiple testing. *J. Roy. Stat. Soc.* B 57, 289–300 (1995).

[b38] Hackenberg M. & Matthiesen R. Algorithms and methods for correlating experimental results with annotation databases. Methods Mol Biol 593, 315–340 (2010). 19957156 10.1007/978-1-60327-194-3_15

[b39] Rivals I., Personnaz L., Taing L. & Potier M. C. Enrichment or depletion of a GO category within a class of genes: which test? Bioinformatics (Oxford, England) 23, 401–407 (2007). 10.1093/bioinformatics/btl633 17182697

[b40] Draghici S., Khatri P., Martins R. P., Ostermeier G. C. & Krawetz S. A. Global functional profiling of gene expression. Genomics 81, 98–104 (2003). 12620386 10.1016/s0888-7543(02)00021-6

