# Supplementary material for: New insights into functional regulation in MS-based drug profiling
Source: Sci Rep. 2016 Jan 8;6:18826. doi: 10.1038/srep18826 (PMC4705526; doi:10.1038/srep18826)
Supplement: Supplementary Information [file srep18826-s1.pdf]

New insights into functional regulation in MS-based drug profiling

Ana Sofia Carvalho[1], *Henrik Molina*[2] *and Rune Matthiesen*[1]

1) Computational and Experimental Biology Group, National Health Institute Dr. Ricardo Jorge, IP, Av. Padre Cruz, 1649-016, Lisbon, Portugal
2) Proteomics Resource Center, The Rockefeller University, 1230 York Avenue, New York, New York 10065-6399

Supplementary information tables

#spectra	Protein coding genes	Unique peptides	#Fractions	Strategy
2337336	9207	166420/NA	72	Protein and peptide fractionation
*570986/580359	6279	123836/176403	5	Subcellular fractionation

*) control/treatment

Table S1. Coverage comparison by using 5 subcellular protein fractions compared to 72 fractions obtained by protein gel filtration and strong anion exchange. Unique peptides: Unique canonical peptides/unique peptides considering modifications.

Table S2. Proteins identified in the cell line KMH2 found to be mutated by WES. Green indicates proteins for which identified peptides covered the amino acid changes.

GENE	POS	AA_CHANGE	CODON_CHANGE
CDK11A	1647893	-117KK	-/AAGAAA
RPL22	6253022	KI69N	aagatc/aac
MTOR	11182151	T2232I	aCc/aTc
RPS6KA1	26887264	K329N	aaG/aaT
RRAGC	39322706	T96A	Acc/Gcc
DNAJC6	65878649	M882L	Atg/Ctg
ODF2L	86814453	Y508N	Tat/Aat
CTSS	1.51E+08	Q100K	Cag/Aag
NDUFS2	1.61E+08	R35Q	cGg/cAg
EPRS	2.2E+08	V1305L	Gtg/Ttg
BROX	2.23E+08	Y249H	Tat/Cat
DNAH14	2.25E+08	M1021I	atG/atA
NBAS	15326982	R2079*	Aga/Tga
ATL2	38526412	K225E	Aaa/Gaa
MOGS	74689399	R400Q	cGa/cAa
RANBP2	1.09E+08	E1667K	Gag/Aag
R3HDM1	1.36E+08	V743E	gTa/gAa
CCDC148	1.59E+08	D436A	gAt/gCt
TANC1	1.6E+08	A1465V	gCt/gTt
SPATS2L	2.01E+08	R366W	Cgg/Tgg
NCL	2.32E+08	ED243D	gaggac/gac
ILKAP	2.39E+08	A54T	Gct/Act
RAF1	12650410	F146I	Ttc/Atc
UBE2E2	23250230	T14A	Act/Gct
FOXP1	71101725	L158P	cTt/cCt
CEP70	1.38E+08	K162I	aAa/aTa
TBL1XR1	1.77E+08	N212D	Aat/Gat
GAK	843751	A1176T	Gcc/Acc
GRK4	2965776	P23PA	ccg/cCGGcg
N4BP2	40103810	K115N	aaA/aaC
PDHA2	96761473	E58*	Gag/Tag
METAP1	99982425	R103P	cGg/cCg
LEF1	1.09E+08	P44A	Ccc/Gcc
SPATA5	1.24E+08	Y324F	tAt/tTt
TRIM60	1.66E+08	NA	NA
WDR70	37703121	S450R	Agc/Cgc
IPO11	61811219	NA	NA
SLC25A46	1.1E+08	V12L	Gtg/Ttg
VARS	31760561	G212R	Gga/Aga

MLIP	54002549	S509C	tCt/tGt
AEBP1	44150627	NA	NA
AGAP3	1.51E+08	R116C	Cgc/Tgc
MCM4	48879968	T326I	aCa/aTa
MRPS28	80942300	L62F	Ctt/Ttt
PAG1	81899679	S67N	aGc/aAc
ESRP1	95680368	VL234V	gtcctc/gtc
PLEC	1.45E+08	A396V	gCa/gTa
IKBKAP	1.12E+08	L252F	ttG/ttT
NDUFA8	1.25E+08	R166C	Cgc/Tgc
C9orf114	1.32E+08	M210I	atG/atT
NUP214	1.34E+08	R19G	Cgt/Ggt
NEBL	21102942	K758Q	Aaa/Caa
MTPAP	30653977	R69*	Cga/Tga
ADO	64565293	Q158H	caG/caT
DDX50	70706238	R689Q	cGg/cAg
TACC2	1.24E+08	P145L	cCc/cTc
TACC2	1.24E+08	G696R	Gga/Aga
MKI67	1.3E+08	M1245I	atG/atA
MKI67	1.3E+08	K219Q	Aag/Cag
RRP8	6621798	V390A	gTg/gCg
ADRBK1	67053984	S406R	Agt/Cgt
SERPINH1	75283010	K163R	aAg/aGg
DLAT	1.12E+08	T335A	Acg/Gcg
RNF214	1.17E+08	D221G	gAt/gGt
RACGAP1	50386391	G199S	Ggt/Agt
TFCP2	51501107	Q149R	cAa/cGa
KRT3	53189427	A134P	Gct/Cct
PPP1R12A	80239112	NA	NA
PPFIA2	81769676	E245K	Gaa/Aaa
TRAFD1	1.13E+08	R392G	Cgc/Ggc
MED13L	1.16E+08	L699S	tTg/tCg
MSI1	1.21E+08	NA	NA
GTF2H3	1.24E+08	D119H	Gac/Cac
SUPT16H	21834637	Q336L	cAg/cTg
GTF2A1	81651931	E284K	Gag/Aag
SETD3	99865086	Q572P	cAa/cCa
PDIA3	44048957	R119S	agG/agT
SPG11	44955780	M22I	atG/atC
ZWILCH	66819637	D163N	Gat/Aat
UNC45A	91485808	R262*	Cga/Tga
SRRM2	2812325	P564L	cCc/cTc
IL32	3117390	D10N	Gac/Aac

TOX3	52553371	G8R	Gga/Aga
ZC3H18	88675410	E269G	gAg/gGg
CTU2	88781603	Q411E	Cag/Gag
CRLF3	29119591	NA	NA
MYO19	34881120	K118R	aAg/aGg
CCR7	38711974	V53L	Gtg/Ctg
VEZF1	56060231	V186A	gTg/gCg
ACOX1	73945843	NA	NA
TUBB6	12326054	NA	NA
CHAF1A	4429530	R567Q	cGg/cAg
ZNF266	9524861	K247R	aAa/aGa
ZNF426	9639298	NA	NA
KRI1	10668876	E414K	Gag/Aag
DNAJB1	14626865	L204F	Ctc/Ttc
MYO9B	17270248	T458M	aCg/aTg
PRKD2	47193937	V577M	Gtg/Atg
GLTSCR2	48259952	R246C	Cgc/Tgc
POLD1	50910318	NA	NA
ZNF841	52570653	N161S	aAc/aGc
ZNF880	52888104	T424N	aCt/aAt
ABHD12	25320300	G5V	gGg/gTg
CHD6	40045932	G2062A	gGa/gCa
ZMYND8	45848924	S1005A	Tcc/Gcc
GNAS	57429554	NA	NA
ARFGAP1	61916271	E144D	gaG/gaC
USP16	30422411	NA	NA
CLTCL1	19220985	G443E	gGg/gAg
TRMT2A	20102135	G399R	Ggg/Agg
RANBP1	20114107	G151C	Ggc/Tgc
PRR14L	32110550	S1092F	tCc/tTc
TCF20	42610618	G232R	Ggc/Cgc
MED14	40569353	T351A	Aca/Gca
SMC1A	53438775	N163S	aAt/aGt
C1orf222	1918415	V119E	gTg/gAg
MEGF6	3417867	P669T	Ccc/Acc
ZNF436	23688470	T301A	Acg/Gcg
KIAA1522	33235862	P302L	cCg/cTg
KIAA1522	33235891	R312C	Cgc/Tgc
ZMYM6	35454289	N798K	aaC/aaA
WDR65	43692750	E988*	Gaa/Taa
PTPRF	44056825	G378S	Ggc/Agc
SLC6A9	44482799	G3S	Ggc/Agc
CYP2J2	60359330	Q501P	cAg/cCg

ROR1	64608324	P389S	Cca/Tca
RPE65	68904763	V287G	gTt/gGt
SAMD13	84853997	C35Y	tGc/tAc
S1PR1	1.02E+08	S445C	tCt/tGt
VAV3	1.08E+08	K552*	Aag/Tag
GPR61	1.1E+08	R140C	Cgc/Tgc
CD58	1.17E+08	NA	NA
SPAG17	1.19E+08	T1016A	Act/Gct
NOTCH2	1.21E+08	G548S	Ggt/Agt
LOR	1.53E+08	G108A	gGg/gCg
SLC27A3	1.54E+08	G18R	Ggg/Agg
PBXIP1	1.55E+08	K481T	aAg/aCg
LY9	1.61E+08	V14E	gTg/gAg
GPA33	1.67E+08	S206L	tCg/tTg
FMO1	1.71E+08	P83S	Cca/Tca
C1orf9	1.73E+08	NA	NA
PAPPA2	1.77E+08	D611N	Gac/Aac
CACNA1E	1.82E+08	Q337P	cAg/cCg
IGFN1	2.01E+08	A568V	gCt/gTt
TNNT2	2.01E+08	NA	NA
PLXNA2	2.08E+08	R493C	Cgc/Tgc
HHAT	2.11E+08	A232V	gCg/gTg
USH2A	2.16E+08	R2677M	aGg/aTg
USH2A	2.16E+08	L2022V	Ttg/Gtg
CHRM3	2.4E+08	S227R	Agt/Cgt
C1orf101	2.45E+08	R723H	cGt/cAt
KIF26B	2.46E+08	A1034V	gCc/gTc
TPO	1544638	R849K	aGg/aAg
AC010872.2	21361946	L536*	tTa/tAa
AC010872.2	21362506	S723T	Tcc/Acc
KIF3C	26203285	R307Q	cGg/cAg
AGBL5	27277596	R217Q	cGa/cAa
BIRC6	32770923	T4269S	aCt/aGt
FEZ2	36805745	G129S	Ggc/Agc
NRXN1	50733732	NA	NA
CCDC142	74709244	T240-	acg/-
CNNM4	97427233	NA	NA
UNC50	99226317	Y32C	tAc/tGc
LONRF2	1.01E+08	G437W	Ggg/Tgg
PSD4	1.14E+08	S436P	Tcg/Ccg
EPB41L5	1.21E+08	V547G	gTc/gGc
BAZ2B	1.6E+08	I117V	Atc/Gtc
ITGB6	1.61E+08	NA	NA

FAP	1.63E+08	G602E	gGa/gAa
FAP	1.63E+08	G602R	Gga/Aga
XIRP2	1.68E+08	NA	NA
HOXD9	1.77E+08	NA	NA
HOXD1	1.77E+08	E159V	gAa/gTa
HOXD1	1.77E+08	P160R	cCc/cGc
TTN	1.79E+08	T21768P	Aca/Cca
TTN	1.79E+08	A21533T	Gcc/Acc
TTN	1.79E+08	W12737R	Tgg/Cgg
TTN	1.8E+08	E5313*	Gaa/Taa
TTN	1.8E+08	T2042A	Aca/Gca
FSIP2	1.87E+08	D5591Y	Gat/Tat
RAPH1	2.04E+08	NA	NA
CXCR2	2.19E+08	C196Y	tGc/tAc
PID1	2.3E+08	E158G	gAg/gGg
GRM7	6903151	C26G	Tgc/Ggc
SLC6A11	10980068	EK627E	gagaag/gag
C3orf20	14813628	E728D	gaG/gaT
DAZL	16679466	I15L	Att/Ctt
XIRP1	39230110	R276Q	cGg/cAg
TMEM42	44905761	S89R	Agc/Cgc
CSPG5	47619289	T76K	aCg/aAg
APPL1	57301785	G620R	Gga/Aga
C3orf67	58834975	S165Y	tCc/tAc
PLXND1	1.29E+08	E1827D	gaG/gaC
PPP2R3A	1.36E+08	NA	NA
DZIP1L	1.38E+08	E622G	gAg/gGg
DZIP1L	1.38E+08	E622*	Gag/Tag
XRN1	1.42E+08	H66Y	Cac/Tac
SHOX2	1.58E+08	L177V	Ctc/Gtc
IFT80	1.6E+08	Q631R	cAa/cGa
SAMD7	1.7E+08	G376R	Ggg/Agg
CCDC39	1.8E+08	L14S	tTa/tCa
MUC4	1.96E+08	N359S	aAt/aGt
TNK2	1.96E+08	P520L	cCg/cTg
CPEB2	15004584	E96V	gAg/gTg
FAM200B	15688673	V25I	Gtt/Att
PCDH7	30725010	S609R	Agc/Cgc
AFM	74364905	V455E	gTg/gAg
CCDC158	77274417	K768N	aaA/aaT
CCDC158	77292577	K381R	aAg/aGg
PTPN13	87735652	T2278I	aCa/aTa
DSPP	88536402	NS863N	aatagt/aat

C4orf37	98108959	R163L	cGt/cTt
KIAA1109	1.23E+08	H2212Y	Cac/Tac
FAT4	1.26E+08	R1509W	Cgg/Tgg
FAT4	1.26E+08	N1754K	aaT/aaG
FREM3	1.44E+08	G2091R	Gga/Aga
RP11-6L6.2	1.47E+08	NA	NA
DCHS2	1.55E+08	T1133I	aCa/aTa
GUCY1B3	1.57E+08	L304R	cTc/cGc
TDO2	1.57E+08	NA	NA
FAM198B	1.59E+08	H130N	Cat/Aat
RAPGEF2	1.6E+08	G75C	Ggt/Tgt
FAT1	1.88E+08	D1280Y	Gat/Tat
IL7R	35876182	Q325R	cAa/cGa
RICTOR	38960526	NA	NA
HEATR7B2	41049398	NA	NA
GHR	42695138	L107Q	cTa/cAa
RAD17	68710083	I495-	ata/-
RP11-428C6.1	73128202	S355L	tCg/tTg
RP11-428C6.1	73128222	R362W	Cgg/Tgg
GPR98	89925278	E587D	gaG/gaC
SLCO4C1	1.02E+08	L434R	cTc/cGc
DMXL1	1.18E+08	P1478L	cCg/cTg
CHSY3	1.3E+08	S600Y	tCt/tAt
PCDHA1	1.4E+08	A100V	gCg/gTg
PCDHB14	1.41E+08	E193K	Gaa/Aaa
PCDHGC4	1.41E+08	L676I	Ctt/Att
PDE6A	1.49E+08	F68C	tTt/tGt
EBF1	1.59E+08	P180T	Cca/Aca
EBF1	1.59E+08	C170G	Tgt/Ggt
GABRA6	1.61E+08	S368P	Tct/Cct
FAM65B	24840973	N601H	Aat/Cat
OR12D3	29342245	A274T	Gcc/Acc
C6orf47	31627004	A241T	Gcc/Acc
GGNBP1	33554498	R50H	cGc/cAc
ITPR3	33633678	N492K	aaT/aaA
UBR2	42571338	H182N	Cat/Aat
TNFRSF21	47252087	D277G	gAc/gGc
KCNQ5	73904505	G613S	Ggc/Agc
EPHA7	94120395	V219A	gTg/gCg
KLHL32	97512587	P3S	Cca/Tca
SOBP	1.08E+08	K286*	Aaa/Taa

SOBP	1.08E+08	P409Q	cCg/cAg
AKD1	1.1E+08	V295-	gtt/-
AKD1	1.1E+08	NA	NA
LAMA4	1.12E+08	D763V	gAc/gTc
DSE	1.17E+08	S806*	tCa/tGa
FAM184A	1.19E+08	T208M	aCg/aTg
LAMA2	1.3E+08	T2015I	aCt/aTt
ARG1	1.32E+08	I169M	atA/atG
TAAR6	1.33E+08	L103I	Ctc/Atc
NMBR	1.42E+08	Y50F	tAc/tTc
GRM1	1.47E+08	L763H	cTc/cAc
SYNE1	1.53E+08	S2733I	aGc/aTc
SYNE1	1.53E+08	K433E	Aag/Gag
SCAF8	1.55E+08	H301R	cAt/cGt
PDE10A	1.66E+08	A625V	gCt/gTt
CHST12	2472977	L235V	Ctc/Gtc
CARD11	2956982	D882V	gAc/gTc
CARD11	2978435	SD297-	tcagac/-
TNRC18	5427366	S697G	Agt/Ggt
THSD7A	11582595	K535E	Aaa/Gaa
SCIN	12610426	L5Q	cTa/cAa
DNAH11	21742390	K2088N	aaA/aaT
STK31	23871902	K970*	Aaa/Taa
ABCA13	48431553	G3897E	gGa/gAa
EGFR	55273148	W1027C	tgG/tgC
PHTF2	77539616	S184R	agT/agG
PCLO	82545928	K3792E	Aag/Gag
PCLO	82586082	S1327C	tCt/tGt
AC093799.1	97937000	NA	NA
MUC3A	1.01E+08	G324E	gGa/gAa
SLC26A3	1.07E+08	NA	NA
DNAJB9	1.08E+08	H197Y	Cat/Tat
HIPK2	1.39E+08	Y468N	Tac/Aac
ABCB8	1.51E+08	R50W	Cgg/Tgg
DLGAP2	1497562	L235F	Ctc/Ttc
MYOM2	2033478	P534T	Ccc/Acc
RP1L1	10480295	NA	NA
EFHA2	16927217	L186F	Ctt/Ttt
RB1CC1	53570015	T792A	Act/Gct
LRRCC1	86027736	E190K	Gag/Aag
CSMD3	1.13E+08	L2824F	ttA/ttT
FER1L6	1.25E+08	K1322Q	Aag/Cag
ZFAT	1.36E+08	C692F	tGt/tTt

ZNF623	1.45E+08	C57F	tGc/tTc
GPT	1.46E+08	E482Q	Gag/Cag
C9orf68	4629109	FL136L	tttctg/ttg
GLDC	6644630	K106N	aaA/aaT
IZUMO3	24544197	NA	NA
IZUMO3	24545911	L12M	Ttg/Atg
TAF1L	32635573	R2Q	cGa/cAa
C9orf128	35825873	NA	NA
C9orf128	35825878	L94S	tTg/tCg
DCAF10	37819330	NA	NA
C9orf41	77598693	K407T	aAg/aCg
TLE4	82321780	S276T	aGt/aCt
S1PR3	91617035	R307Q	cGg/cAg
PTPN3	1.12E+08	P174L	cCt/cTt
TNC	1.18E+08	R1461C	Cgc/Tgc
NOTCH1	1.39E+08	G2436*	Gga/Tga
COBRA1	1.4E+08	F50S	tTc/tCc
C10orf108	696868	NA	NA
FRMD4A	13702440	R577G	Cga/Gga
SPAG6	22634840	R72C	Cgc/Tgc
LYZL2	30918549	A29G	gCg/gGg
CREM	35484138	D7E	gaC/gaA
C10orf71	50532227	N546S	aAt/aGt
KCNK18	1.19E+08	V257G	gTg/gGg
CLRN3	1.3E+08	II198I	atcatt/att
IRF7	613311	W272R	Tgg/Cgg
BRSK2	1481780	E680Q	Gag/Cag
OR52K1	4510149	T7A	Acc/Gcc
SMPD1	6413157	H15Y	Cac/Tac
HPX	6462138	S19Y	tCt/tAt
OTOG	17655346	E1416K	Gag/Aag
GAS2	22770767	S192Y	tCt/tAt
LGR4	27395523	L394V	Cta/Gta
AMBRA1	46564281	R339H	cGc/cAc
OR5D18	55587707	NA	NA
OR8J1	56128476	Y252H	Tat/Cat
SERPING1	57367677	P126L	cCt/cTt
SLC22A8	62762068	A265T	Gct/Act
SLC22A25	62996878	L83M	Ctg/Atg
BBS1	66297398	R354Q	cGa/cAa
LRTOMT	71819070	P114S	Ccc/Tcc
ATG16L2	72536390	N178S	aAc/aGc
BIRC3	1.02E+08	L404R	cTa/cGa

MMP7	1.02E+08	M135K	aTg/aAg
EXPH5	1.08E+08	G282E	gGa/gAa
DRD2	1.13E+08	N17K	aaC/aaA
MLL	1.18E+08	T1478S	Acc/Tcc
OR10G9	1.24E+08	P261Q	cCa/cAa
TIRAP	1.26E+08	P202T	Cct/Act
IQSEC3	283937	I1096T	aTt/aCt
C1RL	7249430	A341T	Gcc/Acc
C3AR1	8211666	Q372H	caG/caC
CLEC12A	10131960	NA	NA
PIK3C2G	18435085	F24L	Ttt/Ctt
CAPZA3	18891824	D208N	Gac/Aac
PLEKHA5	19498783	I545M	atA/atG
SLCO1B3	21069068	M666V	Atg/Gtg
IFLTD1	25699453	E116K	Gaa/Aaa
CNTN1	41302293	A20V	gCa/gTa
CNTN1	41330600	E324K	Gaa/Aaa
ESPL1	53664165	Y383H	Tat/Cat
SUOX	56397514	F114S	tTt/tCt
SUOX	56397944	D92N	Gat/Aat
LRIG3	59282609	M205I	atG/atA
HELB	66698770	K149N	aaG/aaC
OTOGL	80604642	S23L	tCg/tTg
C12orf26	82850538	C504Y	tGt/tAt
LRRIQ1	85492697	N1020S	aAc/aGc
LRRIQ1	85638602	D1684E	gaC/gaA
NR2C1	95416072	V582A	gTt/gCt
ANKS1B	1E+08	S45C	Agc/Tgc
C12orf48	1.03E+08	L295*	tTa/tGa
RFX4	1.07E+08	NA	NA
CABP1	1.21E+08	NA	NA
TMEM132D	1.3E+08	NA	NA
P2RX2	1.33E+08	G242E	gGg/gAg
ZMYM5	20409737	M367I	atG/atA
GJB6	20797064	T186A	Aca/Gca
SACS	23905467	G3433E	gGg/gAg
SMAD9	37446960	P169S	Cca/Tca
POSTN	38144803	P671L	cCa/cTa
FREM2	39264812	T1111S	Act/Tct
ATP7B	52536025	A521T	Gcc/Acc
NEK3	52710309	T350P	Aca/Cca
PCDH9	67800719	F618L	ttT/ttA
EDNRB	78492532	N149K	aaC/aaA

ERCC5	1.03E+08	L16M	Ttg/Atg
COL4A1	1.11E+08	G151D	gGc/gAc
ADPRHL1	1.14E+08	NA	NA
ABHD4	23072577	R132Q	cGg/cAg
MDGA2	47504272	S289R	agC/agG
C14orf183	50550631	P238L	cCc/cTc
SOS2	50605431	L920V	Tta/Gta
NIN	51273478	N43S	aAt/aGt
C14orf148	77873073	P226S	Cct/Tct
CKB	1.04E+08	A9S	Gca/Tca
GABRG3	27773109	P365S	Cct/Tct
TJP1	30112735	S12C	Agt/Tgt
TRPM1	31341645	L480Q	cTg/cAg
BUB1B	40500900	S574L	tCg/tTg
SPTBN5	42143332	A3587V	gCc/gTc
SPTBN5	42168746	Q1317H	caG/caC
SPTBN5	42171536	C1035Y	tGc/tAc
TGM7	43571389	E589K	Gag/Aag
SHF	45470504	L102V	Cta/Gta
SECISBP2L	49304003	L552V	Ttg/Gtg
UNC13C	54307143	N681K	aaC/aaG
UNC13C	54914575	T2051S	Act/Tct
SH2D7	78384999	E25K	Gag/Aag
CHRNA5	78882852	NA	NA
AC022748.1	79045564	E81K	Gag/Aag
DECR2	461507	G258W	Ggg/Tgg
CACNA1H	1257309	Y981F	tAc/tTc
UNKL	1416241	*183W	tgA/tgG
UNKL	1445785	A397V	gCg/gTg
TRAF7	2223235	R283C	Cgc/Tgc
ADCY9	4163828	L539*	tTa/tGa
ERCC4	14029408	S529L	tCg/tTg
IQCK	19746709	F137L	ttT/ttG
ANKS4B	21261923	T346S	Acg/Tcg
OTOA	21728322	G204V	gGa/gTa
CYLD	50825468	NA	NA
CDH8	61761081	K485Q	Aaa/Caa
AC009113.2	89235032	W116*	tGg/tAg
SPATA22	3352131	NA	NA
SMTNL2	4500584	QKNFE265Q	cagaagaacttcgag/cag
DERL2	5383404	G195A	gGa/gCa
XAF1	6674005	V165D	gTt/gAt
ZBTB4	7366511	A597G	gCt/gGt

DNAH2	7727236	T3766I	aCc/aTc
ALOX15B	7951863	E597Q	Gag/Cag
DHRS7C	9680518	R189H	cGt/cAt
SREBF1	17720648	R129W	Cgg/Tgg
SMCR7	18167263	D184N	Gac/Aac
TBC1D28	18539836	S191Y	tCc/tAc
AKAP10	19835179	P527R	cCt/cGt
GPR179	36485071	E1461Q	Gaa/Caa
NBR1	41327890	N25S	aAt/aGt
FAM171A2	42431578	H668Q	caC/caG
TTLL6	46882163	NA	NA
B4GALNT2	47247085	A480T	Gca/Aca
NXPH3	47656230	I109M	atC/atG
ANKRD40	48777220	D106VN	gac/gTGAac
AC007431.1	55822569	NA	NA
TANC2	61488873	NA	NA
ABCA6	67121109	M396T	aTg/aCg
SDK2	71334947	Y1257H	Tac/Cac
DNAH17	76420162	V4400A	gTc/gCc
DNAH17	76440783	K3797E	Aag/Gag
DNAH17	76565492	D388N	Gac/Aac
SLC26A11	78223024	L532F	Ctc/Ttc
NPTX1	78444762	R146S	Cgc/Agc
L3MBTL4	6171874	P163L	cCg/cTg
DSG3	29052309	G654S	Ggt/Agt
POLI	51804172	Q169L	cAa/cTa
RAX	56939682	E152Q	Gag/Cag
GALR1	74980775	R323C	Cgc/Tgc
CTDP1	77488945	E819A	gAg/gCg
AC006273.1	813267	R487H	cGc/cAc
YIPF2	11036364	Y122C	tAt/tGt
AC024575.1	11470274	HT45P	cacacc/ccc
ZNF844	12187829	T632P	Aca/Cca
CACNA1A	13470490	N303I	aAc/aTc
SLC27A1	17611380	S470R	agC/agA
UNC13A	17716900	R1665L	cGc/cTc
TDRD12	33233788	R141Q	cGa/cAa
KIRREL2	36351915	W295G	Tgg/Ggg
ZNF585A	37642602	L370F	ttG/ttC
WDR87	38378810	E1795G	gAg/gGg
AC008537.4	41442351	T130N	aCc/aAc
AC006486.1	42747336	Q112E	Caa/Gaa
MEGF8	42857202	R1091H	cGc/cAc

LIPE	42907141	P862R	cCc/cGc
RSPH6A	46307590	D525Y	Gac/Tac
SIGLEC6	52023401	K381E	Aag/Gag
ZNF415	53612445	S272P	Tca/Cca
ZNF471	57036667	T411A	Acc/Gcc
ZNF549	58049285	V292M	Gtg/Atg
ZNF416	58084350	S308G	Agc/Ggc
ZIK1	58101495	NA	NA
ZNF814	58385103	E552V	gAg/gTg
ZNF446	58991256	F121L	ttC/ttA
DEFB132	239722	NA	NA
CDC25B	3777312	R45Q	cGg/cAg
KIF16B	16360271	R18S	agG/agT
KIF16B	16362352	L609V	Tta/Gta
MYH7B	33575473	E463Q	Gag/Cag
TSHZ2	51871840	G141R	Ggg/Agg
ZNF217	52198651	T239S	Acc/Tcc
C20orf108	54940257	V101M	Gtg/Atg
C20orf85	56735794	NA	NA
RP4-697K14.7	62194245	R1408Q	cGg/cAg
NRIP1	16338886	P543R	cCc/cGc
APP	27394201	A217AP	gcc/gcGCCc
BACH1	30698501	E119V	gAg/gTg
CLDN8	31588219	A9T	Gct/Act
KRTAP20-1	31988813	G14C	Ggt/Tgt
TIAM1	32493054	E1410*	Gag/Tag
SETD4	37418082	D151G	gAc/gGc
KRTAP12-2	46086706	S33C	tCc/tGc
ARVCF	19960715	D720N	Gac/Aac
SCARF2	20785696	A155S	Gcg/Tcg
INPP5J	31529452	D163N	Gat/Aat
APOL3	36537441	I139N	aTc/aAc
CSF2RB	37325765	V132I	Gta/Ata
CSF2RB	37333993	A662S	Gca/Tca
CACNA1I	40042680	Y419S	tAc/tCc
CACNA1I	40061894	K1294N	aaG/aaT
C22orf34	50014247	W144*	tGg/tAg
TUBGCP6	50656430	P1762L	cCg/cTg
SBF1	50903745	R368C	Cgc/Tgc
DMD	32398732	M1457I	atG/atA
SRPX	38009076	R369H	cGc/cAc
BCOR	39933639	NA	NA

EFHC2	44037689	H38N	Cac/Aac
ZNF157	47272390	E306D	gaG/gaT
CLCN5	49834614	G12W	Ggg/Tgg
CCNB3	50052631	P488A	Cct/Gct
ATP7A	77270162	K726Q	Aaa/Caa
SATL1	84362634	S260R	agC/agG
DACH2	85769382	I197L	Atc/Ctc
ARMCX2	1.01E+08	A164P	Gca/Cca
CXorf41	1.06E+08	S25N	aGt/aAt
AL109749.1	1.31E+08	S223I	aGt/aTt
ZIC3	1.37E+08	HAAA45H	cacgccgccgcc/cac
MCF2	1.39E+08	S201*	tCa/tGa
MAGEC1	1.41E+08	D913G	gAc/gGc
AFF2	1.48E+08	N209I	aAc/aTc
PLXNB3	1.53E+08	V349E	gTg/gAg

Table S3. Variable modifications used for searching MSMS data against the human protein sequence database.

UNIMOD	Description	Monoisotopic mass
1	K-acetylation<br>N-terminal protein acetylation	42.01056469
7	N-deamidation<br>Q-deamidation	0.984015593
21	S-phosphorylation<br>T-phosphorylation	79.966335
36	K-ubiquitinylation (GlyGly)	114.042927
885	Oxidized methionine	15.99491462
43	S-HexNAc<br>T-HexNAc	203.0794
5	Carbamoylation	43.00581367
34	K/R-monomethylation	14.01565
36	K-dimethylation	28.0313
37	K/R-trimethylation	42.04695
385	loss of ammonia	-17.026549
NA	H2O loss	-18.0105647

Figure S1 | Protein sequence coverage obtained by MS on mutated proteins identified by WES.

sp|O14545|TRAD1_HUMAN TRAF−type zinc finger domain−contai
ning protein 1 OS=Homo sapiens GN=TRAFD1 PE=1 SV=1 R 392 G

```
        10        20        30        40        50        60        70        80        90       100
MAEFLDDQETRLCDNCKKEIPVFNFTIHEIHCQRNIGMCPTCKEPFPKSDMETHMAAEHCQVTCKCNKKLEKRLLKKHEETECPLRLAVCQHCDLELSIL
       110       120       130       140       150       160       170       180       190       200
KLKEHEDYCGARTELCGNCGRNVLVKDLKTHPEVCGREGEEKRNEVAIPPNAYDESWGQDGIWIASQLLRQIEALDPPMRLPRRPLRAFESDVFHNRTTN
       210       220       230       240       250       260       270       280       290       300
QRNITAQVSIQNNLFEEQERQERNRGQQPPKEGGEESANLDFMLALSLQNEGQASSVAEQDFWRAVCEADQSHGGPRSLSDIKGAADEIMLPCEFCEELY
       310       320       330       340       350       360       370       380       390       400
PEELLIDHQTSCNPSRALPSLNTGSSSPRGVEEPDVIFQNFLQQAASNQLDSLMGLSNSHPVEESIIIPCEFCGVQLEEEVLFHHQDQCDQGRPATATNH
       410       420       430       440       450       460       470       480       490       500
VTEGIPRLDSQPQETSPELPRRRVRHQGDLSSGYLDDTKQETANGPTSCLPPSRPINNMTATYNQLSRSTSGPRPGCQPSSPCVPKLSNSDSQDIQGRNR
       510       520       530       540       550       560       570
DSQNGAIAPGHVSVIRPPQNLYPENIVPSFSPGPSGRYGASGRSEGGRNSRVTPAAANYRSRTAKAKPSKQQGAGDAEEEEEE
```

sp|O43159|RRP8_HUMAN Ribosomal RNA−processing protein 8 OS=Homo sapiens GN=RRP8 PE=1 SV=2 V 390 A

```
         10        20        30        40        50        60        70        80        90       100
MFEEPEWAEAAPVAAGLGPVISRPPPAASSQNKGSKRRQLLATLRALEAASLSQHPPSLCISDSEEEEEEEKKKCPKKASFASASAEVGKKGKKKCQKQG
        110       120       130       140       150       160       170       180       190       200
PPCSDSEEEVERKKKCHKQALVGSDSAEDEKRKRKCQKHAPINSAQHLDNVDQTGPKAWKGSTTNDPPKQSPGSTSPKPPHTLSRKQWRNRQKNKRRCKN
        210       220       230       240       250       260       270       280       290       300
KFQPPQVPDQAPAEAPTEKTEVSPVPRTDSHEARAGALRARMAQRLDGARFRYLNEQLYSGPSSAAQRLFQEDPEAFLLYHRGFQSQVKKWPLQPVDRIA
        310       320       330       340       350       360       370       380       390       400
RDLRQRPASLVVADFGCGDCRLASSIRNPVHCFDLASLDPRVTVCDMAQVPLEDESVDVAVFCLSLMGTNIRDFLEEANRVLKPGGLLKAVAEVSSRFED
        410       420       430       440
VRTFLRAVTKLGFKIVSKDLTNSHFFLFDFQKTGPPLVGPKAQLSGLQLQPCLYKRR
```

sp|O60244|MED14_HUMAN Mediator of RNA polymerase II transcr
iption subunit 14 OS=Homo sapiens GN=MED14 PE=1 SV=2 T 351 A

```
10 20 30 40 50 60 70 80 90 100
MAPVQLENHQLVPPGGGGGGSGGPPSAPAPPPPGAAVAAAAAAASPGYRLSTLIEFLLHRAYSELMVLTDLLPRKSDVERKIEIVQFASRTRQLFVRLL
110 120 130 140 150 160 170 180 190 200
ALVKWANNAGKVEKCAMISSFLDQQAILFVDTADRLASLARDALVHARLPSFAIPYAIDVLTTGSYPRLPTCIRDKIIPPDPITKIEKQATLHQLNQILR
210 220 230 240 250 260 270 280 290 300
HRLVTTDLPPQLANLTVANGRVKFRVEGEFEATLTVMGDDPDVPWRLLKLEILVEDKETGDGRALVHSMQISFIHQLVQSRLFADEKPLQDMYNCLHSFC
310 320 330 340 350 360 370 380 390 400
LSLQLEVLHSQTLMLIRERWGDLVQVERYHAGKCLSLSVWNQQVLGRKTGATASVHKVTIKIDENDVSKPLQIFHDPPLPASDSKLVERAMKIDHLSIEK
410 420 430 440 450 460 470 480 490 500
LLIDSVHARAHQKLQELKAILRGFNANENSSIETALPALVVPILEPCGNSECLHIFVDLHSGMFQLMLYGLDQATLDDMEKSVNDDMKRIIPWIQQLKFW
510 520 530 540 550 560 570 580 590 600
LGQQRCKQSIKHLPTISSETLQLSNYSTHPIGNLSKNKLFIKLTRLPQYYIVVEMLEVPNKPTQLSYKYYFMSVNAADREDSPAMALLLQQFKENIQDLV
610 620 630 640 650 660 670 680 690 700
FRTKTGKQTRTNAKRKLSDDPCPVESKKTKRAGEMCAFNKVLAHFVAMCDTNMPFVGLRLELSNLEIPHQGVQVEGDGFSHAIRLLKIPPCKGITEETQK
710 720 730 740 750 760 770 780 790 800
ALDRSLLDCTFRLQGRNNRTWVAELVFANCPLNGTSTREQGPSRHVYLTYENLLSEPVGGRKVVEMFLNDWNSIARLYECVLEFARSLPDIPAHLNIFSE
810 820 830 840 850 860 870 880 890 900
VRVYNYRKLILCYGTTKGSSISIQWNSIHQKFHISLGTVGPNSGCSNCHNTILHQLQEMFNKTPNVVQLLQVLFDTQAPLNAINKLPTVPMLGLTQRTNT
910 920 930 940 950 960 970 980 990 1000
AYQCFSILPQSSTHIRLAFRNMYCIDIYCRSRGVVAIRDGAYSLFDNSKLVEGFYPAPGLKTFLNMFVDSNQDARRRSVNEDDNPPSPIGGDMMDSLISQ
1010 1020 1030 1040 1050 1060 1070 1080 1090 1100
LQPPPQQQPFPKQPGTSGAYPLTSPPTSYHSTVNQSPSMMHTQSPGNLHAASSPSGALRAPSPASFVPTPPPSSHGISIGPGASFASPHGTLDPSSPYTM
1110 1120 1130 1140 1150 1160 1170 1180 1190 1200
VSPSGRAGNWPGSPQVSGPSPAARMPGMSPANPSLHSPVPDASHSPRAGTSSQTMPTNMPPPRKLPQRSWAASIPTILTHSALNILLLPSPTPGLVPGLA
1210 1220 1230 1240 1250 1260 1270 1280 1290 1300
GSYLCSPLERFLGSVIMRRHLQRIIQQETLQLINSNEPGVIMFKTDALKCRVALSPKTNQTLQLKVTPENAGQWKPDELQVLEKFFETRVAGPPFKANTL
1310 1320 1330 1340 1350 1360 1370 1380 1390 1400
IAFTKLLGAPTHILRDCVHIMKLELFPDQATQLKWNVQFCLTIPPSAPPIAPPGTPAVVLKSKMLFFLQLTQKTSVPPQEPVSIIVPIIYDMASGTTQQA
1410 1420 1430 1440
DIPRQQNSSVAAPMMVSNILKRFAEMNPPRQGECTIFAAVRDLMANLTLPPGGRP
```

sp|O75306|NDUS2_HUMAN NADH dehydrogenase [ubiquinone] iron−sulfur protein 2, mitochondrial OS=Homo sapiens GN=NDUFS2 PE=1 SV=2 R 35 Q

```
         10        20        30        40        50        60        70        80        90       100
MAALRALCGFRGVAAQVLRPGAGVRLPIQPSRGVRQWQPDVEWAQQFGGAVMYPSKETAHWKPPPWNDVDPPKDTIVKNITLNFGPQHPAAHGVLRLVM
        110       120       130       140       150       160       170       180       190       200
ELSGEMVRKCDPHIGLLHRGTEKLIEYKTYLQALPYFDRLDYVSMMCNEQAYSLAVEKLLNIRPPPRAQWIRVLFGEITRLLNHIMAVTTHALDLGAMTP
        210       220       230       240       250       260       270       280       290       300
FFWLFEEREKMFEFYERVSGARMHAAYIRPGGVHQDLPLGLMDDIYQFSKNFSLRLDELEELLTNNRIWRNRTIDIGVVTAEEALNYGFSGVMLRGSGIQ
        310       320       330       340       350       360       370       380       390       400
WDLRKTQPYDVYDQVEFDVPVGSRGDCYDRYLCRVEEMRQSLRIIAQCLNKMPPGEIKVDDAKVSPPKRAEMKTSMESLIHHFKLYTEGYQVPPGATYTA
        410       420       430       440       450
IEAPKGEFGVYLVSDGSSRPYRCKIKAPGFAHLAGLDKMSKGHMLADVVAIIGTQDIVFGEVDR
```

sp|O95163|ELP1_HUMAN Elongator complex protein 1 OS=Homo sapiens GN=IKBKAP PE=1 SV=3 L 252 F

```
                10        20        30        40        50        60        70        80        90       100
MRNLKLFRTLEFRDIQGPGNPQCFSLRTEQGTVLIGSEHGLIEVDPVSREVKNEVSLVAEGFLPEDGSGRIVGVQDLLDQESVCVATASGDVILCSLSTQ
               110       120       130       140       150       160       170       180       190       200
QLECVGSVASGISVMSWSPDQELVLLATGQQTLIMMTKDFEPILEQQIHQDDFGESKFITVGWGRKETQFHGSEGRQAAFQMQMHESALPWDDHRPQVTW
               210       220       230       240       250       260       270       280       290       300
RGDGQFFAVSVVCPETGARKVRVWNREFALQSTSEPVAGLGPALAWKPSGSFLIASTQDKPNQQDIVFFEKNGLLHGHFTLPFLKDEVKVNDLLWNADSS
               310       320       330       340       350       360       370       380       390       400
VLAVWLEDLQREESSIPKTCVQLWTVGNYHWYLKQSLSFSTCGKSKIVSLMWDPVTPYRLHVLCQGWHYLAYDWHWTTDRSVGDNSSDLSNVAVIDGNRV
               410       420       430       440       450       460       470       480       490       500
LVTVFRQTVVPPPMCTYQLLFPHPVNQVTFLAHPQKSNDLAVLDASNQISVYKCGDCPSADPTVKLGAVGGSGFKVCLRTPHLEKRYKIQFENNEDQDVN
               510       520       530       540       550       560       570       580       590       600
PLKLGLLTWIEEDVFLAVSHSEFSPRSVIHHLTAASSEMDEEHGQLNVSSSAAVDGVIISLCCNSKTKSVVLQLADGQIFKYLWESPSLAIKPWKNSGGF
               610       620       630       640       650       660       670       680       690       700
PVRFPYPCTQTELAMIGEEECVLGLTDRCRFFINDIEVASNITSFAVYDEFLLLTTHSHTCQCFCLRDASFKTLQAGLSSNHVSHGEVLRKVERGSRIVT
               710       720       730       740       750       760       770       780       790       800
VVPQDTKLVLQMPRGNLEVVHHRALVLAQIRKWLDKLMFKEAFECMRKLRINLNLIYDHNPKVFLGNVETFIKQIDSVNHINLFFTELKEEDVTKTMYPA
               810       820       830       840       850       860       870       880       890       900
PVTSSVYLSRDPDGNKIDLVCDAMRAVMESINPHKYCLSILTSHVKKTTPELEIVLQKVHELQGNAPSDPDAVSAEEALKYLLHLVDVNELYDHSLGTYD
               910       920       930       940       950       960       970       980       990      1000
FDLVLMVAEKSQKDPKEYLPFLNTLKKMETNYQRFTIDKYLKRYEKAIGHLSKCGPEYFPECLNLIKDKNLYNEALKLYSPSSQQYQDISIAYGEHLMQE
              1010      1020      1030      1040      1050      1060      1070      1080      1090      1100
HMYEPAGLMFARCGAHEKALSAFLTCGNWKQALCVAAQLNFTKDQLVGLGRTLAGKLVEQRKHIDAAMVLEECAQDYEEAVLLLLEGAAWEEALRLVYKY
              1110      1120      1130      1140      1150      1160      1170      1180      1190      1200
NRLDIIETNVKPSILEAQKNYMAFLDSQTATFSRHKKRLLVVRELKEQAQQAGLDDEVPHGQESDLFSETSSVVSGSEMSGKYSHSNSRISARSSKNRRK
              1210      1220      1230      1240      1250      1260      1270      1280      1290      1300
AERKKHSLKEGSPLEDLALLEALSEVVQNTENLKDEVYHILKVLFLFEFDEQGRELQKAFEDTLQLMERSLPEIWTLTYQQNSATPVLGPNSTANSIMAS
              1310      1320
YQQQKTSVPVLDAELFIPPKINRRTQWKLSLLD
```

sp|P07814|SYEP_HUMAN Bifunctional glutamate/proline−−
tRNA ligase OS=Homo sapiens GN=EPRS PE=1 SV=5 V 1305 L

MATLSLTVNSGDPPLGALLAVEHVKDDVSISVEEGKENILHVSENVIFTDVNSILRYLARVATTAGLYGSNLMEHTEIDHWLEFSATKLSSCDSFTSTIN 100
ELNHCLSLRTYLVGNSLSLADLCVWATLKGNAAWQEQLKQKKAPVHVKRWFGFLEAQQAFQSVGTKWDVSTTKARVAPEKKQDVGKFVELPGAEMGKVTV 200
RFPPEASGYLHIGHAKAALLNQHYQVNFKGKLIMRFDDTNPEKEKEDFEKVILEDVAMLHIKPDQFTYTSDHFETIMKYAEKLIQEGKAYVDDTPAEQMK 300
AEREQRIDSKHRKNPIEKNLQMWEEMKKGSQFGQSCCLRAKIDMSSNNGCMRDPTLYRCKIQPHPRTGNKYNVYPTYDFACPIVDSIEGVTHALRTTEYH 400
DRDEQFYWIIEALGIRKPYIWEYSRLNLNNTVLSKRKLTWFVNEGLVDGWDDPRFPTVRGVLRRGMTVEGLKQFIAAQGSSRSVVNMEWDKIWAFNKKVI 500
DPVAPRYVALLKKEVIPVNVPEAQEEMKEVAKHPKNPEVGLKPVWYSPKVFIEGADAETFSEGEMVTFINWGNLNITKIHKNADGKIISLDAKLNLENKD 600
YKKTTKVTWLAETTHALPIPVICVTYEHLITKPVLGKDEDFKQYVNKNSKHEELMLGDPCLKDLKKGDIIQLQRRGFFICDQPYEPVSPYSCKEAPCVLI 700
YIPDGHTKEMPTSGSKEKTKVEATKNETSAPFKERPTPSLNNNCTTSEDSLVLYNRVAVQGDVVRELKAKKAPKEDVDAAVKQLLSLKAEYKEKTGQEYK 800
PGNPPAEIGQNISSNSSASILESKSLYDEVAAQGEVVRKLKAEKSPKAKINEAVECLLSLKAQYKEKTGKEYIPGQPPLSQSSDSSPTRNSEPAGLETPE 900
AKVLFDKVASQGEVVRKLKTEKAPKDQVDIAVQELLQLKAQYKSLIGVEYKPVSATGAEDKDKKKKEKENKSEKQNKPQKQNDGQRKDPSKNQGGGLSSS 1000
GAGEGQGPKKQTRLGLEAKKEENLADWYSQVITKSEMIEYHDISGCYILRPWAYAIWEAIKDFFDAEIKKLGVENCYFPMFVSQSALEKEKTHVADFAPE 1100
VAWVTRSGKTELAEPIAIRPTSETVMYPAYAKWVQSHRDLPIKLNQWCNVVRWEFKHPQPFLRTREFLWQEGHSAFATMEEAAEEVLQILDLYAQVYEEL 1200
LAIPVVKGRKTEKEKFAGGDYTTTIEAFISASGRAIQGGTSHHLGQNFSKMFEIVFEDPKIPGEKQFAYQNSWGLTTRTIGVMTMVHGDNMGLVLPPRVA 1300
CVQVLVIIPCGITNALSEEDKEALIAKCNDYRRRLLSVNIRVRADLRDNYSPGWKFNHWELKGVPIRLEVGPRDMKSCQFVAVRRDTGEKLTVAENEAET 1400
KLQAILEDIQVTLFTRASEDLKTHMVVANTMEDFQKILDSGKIVQIPFCGEIDCEDWIKKTTARDQDLEPGAPSMGAKSLCIPFKPLCELQPGAKCVCGK 1500
NPAKYYTLFGRSY

sp|P12035|K2C3_HUMAN Keratin, type II cytoskeletal 3 OS=Homo sapiens GN=KRT3 PE=1 SV=3 A 134 P

```
         10        20        30        40        50        60        70        80        90       100
MSRQASKTSGGGSQGFSGRSAVVSGSSRMSCVAHSGGAGGGAYGFRSGAGGFGSRSLYNLGGNKSISISVAAGGSRAGGFGGGRSSCAFAGGYGGGFGSG
        110       120       130       140       150       160       170       180       190       200
YGGGFGGGFGGGRGMGGGFGGAGGFGGAGGFGGPAGGFGGPGGFGGSGGFGGPGSLGSPGGFGPGGFPGGIQEVTINQSLLQPLNVEIDPQIGQVKAQER
        210       220       230       240       250       260       270       280       290       300
EQIKTLNNKFASFIDKVRFLEQQNKVLETKWNLLQQQGTSSISGTNNLEPLFENHINYLRSYLDNILGERGRLDSELKNMEDLVEDFKKKYEDEINKRTA
        310       320       330       340       350       360       370       380       390       400
AENEFVTLKKDVDSAYMNKVELQAKVDALIDEIDFLRTLYDAELSQMQSHISDTSVVLSMDNNRSLDLDSIIAEVRAQYEDIAQRSKAEAEALYQTKLGE
        410       420       430       440       450       460       470       480       490       500
LQTTAGRHGDDLRNTKSEIIELNRMIQRLRAEIEGVKKQNANLQTAIAEAEQHGEMALKDANAKLQELQAALQQAKDDLARLLRDYQELMNVKLALDVEI
        510       520       530       540       550       560       570       580       590       600
ATYRKLLEGEEYRMSGECPSAVSISVVSSSTTSASAGGYGGGYGGGMGGGLGGGFSAGGGSGSGFGRGGGGGIGGGFGGGSSGFSGGSGFGSISGARYGV
        610
SGGGFSSASNRGGSIKFSQSSQSSQRYSR
```

sp|P19338|NUCL_HUMAN Nucleolin OS=Homo sapiens GN=NCL PE=1 SV=3 ED 243 D

```
         10        20        30        40        50        60        70        80        90       100
MVKLAKAGKNQGDPKKMAPPPKEVEEDSEDEEMSEDEEDDSSGEEVVIPQKKGKKAAATSAKKVVVSPTKKVAVATPAKKAAVTPGKKAAATPAKKTVTP
        110       120       130       140       150       160       170       180       190       200
AKAVTTPGKKGATPGKALVATPGKKGAAIPAKGAKNGKNAKKEDSDEEEDDDSEEDEEDDEDEDEDEDEIEPAAMKAAAAAPASEDEDDEDDEDDEDDDD
        210       220       230       240       250       260       270       280       290       300
DEEDDSEEEAMETTPAKGKKAAKVVPVKAKNVAEDEDEEEDDDDEDDDDDEDDEDDDDEDDEEEEEEEEEEPVKEAPGKRKKEMAKQKAAPEAKKQKVEG
        310       320       330       340       350       360       370       380       390       400
TEPTTAFNLFVGNLNFNKSAPELKTGISDVFAKNDLAVVDVRIGMTRKFGYVDFESAEDLEKALELTGLKVFGNEIKLEKPKGKDSKKERDARTLLAKNL
        410       420       430       440       450       460       470       480       490       500
PYKVTQDELKEVFEDAAEIRLVSKDGKSKGIAYIEFKTEADAEKTFEEKQGTEIDGRSISLYYTGEKGQNQDYRGGKNSTWSGESKTLVLSNLSYSATEE
        510       520       530       540       550       560       570       580       590       600
TLQEVFEKATFIKVPQNQNGKSKGYAFIEFASFEDAKEALNSCNKREIEGRAIRLELQGPRGSPNARSQPSKTLFVKGLSEDTTEETLKESFDGSVRARI
        610       620       630       640       650       660       670       680       690       700
VTDRETGSSKGFGFVDFNSEEDAKAAKEAMEDGEIDGNKVTLDWAKPKGEGGFGGRGGGRGGFGGRGGGRGGRGGFGGRGRGGFGGRGGFRGGRGGGGDH

KPQGKKTKFE
```

sp|P24001|IL32_HUMAN Interleukin−32 OS=Homo sapiens GN=IL32 PE=1 SV=3 D 10 N

```
                  10        20        30        40        50        60        70        80        90       100
MCFPKVLSDNDMKKLKARMVMLLPTSAQGLGAWVSACDTEDTVGHLGPWRDKDPALWCQLCLSSQHQAIERFYDKMQNAESGRGQVMSSLAELEDDFKEG
                 110       120       130       140       150       160       170       180       190       200
YLETVAAYYEEQHPELTPLLEKERDGLRCRGNRSPVPDVEDPATEEPGESFCDKVMRWFQAMLQRLQTWWHGVLAWVKEKVVALVHAVQALWKQFQSFCC
                 210       220
SLSELFMSSFQSYGAPRGDKEELTPQKCSEPQSSK
```

sp|P25685|DNJB1_HUMAN DnaJ homolog subfamily B member 1 OS=Homo sapiens GN=DNAJB1 PE=1 SV=4 L 204 F

10 20 30 40 50 60 70 80 90 100
MGKDYYQTLGLARGASDEEIKRAYRRQALRYHPDKNKEPGAEEKFKEIAEAYDVLSDPRKREIFDRYGEEGLKGSGPSGGSGGGANGTSFSYTFHGDPHA

110 120 130 140 150 160 170 180 190 200
MFAEFFGGRNPFDTFFGQRNGEEGMDIDDPFSGFPMGMGGFTNVNFGRSRSAQEPARKKQDPPVTHDLRVSLEEIYSGCTKKMKISHKRLNPDGKSIRNE

210 220 230 240 250 260 270 280 290 300
DKIFLTIEVKKGWKEGTKITFPKEGDQTSNNIPADIVFVLKDKPHNIFKRDGSDVIYPARISLREALCGCTVNVPTLDGRTIPVVFKDVIRPGMRRKVPG

310 320 330
EGLPLPKTPEKRGDLIIEFEVIFPERIPQTSRTVLEQVLPI

sp|P25774|CATS_HUMAN Cathepsin S OS=Homo sapiens GN=CTSS PE=1 SV=3 Q 100 K

```
        10        20        30        40        50        60        70        80        90       100
MKRLVCVLLVCSSAVAQLHKDPTLDHHWHLWKKTYGKQYKEKNEEAVRRLIWEKNLKFVMLHNLEHSMGMHSYDLGMNHLGDMTSEEVMSLMSSLRVPSK
       110       120       130       140       150       160       170       180       190       200
QWQRNITYKSNPNRILPDSVDWREKGCVTEVKYQGSCGACWAFSAVGALEAQLKLKTGKLVSLSAQNLVDCSTEKYGNKGCNGGFMTTAFQYIIDNKGID
       210       220       230       240       250       260       270       280       290       300
SDASYPYKAMDQKCQYDSKYRAATCSKYTELPYGREDVLKEAVANKGPVSVGVDARHPSFFLYRSGVYYEPSCTQNVNHGVLVVGYGDLNGKEYWLVKNS
       310       320
WGHNFGEEGYIRMARNKGNHCGIASFPSYPEI
```

sp|P26640|SYVC_HUMAN Valine−−tRNA ligase OS=Homo sapiens GN=VARS PE=1 SV=4 G 212 R

```
MSTLYVSPHPDAFPSLRALIAARYGEAGEGPGWGGAHPRICLQPPPTSRTPFPPPRLPALEQGPGGLWVWGATAVAQLLWPAGLGGPGGSRAAVLVQQWV 100
SYADTELIPAACGATLPALGLRSSAQDPQAVLGALGRALSPLEEWLRLHTYLAGEAPTLADLAAVTALLLPFRYVLDPPARRIWNNVTRWFVTCVRQPEF 200
RAVLGEVVLYSRGARPLSHQPGPEAPALPKTAAQLKKEAKKREKLEKFQQKQKIQQQQPPPGEKKPKPEKREKRDPGVITYDLPTPPGEKKDVSGPMPDS 300
YSPRYVEAAWYPWWEQQGFFKPEYGRPNVSAANPRGVFMMCIPPPNVTGSLHLGHALTNAIQDSLTRWHRMRGETTLWNPGCDHAGIATQVVVEKKLWRE 400
QGLSRHQLGREAFLQEVWKWKEEKGDRIYHQLKKLGSSLDWDRACFTMDPKLSAAVTEAFVRLHEEGIIYRSTRLVNWSCTLNSAISDIEVDKKELTGRT 500
LLSVPGYKEKVEFGVLVSFAYKVQGSDSDEEVVVATTRIETMLGDVAVAVHPKDTRYQHLKGKNVIHPFLSRSLPIVFDEFVDMDFGTGAVKITPAHDQN 600
DYEVGQRHGLEAISIMDSRGALINVPPPFLGLPRFEARKAVLVALKERGLFRGIEDNPMVVPLCNRSKDVVEPLLRPQWYVRCGEMAQAASAAVTRGDLR 700
ILPEAHQRTWHAWMDNIREWCISRQLWWGHRIPAYFVTVSDPAVPPGEDPDGRYWVSGRNEAEAREKAAKEFGVSPDKISLQQDEDVLDTWFSSGLFPLS 800
ILGWPNQSEDLSVFYPGTLLETGHDILFFWVARMVMLGLKLTGRLPFREVYLHAIVRDAHGRKMSKSLGNVIDPLDVIYGISLQGLHNQLLNSNLDPSEV 900
EKAKEGQKADFPAGIPECGTDALRFGLCAYMSQGRDINLDVNRILGYRHFCNKLWNATKFALRGLGKGFVPSPTSQPGGHESLVDRWIRSRLTEAVRLSN 1000
QGFQAYDFPAVTTAQYSFWLYELCDVYLECLKPVLNGVDQVAAECARQTLYTCLDVGLRLLSPFMPFVTEELFQRLPRRMPQAPPSLCVTPYPEPSECSW 1100
KDPEAEAALELALSITRAVRSLRADYNLTRIRPDCFLEVADEATGALASAVSGYVQALASAGVVAVLALGAPAPQGCAVALASDRCSIHLQLQGLVDPAR 1200
ELGKLQAKRVEAQRQAQRLRERRAASGYPVKVPLEVQEADEAKLQQTEAELRKVDEAIALFQKML
```

sp|P30101|PDIA3_HUMAN Protein disulfide−isomerase A3 OS=Homo sapiens GN=PDIA3 PE=1 SV=4 R 119 S

```
        10        20        30        40        50        60        70        80        90       100
MRLRRLALFPGVALLLAAARLAAASDVLELTDDNFESRISDTGSAGLMLVEFFAPWCGHCKRLAPEYEAAATRLKGIVPLAKVDCTANTNTCNKYGVSGY
       110       120       130       140       150       160       170       180       190       200
PTLKIFRDGEEAGAYDGPRTADGIVSHLKKQAGPASVPLRTEEEFKKFISDKDASIVGFFDDSFSEAHSEFLKAASNLRDNYRFAHTNVESLVNEYDDN
       210       220       230       240       250       260       270       280       290       300
GEGIILFRPSHLTNKFEDKTVAYTEQKMTSGKIKKFIQENIFGICPHMTEDNKDLIQGKDLLIAYYDVDYEKNAKGSNYWRNRVMMVAKKFLDAGHKLNF
       310       320       330       340       350       360       370       380       390       400
AVASRKTFSHELSDFGLESTAGEIPVVAIRTAKGEKFVMQEEFSRDGKALERFLQDYFDGNLKRYLKSEPIPESNDGPVKVVVAENFDEIVNNENKDVLI
       410       420       430       440       450       460       470       480       490
EFYAPWCGHCKNLEPKYKELGEKLSKDPNIVIAKMDATANDVPSPYEVRGFPTIYFSPANKKLNPKKYEGGRELSDFISYLQREATNPPVIQEEKPKKKK
KAQEDL
```

sp|P32248|CCR7_HUMAN C−C chemokine receptor type 7 OS=Homo sapiens GN=CCR7 PE=1 SV=2 V 53 L

```
         10        20        30        40        50        60        70        80        90        100
MDLGKPMKSVLVVALLVIFQVCLCQDEVTDDYIGDNTTVDYTLFESLCSKKDLVRNFKAWFLPIMYSIICFVGLLGNGLVVLTYIYFKRLKTMTDTYLLN
         110       120       130       140       150       160       170       180       190       200
LAVADILFLLTLPFWAYSAAKSWVFGVHFCKLIFAIYKMSFFSGMLLLLCISIDRYVAIVQAVSAHRHRARVLLISKLSCVGIWILATVLSIPELLYSDL
         210       220       230       240       250       260       270       280       290       300
QRSSSEQAMRCSLITEHVEAFITIQVAQMVIGFLVPLLAMSFCYLVIIRTLLQARNFERNKAIKVIIAVVVVFIVFQLPYNGVVLAQTVANFNITSSTCE
         310       320       330       340       350       360
LSKQLNIAYDVTYSLACVRCCVNPFLYAFIGVKFRNDLFKLFKDLGCLSQEQLRQWSSCRHIRRSSMSVEAETTTTFSP
```

sp|P35268|RL22_HUMAN 60S ribosomal protein L22 OS=Homo sapiens GN=RPL22 PE=1 SV=2 KI 69 N

```
         10        20        30        40        50        60        70        80        90       100
MAPVKKLVVKGGKKKKQVLKFTLDCTHPVEDGIMDAANFEQFLQERIKVNGKAGNLGGGVVTIERSKSKITVTSEVPFSKRYLKYLTKKYLKKNNLRDWL
        110
RVVANSKESYELRYFQINQDEEEEEDED
```

sp|P42345|MTOR_HUMAN Serine/threonine−protein kinase mTOR OS=Homo sapiens GN=MTOR PE=1 SV=1 T 2232 I

```
MLGTGPAAATTAATTSSNVSVLQQFASGLKSRNEETRAKAAKELQHYVTMELREMSQEESTRFYDQLNHHIFELVSSSDANERKGGILAIASLIGVEGGN 100
ATRIGRFANYLRNLLPSNDPVVMEMASKAIGRLAMAGDTFTAEYVEFEVKRALEWLGADRNEGRRHAAVLVLRELAISVPTFFFQQVQPFFDNIFVAVWD 200
PKQAIREGAVAALRACLILTTQREPKEMQKPQWYRHTFEEAEKGFDETLAKEKGMNRDDRIHGALLILNELVRISSMEGERLREEMEEITQQQLVHDKYC 300
KDLMGFGTKPRHITPFTSFQAVQPQQSNALVGLLGYSSHQGLMGFGTSPSPAKSTLVESRCCRDLMEEKFDQVCQWVLKCRNSKNSLIQMTILNLLPRLA 400
AFRPSAFTDTQYLQDTMNHVLSCVKKEKERTAAFQALGLLSVAVRSEFKVYLPRVLDIIRAALPPKDFAHKRQKAMQVDATVFTCISMLARAMGPGIQQD 500
IKELLEPMLAVGLSPALTAVLYDLSRQIPQLKKDIQDGLLKMLSLVLMHKPLRHPGMPKGLAHQLASPGLTTLPEASDVGSITLALRTLGSFEFEGHSLT 600
QFVRHCADHFLNSEHKEIRMEAARTCSRLLTPSIHLISGHAHVVSQTAVQVVADVLSKLLVVGITDPDPDIRYCVLASLDERFDAHLAQAENLQALFVAL 700
NDQVFEIRELAICTVGRLSSMNPAFVMPFLRKMLIQILTELEHSGIGRIKEQSARMLGHLVSNAPRLIRPYMEPILKALILKLKDPDPDPNPGVINNVLA 800
TIGELAQVSGLEMRKWVDELFIIIMDMLQDSSLLAKRQVALWTLGQLVASTGYVVEPYRKYPTLLEVLLNFLKTEQNQGTRREAIRVLGLLGALDPYKHK 900
VNIGMIDQSRDASAVSLSESKSSQDSSDYSTSEMLVNMGNLPLDEFYPAVSMVALMRIFRDQSLSHHHTMVVQAITFIFKSLGLKCVQFLPQVMPTFLNV 1000
IRVCDGAIREFLFQQLGMLVSFVKSHIRPYMDEIVTLMREFWVMNTSIQSTIILLIEQIVVALGGEFKLYLPQLIPHMLRVFMHDNSPGRIVSIKLLAAI 1100
QLFGANLDDYLHLLLPPIVKLFDAPEAPLPSRKAALETVDRLTESLDFTDYASRIIHPIVRTLDQSPELRSTAMDTLSSLVFQLGKKYQIFIPMVNKVLV 1200
RHRINHQRYDVLICRIVKGYTLADEEEDPLIYQHRMLRSGQGDALASGPVETGPMKKLHVSTINLQKAWGAARRVSKDDWLEWLRRLSLELLKDSSSPSL 1300
RSCWALAQAYNPMARDLFNAAFVSCWSELNEDQQDELIRSIELALTSQDIAEVTQTLLNLAEFMEHSDKGPLPLRDDNGIVLLGERAAKCRAYAKALHYK 1400
ELEFQKGPTPAILESLISINNKLQQPEAAAGVLEYAMKHFGELEIQATWYEKLHEWEDALVAYDKKMDTNKDDPELMLGRMRCLEALGEWGQLHQQCCEK 1500
WTLVNDETQAKMARMAAAAAWGLGQWDSMEEYTCMIPRDTHDGAFYRAVLALHQDLFSLAQQCIDKARDLLDAELTAMAGESYSRAYGAMVSCHMLSELE 1600
EVIQYKLVPERREIIRQIWWERLQGCQRIVEDWQKILMVRSLVVSPHEDMRTWLKYASLCGKSGRLALAHKTLVLLLGVDPSRQLDHPLPTVHPQVTYAY 1700
MKNMWKSARKIDAFQHMQHFVQTMQQQAQHAIATEDQQHKQELHKLMARCFLKLGEWQLNLQGINESTIPKVLQYYSAATEHDRSWYKAWHAWAVMNFEA 1800
VLHYKHQNQARDEKKKLRHASGANITNATTAATTAATATTTASTEGSNSESEAESTENSPTPSPLQKKVTEDLSKTLLMYTVPAVQGFFRSISLSRGNNL 1900
QDTLRVLTLWFDYGHWPDVNEALVEGVKAIQIDTWLQVIPQLIARIDTPRPLVGRLIHQLLTDIGRYHPQALIYPLTVASKSTTTARHNAANKILKNMCE 2000
HSNTLVQQAMMVSEELIRVAILWHEMWHEGLEEASRLYFGERNVKGMFEVLEPLHAMMERGPQTLKETSFNQAYGRDLMEAQEWCRKYMKSGNVKDLTQA 2100
WDLYYHVFRRISKQLPQLTSLELQYVSPKLLMCRDLELAVPGTYDPNQPIIRIQSIAPSLQVITSKQRPRKLTLMGSNGHEFVFLLKGHEDLRQDERVMQ 2200
LFGLVNTLLANDPTSLRKNLSIQRYAVIPLSITNSGLIGWVPHCDTLHALIRDYREKKKILLNIEHRIMLRMAPDYDHLTLMQKVEVFEHAVNNTAGDDL 2300
AKLLWLKSPSSEVWFDRRTNYTRSLAVMSMVGYILGLGDRHPSNLMLDRLSGKILHIDFGDCFEVAMTREKFPEKIPFRLTRMLTNAMEVTGLDGNYRIT 2400
CHTVMEVLREHKDSVMAVLEAFVYDPLLNWRLMDTNTKGNKRSRTRTDSYSAGQSVEILDGVELGEPAHKKTGTTVPESIHSFIGDGLVKPEALNKKAIQ 2500
IINRVRDKLTGRDFSHDDTLDVPTQVELLIKQATSHENLCQCYIGWCPFW
```

sp|P46013|KI67_HUMAN Antigen KI−67 OS=Homo sapiens GN=MKI67 PE=1 SV=2 K 219 Q

10 20 30 40 50 60 70 80 90 100
MWPTRRLVTIKRSGVDGPHFPLSLSTCLFGRGIECDIRIQLPVVSKQHCKIEIHEQEAILHNFSSTNPTQVNGSVIDEPVRLKHGDVITIIDRSFRYENE
110 120 130 140 150 160 170 180 190 200
SLQNGRKSTEFPRKIREQEPARRVSRSSFSSDPDEKAQDSKAYSKITEGKVSGNPQVHIKNVKEDSTADDSKDSVAQGTTNVHSSEHAGRNGRNAADPIS
210 220 230 240 250 260 270 280 290 300
GDFKEISSVKLVSRYGELQKSVPTTQCLDNSKKNESPFWKLYESVKKELDVKSQKENVLQYCRKSGLQTDYATEKESADGLQGETQLLVSRKSRPKSGGS
310 320 330 340 350 360 370 380 390 400
GHAVAEPASPEQELDQNKGKGRDVESVQTPSKAVGASFPLYEPAKMKTPVQYSQQQNSPQKHKNKDLYTTGRRESVNLGKSEGFKAGDKTLTPRKLSTRN
410 420 430 440 450 460 470 480 490 500
RTPAKVEDAADSATKPENLSSKTRGSIPTDVEVLPTETEIHNEPFLTLWLTQVERKIQKDSLSKPEKLGTTAGQMCSGLPGLSSVDINNFGDSINESEGI
510 520 530 540 550 560 570 580 590 600
PLKRRRVSFGGHLRPELFDENLPPNTPLKRGEAPTKRKSLVMHTPPVLKKIIKEQPQPSGKQESGSEIHVEVKAQSLVISPPAPSPRKTPVASDQRRRSC
610 620 630 640 650 660 670 680 690 700
KTAPASSSKSQTEVPKRGGRKSGNLPSKRVSISRSQHDILQMICSKRRSGASEANLIVAKSWADVVKLGAKQTQTKVIKHGPQRSMNKRQRRPATPKKPV
710 720 730 740 750 760 770 780 790 800
GEVHSQFSTGHANSPCTIIIGKAHTEKVHVPARPYRVLNNFISNQKMDFKEDLSGIAEMFKTPVKEQPQLTSTCHIAISNSENLLGKQFQGTDSGEEPLL
810 820 830 840 850 860 870 880 890 900
PTSESFGGNVFFSAQNAAKQPSDKCSASPPLRRQCIRENGNVAKTPRNTYKMTSLETKTSDTETEPSKTVSTANRSGRSTEFRNIQKLPVESKSEETNTE
910 920 930 940 950 960 970 980 990 1000
IVECILKRGQKATLLQQRREGEMKEIERPFETYKENIELKENDEKMKAMKRSRTWGQKCAPMSDLTDLKSLPDTELMKDTARGQNLLQTQDHAKAPKSEK
1010 1020 1030 1040 1050 1060 1070 1080 1090 1100
GKITKMPCQSLQPEPINTPTHTKQQLKASLGKVGVKEELLAVGKFTRTSGETTHTHREPAGDGKSIRTFKESPKQILDPAARVTGMKKWPRTPKEEAQSL
1110 1120 1130 1140 1150 1160 1170 1180 1190 1200
EDLAGFKELFQTPGPSEESMTDEKTTKIACKSPPPESVDTPTSTKQWPKRSLRKADVEEEFLALRKLTPSAGKAMLTPKPAGGDEKDIKAFMGTPVQKLD
1210 1220 1230 1240 1250 1260 1270 1280 1290 1300
LAGTLPGSKRQLQTPKEKAQALEDLAGFKELFQTPGHTEELVAAGKTTKIPCDSPQSDPVDTPTSTKQRPKRSIRKADVEGELLACRNLMPSAGKAMHTP
1310 1320 1330 1340 1350 1360 1370 1380 1390 1400
KPSVGEEKDIIIFVGTPVQKLDLTENLTGSKRRPQTPKEEAQALEDLTGFKELFQTPGHTEEAVAAGKTTKMPCESSPPESADTPTSTRRQPKTPLEKRD
1410 1420 1430 1440 1450 1460 1470 1480 1490 1500
VQKELSALKKLTQTSGETTHTDKVPGGEDKSINAFRETAKQKLDPAASVTGSKRHPKTKEKAQPLEDLAGLKELFQTPVCTDKPTTHEKTTKIACRSQPD
1510 1520 1530 1540 1550 1560 1570 1580 1590 1600
PVDTPTSSKPQSKRSLRKVDVEEEFFALRKRTPSAGKAMHTPKPAVSGEKNIYAFMGTPVQKLDLTENLTGSKRRLQTPKEKAQALEDLAGFKELFQTRG
1610 1620 1630 1640 1650 1660 1670 1680 1690 1700
HTEESMTNDKTAKVACKSSQPDPDKNPASSKRRLKTSLGKVGVKEELLAVGKLTQTSGETTHTHTEPTGDGKSMKAFMESPKQILDSAASLTGSKRQLRT
1710 1720 1730 1740 1750 1760 1770 1780 1790 1800
PKGKSEVPEDLAGFIELFQTPSHTKESMTNEKTTKVSYRASQPDLVDTPTSSKPQPKRSLRKADTEEEFLAFRKQTPSAGKAMHTPKPAVGEEKDINTFL
1810 1820 1830 1840 1850 1860 1870 1880 1890 1900
GTPVQKLDQPGNLPGSNRRLQTRKEKAQALEELTGFRELFQTPCTDNPTTDEKTTKKILCKSPQSDPADTPTNTKQRPKRSLKKADVEEEFLAFRKLTPS
1910 1920 1930 1940 1950 1960 1970 1980 1990 2000
AGKAMHTPKAAVGEEKDINTFVGTPVEKLDLLGNLPGSKRRPQTPKEKAKALEDLAGFKELFQTPGHTEESMTDDKITEVSCKSPQPDPVKTPTSSKQRL
2010 2020 2030 2040 2050 2060 2070 2080 2090 2100
KISLGKVGVKEEVLPVGKLTQTSGKTTQTHRETAGDGKSIKAFKESAKQMLDPANYGTGMERWPRTPKEEAQSLEDLAGFKELFQTPDHTEESTTDDKTT
2110 2120 2130 2140 2150 2160 2170 2180 2190 2200
KIACKSPPPESMDTPTSTRRRPKTPLGKRDIVEELSALKQLTQTTHTDKVPGDEDKGINVFRETAKQKLDPAASVTGSKRQPRTPKGKAQPLEDLAGLKE
2210 2220 2230 2240 2250 2260 2270 2280 2290 2300
LFQTPICTDKPTTHEKTTKIACRSPQPDPVGTPTIFKPQSKRSLRKADVEEESLALRKRTPSVGKAMDTPKPAGGDEKDMKAFMGTPVQKLDLPGNLPGS
2310 2320 2330 2340 2350 2360 2370 2380 2390 2400
KRWPQTPKEKAQALEDLAGFKELFQTPGTDKPTTDEKTTKIACKSPQPDPVDTPASTKQRPKRNLRKADVEEEFLALRKRTPSAGKAMDTPKPAVSDEKN
2410 2420 2430 2440 2450 2460 2470 2480 2490 2500
INTFVETPVQKLDLLGNLPGSKRQPQTPKEKAEALEDLVGFKELFQTPGHTEESMTDDKITEVSCKSPQPESFKTSRSSKQRLKIPLVKVDMKEEPLAVS
2510 2520 2530 2540 2550 2560 2570 2580 2590 2600
KLTRTSGETTQTHTEPTGDSKSIKAFKESPKQILDPAASVTGSRRQLRTRKEKARALEDLVDFKELFSAPGHTEESMTIDKNTKIPCKSPPPELTDTATS
2610 2620 2630 2640 2650 2660 2670 2680 2690 2700
TKRCPKTRPRKEVKEELSAVERLTQTSGQSTHTHKEPASGDEGIKVLKQRAKKKPNPVEEEPSRRRPRAPKEKAQPLEDLAGFTELSETSGHTQESLTAG
2710 2720 2730 2740 2750 2760 2770 2780 2790 2800
KATKIPCESPPLEVVDTTASTKRHLRTRVQKVQVKEEPSAVKFTQTSGETTDADKEPAGEDKGIKALKESAKQTPAPAASVTGSRRRPRAPRESAQAIED
2810 2820 2830 2840 2850 2860 2870 2880 2890 2900
LAGFKDPAAGHTEESMTDDKTTKIPCKSSPELEDTATSSKRRPRTRAQKVEVKEELLAVGKLTQTSGETTHTDKEPVGEGKGTKAFKQPAKRKLDAEDVI
2910 2920 2930 2940 2950 2960 2970 2980 2990 3000
GSRRQPRAPKEKAQPLEDLASFQELSQTPGHTEELANGAADSFTSAPKQTPDSGKPLKISRRVLRAPKVEPVGDVVSTRDPVKSQSKSNTSLPPLPFKRG
3010 3020 3030 3040 3050 3060 3070 3080 3090 3100
GGKDGSVTGTKRLRCMPAPEEIVEELPASKKQRVAPRARGKSSEPVVIMKRSLRTSAKRIEPAEELNSNDMKTNKEEHKLQDSVPENKGISLRSRRQNKT
3110 3120 3130 3140 3150 3160 3170 3180 3190 3200
EAEQQITEVFVLAERIEINRNEKKPMKTSPEMDIQNPDDGARKPIPRDKVTENKRCLRSARQNESSQPKVAEESGGQKSAKVLMQNQKGKGEAGNSDSMC
3210 3220 3230 3240
LRSRKTKSQPAASTLESKSVQRVTSVKRCAENPKKAEDNVCVKKIRTRSHRDSEDI

sp|P51970|NDUA8_HUMAN NADH dehydrogenase [ubiquinone] 1 alpha subcomplex subunit 8 OS=Homo sapiens GN=NDUFA8 PE=1 SV=3 R 166 C

```
         10        20        30        40        50        60        70        80        90       100
MPGIVELPTLEELKVDEVKISSAVLKAAAHHYGAQCDKPNKEFMLCRWEEKDPRRCLEEGKLVNKCALDFFRQIKRHCAEPFTEYWTCIDYTGQQLFRHC
        110       120       130       140       150       160
RKQQAKFDECVLDKLGWVRPDLGELSKVTKVKTDRPLPENPYHSRPRPDPSPEIEGDLQPATHGSCRFYFWTK
```

sp|P53675|CLH2_HUMAN Clathrin heavy chain 2 OS=Homo sapiens GN=CLTCL1 PE=1 SV=2 G 443 E

10 20 30 40 50 60 70 80 90 100
MAQILPVRFQEHFQLQNLGINPANIGFSTLTMESDKFICIREKVGEQAQVTIIDMSDPMAPIRRPISAESAIMNPASKVIALKAGKTLQIFNIEMKSKMK
110 120 130 140 150 160 170 180 190 200
AHTMAEEVIFWKWVSVNTVALVTETAVYHWSMEGDSQPMKMFDRHTSLVGCQVIHYRTDEYQKWLLLVGISAQQNRVVGAMQLYSVDRKVSQPIEGHAAA
210 220 230 240 250 260 270 280 290 300
FAEFKMEGNAKPATLFCFAVRNPTGGKLHIIEVGQPAAGNQPFVKKAVDVFFPPEAQNDFPVAMQIGAKHGVIYLITKYGYLHLYDLESGVCICMNRISA
310 320 330 340 350 360 370 380 390 400
DTIFVTAPHKPTSGIIGVNKKGQVLSVCVEEDNIVNYATNVLQNPDLGLRLAVRSNLAGAEKLFVRKFNTLFAQGSYAEAAKVAASAPKGILRTRETVQK
410 420 430 440 450 460 470 480 490 500
FQSIPAQSGQASPLLQYFGILLDQGQLNKLESLELCHLVLQQEGRKQLLEKWLKEDKLECSEELGDLVKTTDPMLALSVYLRANVPSKVIQCFAETGQFQ
510 520 530 540 550 560 570 580 590 600
KIVLYAKKVGYTPDWIFLLRGVMKISPEQGLQFSRMLVQDEEPLANISQIVDIFMENSLIQQCTSFLLDALKNNRPAEGLLQTWLLEMNLVHAPQVADAI
610 620 630 640 650 660 670 680 690 700
LGNKMFTHYDRAHIAQLCEKAGLLQQALEHYTDLYDIKRAVVHTHLLNPEWLVNFFGSLSVEDSVECLHAMLSANIRQNLQLCVQVASKYHEQLGTQALV
710 720 730 740 750 760 770 780 790 800
ELFESFKSYKGLFYFLGSIVNFSQDPDVHLKYIQAACKTGQIKEVERICRESSCYNPERVKNFLKEAKLTDQLPLIIVCDRFGFVHDLVLYLYRNNLQRY
810 820 830 840 850 860 870 880 890 900
IEIYVQKVNPSRTPAVIGGLLDVDCSEEVIKHLIMAVRGQFSTDELVAEVEKRNRLKLLLPWLESQIQEGCEEPATHNALAKIYIDSNNSPECFLRENAY
910 920 930 940 950 960 970 980 990 1000
YDSSVVGRYCEKRDPHLACVAYERGQCDLELIKVCNENSLFKSEARYLVCRKDPELWAHVLEETNPSRRQLIDQVVQTALSETRDPEEISVTVKAFMTAD
1010 1020 1030 1040 1050 1060 1070 1080 1090 1100
LPNELIELLEKIVLDNSVFSEHRNLQNLLILTAIKADRTRVMEYISRLDNYDALDIASIAVSSALYEEAFTVFHKFDMNASAIQVLIEHIGNLDRAYEFA
1110 1120 1130 1140 1150 1160 1170 1180 1190 1200
ERCNEPAVWSQLAQAQLQKDLVKEAINSYIRGDDPSSYLEVVQSASRSNNWEDLVKFLQMARKKGRESYIETELIFALAKTSRVSELEDFINGPNNAHIQ
1210 1220 1230 1240 1250 1260 1270 1280 1290 1300
QVGDRCYEEGMYEAAKLLYSNVSNFARLASTLVHLGEYQAAVDNSRKASSTRTWKEVCFACMDGQEFRFAQLCGLHIVIHADELEELMCYYQDRGYFEEL
1310 1320 1330 1340 1350 1360 1370 1380 1390 1400
ILLLEAALGLERAHMGMFTELAILYSKFKPQKMLEHLELFWSRVNIPKVLRAAEQAHLWAELVFLYDKYEEYDNAVLTMMSHPTEAWKEGQFKDIITKVA
1410 1420 1430 1440 1450 1460 1470 1480 1490 1500
NVELCYRALQFYLDYKPLLINDLLLVLSPRLDHTWTVSFFSKAGQLPLVKPYLRSVQSHNNKSVNEALNHLLTEEEDYQGLRASIDAYDNFDNISLAQQL
1510 1520 1530 1540 1550 1560 1570 1580 1590 1600
EKHQLMEFRCIAAYLYKGNNWWAQSVELCKKDHLYKDAMQHAAESRDAELAQKLLQWFLEEGKRECFAACLFTCYDLLRPDMVLELAWRHNLVDLAMPYF
1610 1620 1630
IQVMREYLSKVDKLDALESLRKQEEHVTEPAPLVFDFDGHE

sp|Q13111|CAF1A_HUMAN Chromatin assembly factor 1 s
ubunit A OS=Homo sapiens GN=CHAF1A PE=1 SV=2 R 567 Q

```
                 10        20        30        40        50        60        70        80        90       100
MLEELECGAPGARGAATAMDCKDRPAFPVKKLIQARLPFKRLNLVPKGKADDMSDDQGTSVQSKSPDLEASLDTLENNCHVGSDIDFRPKLVNGKGPLDN
                110       120       130       140       150       160       170       180       190       200
FLRNRIETSIGQSTVIIDLTEDSNEQPDSLVDHNKLNSEASPSREAINGQREDTGDQQGLLKAIQNDKLAFPGETLSDIPCKTEEEGVGCGGAGRRGDSQ
                210       220       230       240       250       260       270       280       290       300
ECSPRSCPELTSGPRMCPRKEQDSWSEAGGILFKGKVPMVVLQDILAVRPPQIKSLPATPQGKNMTPESEVLESFPEEDSVLSHSSLSSPSSTSSPEGPP
                310       320       330       340       350       360       370       380       390       400
APPKQHSSTSPFPTSTPLRRITKKFVKGSTEKNKLRLQRDQERLGKQLKLRAEREEKEKLKEEAKRAKEEAKKKKEEEKELKEKERREKREKDEKEKAEK
                410       420       430       440       450       460       470       480       490       500
QRLKEERRKEREQEALEAKLEEKRKKEEEKRLREEEKRIKAEKAEITRFFQKPKTPQAPKTLAGSCGKFAPFEIKEHMVLAPRRRTAFHPDLCSQLDQLLQ
                510       520       530       540       550       560       570       580       590       600
QQSGEFSFLKDLKGRQPLRSGPTHVSTRNADIFNSDVVIVERGKGDGVPERRKFGRMKLLQFCENHQRPAYWGTWNKKTALIRARDPWAQDTKLLDYEVD
                610       620       630       640       650       660       670       680       690       700
SDEEWEEEEPGESLSHSEGDDDDDMGEDEDEDDGFFVPHGYLSEDEGVTEECADPENHKVRQKLKAKEWDEFLAKGKRFRVLQPVKIGCVWAADRDCAGD
                710       720       730       740       750       760       770       780       790       800
DLKVLQQFAACFLETLPAQEEQTPKASKRERRDEQILAQLLPLLHGNVNGSKVIIREFQEHCRRGLLSNHTGSPRSPSTTYLHTPTPSEDAAIPSKSRLK
                810       820       830       840       850       860       870       880       890       900
RLISENSVYEKRPDFRMCWYVHPQVLQSFQQEHLPVPCQWSYVTSVPSAPKEDSGSVPSTGPSQGTPISLKRKSAGSMCITQFMKKRRHDGQIGAEDMDG
                910       920       930       940
FQADTEEEEEEEGDCMIVDVPDAAEVQAPCGAASGAGGGVGVDTGKATLTSSPLGAS
```

sp|Q14119|VEZF1_HUMAN Vascular endothelial zinc finger 1 OS=Homo sapiens GN=VEZF1 PE=1 SV=2 V 186 A

```
               10        20        30        40        50        60        70        80        90       100
MEANWTAFLFQAHEASHHQQQAAQNSLLPLLSSAVEPPDQKPLLPIPITQKPQGAPETLKDAIGIKKEKPKTSFVCTYCSKAFRDSYHLRRHESCHTGIK
              110       120       130       140       150       160       170       180       190       200
LVSRPKKTPTTVVPLISTIAGDSSRTSLVSTIAGILSTVTTSSSGTNPSSSASTTAMPVTQSVKKPSKPVKKNHACEMCGKAFRDAVYHLNRHKLSHSDE
              210       220       230       240       250       260       270       280       290       300
KPFECPICNQRFKRKDRMTYHVRSHEGGITKPYTCSVCGKGFSRPDHLSCHVKHVHSTERPFKCQTCTAAFATKDRLRTHMVRHEGKVSCNICGKLLSAA
              310       320       330       340       350       360       370       380       390       400
YITSHLKTHGQSQSINCNTCKQGISKTCMSEETSNQKQQQQQQQQQQQQQQQQQHVTSWPGKQVETLRLWEEAVKARKKEAANLCQTSTAATTPVTLTT
              410       420       430       440       450       460       470       480       490       500
PFSITSSVSSGTMSNPVTVAAAMSMRSPVNVSSAVNITSPMNIGHPVTITSPLSMTSPLTLTTPVNLPTPVTAPVNIAHPVTITSPMNLPTPMTLAAPLN
              510
IAMRPVESMPFLPQALPTSPPW
```

sp|Q14584|ZN266_HUMAN Zinc finger protein 266 OS=Homo sapiens GN=ZNF266 PE=2 SV=2 K 247 R

```
         10        20        30        40        50        60        70        80        90       100
MLENYKNLATVGYQLFKPSLISWLEQEESRTVQRGDFQASEWKVQLKTKELALQQDVLGEPTSSGIQMIGSHNGGEVSDVKQCGDVSSEHSCLKTHVRTQ
        110       120       130       140       150       160       170       180       190       200
NSENTFECYLYGVDFLTLHKKTSTGEQRSVFSQCGKAFSLNPDVVCQRTCTGEKAFDCSDSGKSFINHSHLQGHLRTHNGESLHEWKECGRGFIHSTDLA
        210       220       230       240       250       260       270       280       290       300
VRIQTHRSEKPYKCKECGKGFRYSAYLNIHMGTHTGDNPYECKECGRKAFTRSCQLTQHRKTHTGEKPYKCKDCGRAFTVSSCLSQHMKIHVGEKPYECK
        310       320       330       340       350       360       370       380       390       400
ECGIAFTRSSQLTEHLKTHTAKDPFECKICGKSFRNSSCLSDHFRIHTGIKPYKCKDCGKAFTQNSDLTKHARTHSGERPYECKECGKAFARSSRLSEHT
        410       420       430       440       450       460       470       480       490       500
RTHTGEKPFECVKCGKAFAISSNLSGHLRIHTGEKPFECLECGKAFTHSSSLNNHMRTHSAKKPFTCMECGKAFKFPTCVNLHMRIHTGEKPYKCKQCGK
        510       520       530       540
SFSYSNSFQLHERTHTGEKPYECKECGKAFSSSSSFRNHERRHADERLSA
```

sp|Q5VW32|BROX_HUMAN BRO1 domain−containing protein BROX OS=Homo sapiens GN=BROX PE=1 SV=1 Y 249 H

```
        10        20        30        40        50        60        70        80        90       100
MTHWFHRNPLKATAPVSFNYYGVVTGPSASKICNDLRSSRARLLELFTDLSCNPEMMKNAADSYFSLLQGFINSLDESTQESKLRYIQNFKWTDTLQGQV
       110       120       130       140       150       160       170       180       190       200
PSAQQDAVFELISMGFNVALWYTKYASRLAGKENITEDEAKEVHRSLKIAAGIFKHLKESHLPKLITPAEKGRDLESRLIEAYVIQCQAEAQEVTIARAI
       210       220       230       240       250       260       270       280       290       300
ELKHAPGLIAALAYETANFYQKADHTLSSLEPAYSAKWRKYLHLKMCFHYTAYAYCYHGETLLASDKCGEAIRSLQEAEKLYAKAEALCKEYGETKGPGP
       310       320       330       340       350       360       370       380       390       400
TVKPSGHLFFRKLGNLVKNTLEKCQRENGFIYFQKIPTEAPQLELKANYGLVEPIPFEFPPTSVQWTPETLAAFDLTKRPKDDSTKPKPEEEVKPVKEPD
IKPQKDTGCYIS
```

sp|Q6PDB4|ZN880_HUMAN Zinc finger protein 880 OS=Homo sapiens GN=ZNF880 PE=2 SV=2 T 424 N

```
               10        20        30        40        50        60        70        80        90       100
MLRRGHLAFRDVAIEFPQEEWKCLDPAQRTLYREVMVENYRNLVFLGICLPDLSVISMLEQRRDPRNLQSEVKIANNPGGRECIKGVNAESSSKLGSNAG
              110       120       130       140       150       160       170       180       190       200
NKSLKNQLGLTFQLHLSELQLFQAERNISGCKHVEKPINNSLVSPLQKIYSSVKSHILNKYRNDFDDSPFLPQEQKAQIREKPCECNEHGKAFRVSSRLA
              210       220       230       240       250       260       270       280       290       300
NNQVIHTADNPYKCNECDKVFSNSSNLVQHQRIHTGEKPYKCHECGKLFNRISLLARHQRIHTGEKPYKCHECGKVFTQNSHLANHHRIHTGEKPYKCNE
              310       320       330       340       350       360       370       380       390       400
CGKVFNRNAHLARHQKIHSGEKPYKCKECGKAFSGGSGLTAHLVIHTGEKLYKCNKCGKVFNRNAHLTRHQRIHTGEKPYECKECGKVFRHKFCLTNHHR
              410       420       430       440       450       460       470       480       490       500
MHTGEQPYKCNECGKAFRDCSGLNTAHLLIHTGEKPYKCKECAKVFRHRLSLSNHQRFHTGEKPYRCDECGKDFTRNSNLANHHRIHTGEKPYKCSECHK
              510       520       530       540       550       560
VFSHNSHLARHRQIHTGEKSYKCNECGKVFSHKLYLKKHERIHTGEKPYRCHECGKDFTRNSNLANHHRIHTGEKPYR
```

sp|Q71F56|MD13L_HUMAN Mediator of RNA polymerase II transcript
ion subunit 13−like OS=Homo sapiens GN=MED13L PE=1 SV=1 L 699 S

```
         10        20        30        40        50        60        70        80        90       100
MTAAANWVANGASLEDCHSNLFSLAELTGIKWRRYNFGGHGDCGPIISAPAQDDPILLSFIRCLQANLLCVWRRDVKPDCKELWIFWWGDEPNLVGVIHH
        110       120       130       140       150       160       170       180       190       200
ELQVVEEGLWENGLSYECRTLLFKAIHNLLERCLMDKNFVRIGKWFVRPYEKDEKPVNKSEHLSCAFTFFLHGESNVCTSVEIAQHQPIYLINEEHIHMA
        210       220       230       240       250       260       270       280       290       300
QSSPAPFQVLVSPYGLNGTLTGQAYKMSDPATRKLIEEWQYFYPMVLKKKEESKEEDELGYDDDFPVAVEVIVGGVRMVYPSAFVLISQNDIPVPQSVAS
        310       320       330       340       350       360       370       380       390       400
AGGHIAVGQQGLGSVKDPSNCGMPLTPPTSPEQAILGESGGMQSAASHLVSQDGGMITMHSPKRSGKIPPKLHNHMVHRVWKECILNRTQSKRSQMSTPT
        410       420       430       440       450       460       470       480       490       500
LEEEPASNPATWDFVDPTQRVSCSCSRHKLLKRCAVGPNRPPTVSQPGFSAGPSSSSSLPPPASSKHKTAERQEKGDKLQKRPLIPFHHRPSVAEELCME
        510       520       530       540       550       560       570       580       590       600
QDTPGQKLGLAGIDSSLEVSSSRKYDKQMAVPSRNTSKQMNLNPMDSPHSPISPLPPTLSPQPRGQETESLDPPSVPVNPALYGNGLELQQLSTLDDRTV
        610       620       630       640       650       660       670       680       690       700
LVGQRLPLMAEVSETALYCGIRPSNPESSEKWWHSYRLPPSDDAEFRPPELQGERCDAKMEVNSESTALQRLLAQPNKRFKIWQDKQPQLQPLHFLDPSL
        710       720       730       740       750       760       770       780       790       800
PLSQQPGDSLGEVNDPYTFEDGDIKYIFTANKKCKQGTEKDSLKKNKSEDGFGTKDVTTPGHSTPVPDGKNAMSIFSSATKTDVRQDNAAGRAGSSSLTQ
        810       820       830       840       850       860       870       880       890       900
VTDLAPSLHDLDNIFDNSDDDELGAVSPALRSSKMPAVGTEDRPLGKDGRAAVPYPPTVADLQRMFPTPPSLEQHPAFSPVMNYKDGISSETVTALGMME
        910       920       930       940       950       960       970       980       990      1000
SPMVSMVSTQLTEFKMEVEDGLGSPKPEEIKDFSYVHKVPSFQPFVGSSMFAPLKMLPSHCLLPLKIPDACLFRPSWAIPPKIEQLPMPPAATFIRDGYN
       1010      1020      1030      1040      1050      1060      1070      1080      1090      1100
NVPSVGSLADPDYLNTPQMNTPVTLNSAAPASNSGAGVLPSPATPRFSVPTPRTPRTPRTPRGGGTASGQGSVKYDSTDQGSPASTPSTTRPLNSVEPAT
       1110      1120      1130      1140      1150      1160      1170      1180      1190      1200
MQPIPEAHSLYVTLILSDSVMNIFKDRNFDSCCICACNMNIKGADVGLYIPDSSNEDQYRCTCGFSAIMNRKLGYNSGLFLEDELDIFGKNSDIGQAAER
       1210      1220      1230      1240      1250      1260      1270      1280      1290      1300
RLMMCQSTFLPQVEGTKKPQEPPISLLLLLQNQHTQPFASLNFLDYISSNNRQTLPCVSWSYDRVQADNNDYWTECFNALEQGRQYVDNPTGGKVDEALV
       1310      1320      1330      1340      1350      1360      1370      1380      1390      1400
RSATVHSWPHSNVLDISMLSSQDVVRMLLSLQPFLQDAIQKKRTGRTWENIQHVQGPLTWQQFHKMAGRGTYGSEESPEPLPIPTLLVGYDKDFLTISPF
       1410      1420      1430      1440      1450      1460      1470      1480      1490      1500
SLPFWERLLLDPYGGHRDVAYIVVCPENEALLEGAKTFFRDLSAVYEMCRLGQHKPICKVLRDGIMRVGKTVAQKLTDELVSEWFNQPWSGEENDNHSRL
       1510      1520      1530      1540      1550      1560      1570      1580      1590      1600
KLYAQVCRHHLAPYLATLQLDSSLLIPPKYQTPPAAAQGQATPGNAGPLAPNGSAAPPAGSAFNPTSNSSSTNPAASSSASGSSVPPVSSSASAPGISQI
       1610      1620      1630      1640      1650      1660      1670      1680      1690      1700
STTSSSGFSGSVGGQNPSTGGISADRTQGNIGCGGDTDPGQSSSQPSQDGQESVTERERIGIPTEPDSADSHAHPPAVVIYMVDPFTYAAEEDSTSGNFW
       1710      1720      1730      1740      1750      1760      1770      1780      1790      1800
LLSLMRCYTEMLDNLPEHMRNSFILQIVPCQYMLQTMKDEQVFYIQYLKSMAFSVYCQCRRPLPTQIHIKSLTGFGPAASIEMTLKNPERPSPIQLYSPP
       1810      1820      1830      1840      1850      1860      1870      1880      1890      1900
FILAPIKDKQTELGETFGEASQKYNVLFVGYCLSHDQRWLLASCTDLHGELLETCVVNIALPNRSRRSKVSARKIGLQKLWEWCIGIVQMTSLPWRVVIG
       1910      1920      1930      1940      1950      1960      1970      1980      1990      2000
RLGRLGHGELKDWSILLGECSLQTISKKLKDVCRMCGISAADSPSILSACLVAMEPQGSFVVMPDAVTMGSVFGRSTALNMQSSQLNTPQDASCTHILVF
       2010      2020      2030      2040      2050      2060      2070      2080      2090      2100
PTSSTIQVAPANYPNEDGFSPNNDDMFVDLPFPDDMDNDIGILMTGNLHSSPNSSPVPSPGSPSGIGVGSHFQHSRSQGERLLSREAPEELKQQPLALGY
       2110      2120      2130      2140      2150      2160      2170      2180      2190      2200
FVSTAKAENLPQWFWSSCPQAQNQCPLFLKASLHHHISVAQTDELLPARNSQRVPHPLDSKTTSDVLRFVLEQYNALSWLTCNPATQDRTSCLPVHFVVL
TQLYNAIMNIL
```

sp|Q86TU7|SETD3_HUMAN Histone−lysine N−methyltransfe
rase setd3 OS=Homo sapiens GN=SETD3 PE=1 SV=1 Q 572 P

```
         10        20        30        40        50        60        70        80        90       100
MGKKSRVKTQKSGTGATATVSPKEILNLTSELLQKCSSPAPGPGKEWEEYVQIRTLVEKIRKKQKGLSVTFDGKREDYFPDLMKWASENGASVEGFEMVN
        110       120       130       140       150       160       170       180       190       200
FKEEGFGLRATRDIKAEELFLWVPRKLLMTVESAKNSVLGPLYSQDRILQAMGNIALAFHLLCERASPNSFWQPYIQTLPSEYDTPLYFEEDEVRYLQST
        210       220       230       240       250       260       270       280       290       300
QAIHDVFSQYKNTARQYAYFYKVIQTHPHANKLPLKDSFTYEDYRWAVSSVMTRQNQIPTEDGSRVTLALIPLWDMCNHTNGLITTGYNLEDDRCECVAL
        310       320       330       340       350       360       370       380       390       400
QDFRAGEQIYIFYGTRSNAEFVIHSGFFFDNNSHDRVKIKLGVSKSDRLYAMKAEVLARAGIPTSSVFALHFTEPPISAQLLAFLRVFCMTEEELKEHLL
        410       420       430       440       450       460       470       480       490       500
GDSAIDRIFTLGNSEFPVSWDNEVKLWTFLEDRASLLLKTYKTTIEEDKSVLKNHDLSVRAKMAIKLRLGEKEILEKAVKSAAVNREYYRQQMEEKAPLP
        510       520       530       540       550       560       570       580
KYEESNLGLLESSVGDSRLPLVLRNLEEEAGVQDALNIREAISKAKATENGLVNGENSIPNGTRSENESLNPQESKRAVEDAKGSSSDSTAGVKE
```

sp|Q8IZ69|TRM2A_HUMAN tRNA (uracil−5−)−methyltransferas
e homolog A OS=Homo sapiens GN=TRMT2A PE=1 SV=2 G 399 R

```
         10        20        30        40        50        60        70        80        90       100
MSENLDNEGPKPMESCGQESSSALSCPTVSVPPAAPAALEEVEKEGAGAATGPGPQPGLYSYIRDDLFTSEIFKLELQNVPRHASFSDVRRFLGRFGLQP
        110       120       130       140       150       160       170       180       190       200
HKTKLFGQPPCAFVTFRSAAERDKALRVLHGALWKGRPLSVRLARPKADPMARRRRQEGESEPPVTRVADVVTPLWTVPYAEQLERKQLECEQVLQKLAK
        210       220       230       240       250       260       270       280       290       300
EIGSTNRALLPWLLEQRHKHNKACCPLEGVRPSPQQTEYRNKCEFLVGVGVDGEDNTVGCRLGKYKGGTCAVAAPFDTVHIPEATKQVVKAFQEFIRSTP
        310       320       330       340       350       360       370       380       390       400
YSAYDPETYTGHWKQLTVRTSRRHQAMAIAYFHPQKLSPEELAELKTSLAQHFTAGPGRASGVTCLYFVEEGQRKTPSQEGLPLEHVAGDRCIHEDLLRG
        410       420       430       440       450       460       470       480       490       500
LTFRISPHAFFQVNTPAAEVLYTVIQDWAQLDAGSMVLDVCCGTGTIGLALARKVKRVIGVELCPEAVEDARVNAQDNELSNVEFHCGRAEDLVPTLVSR
        510       520       530       540       550       560       570       580       590       600
LASQHLVAILDPPRAGLHSKVILAIRRAKNLRRLLYVSCNPRAAMGNFVDLCRAPSNRVKGIPFRPVKAVAVDLFPQTPHCEMLILFERVEHPNGTGVLG
        610
PHSPPAQPTPGPPDNTLQETGTFPSS
```

sp|Q8NB90|SPAT5_HUMAN Spermatogenesis−associated pr
otein 5 OS=Homo sapiens GN=SPATA5 PE=1 SV=3 Y 324 F

10 20 30 40 50 60 70 80 90 100
MSSKKNRKRLNQSAENGSSLPSAASSCAEARAPSAGSDFAATSGTLTVTNLLEKVDDKIPKTFQNSLIHLGLNTMKSANICIGRPVLLTSLNGKQEVYTA

110 120 130 140 150 160 170 180 190 200
WPMAGFPGGKVGLSEMAQKNVGVRPGDAIQVQPLVGAVLQAEEMDVALSDKDMEINEEELTGCILRKLDGKIVLPGNFLYCTFYGRPYKLQVLRVKGADG

210 220 230 240 250 260 270 280 290 300
MILGGPQSDSDTDAQRMAFEQSSMETSSLELSLQLSQLDLEDTQIPTSRSTPYKPIDDRITNKASDVLLDVTQSPGDGSGLMLEEVTGLKCNFESAREGN

310 320 330 340 350 360 370 380 390 400
EQLTEEERLLKFSIGAKCNTDTFFYFISSTTRVNFTEIDKNSKEQDNQFKVTYDMIGGLSSQLKAIREIIELPLKQPELFKSYGIPAPRGVLLYGPPGTG

410 420 430 440 450 460 470 480 490 500
KTMIARAVANEVGAYVSVINGPEIISKFYGETEAKLRQIFAEATLRHPSIIFIDELDALCPKREGAQNEVEKRVVASLLTLMDGIGSEVSEGQVLVLGAT

510 520 530 540 550 560 570 580 590 600
NRPHALDAALRRPGRFDKEIEIGVPNAQDRLDILQKLLRRVPHLLTEAELLQLANSAHGYVGADLKVLCNEAGLCALRRILKKQPNLPDVKVAGLVKITL

610 620 630 640 650 660 670 680 690 700
KDFLQAMNDIRPSAMREIAIDVPNVSWSDIGGLESIKLKLEQAVEWPLKHPESFIRMGIQPPKGVLLYGPPGCSKTMIAKALANESGLNFLAIKGPELMN

710 720 730 740 750 760 770 780 790 800
KYVGESERAVRETFRKARAVAPSIIFFDELDALAVERGSSLGAGNVADRVLAQLLTEMDGIEQLKDVTILAATNRPDRIDKALMRPGRIDRIIYVPLPDA

810 820 830 840 850 860 870 880
ATRREIFKLQFHSMPVSNEVDLDELILQTDAYSGAEIVAVCREAALLALEEDIQANLIMKRHFTQALSTVTPRIPESLRRFYEDYQEKSGLHTL

sp|Q8ND24|RN214_HUMAN RING finger protein 214 OS=Homo sapiens GN=RNF214 PE=1 SV=2 D 221 G

```
         10        20        30        40        50        60        70        80        90       100
MAASEVAGVVANAPSPPESSSLCASKSDEGLPDGLSTKDSAQKQKNSPLLSVSSQTITKENNRNVHLEHSEQNPGSSAGDTSAAHQVVLGENLIATALCL
        110       120       130       140       150       160       170       180       190       200
SGSGSQSDLKDVASTAGEEGDTSLRESLHPVTRSLKAGCHTKQLASRNCSEEKSPQTSILKEGNRDTSLDFRPVVSPANGVEGVRVDQDDDQDSSSLKLS
        210       220       230       240       250       260       270       280       290       300
QNIAVQTDFKTADSEVNTDQGDIEKNLDKMMTERTLLKERYQEVLDKQRQVENQLQVQLKQLQQRREEEMKNHQEILKAIQDVTIKREETKKKIEKEKKE
        310       320       330       340       350       360       370       380       390       400
FLQKEQDLKAEIEKLCEKGRREVWEMELDRLKNQDGEINRNIMEETERAWKAEILSLESRKELLVLKLEEAEKEAELHLTYLKSTPPTLETVRSKQEWET
        410       420       430       440       450       460       470       480       490       500
RLNGVRIMKKNVRDQFNSHIQLVRNGAKLSSLPQIPTPTLPPPPSETDFMLQVFQPSPSLAPRMPFSIGQVTMPMVMPSADPRSLSFPILNPALSQPSQP
        510       520       530       540       550       560       570       580       590       600
SSPLPGSHGRNSPGLGSLVSPHGPHMPPAASIPPPPGLGGVKASAETPRPQPVDKLEKILEKLLTRFPQCNKAQMTNILQQIKTARTTMAGLTMEELIQL
        610       620       630       640       650       660       670       680       690
VAARLAEHERVAASTQPLGRIRALFPAPLAQISTPMFLPSAQVSYPGRSSHAPATCKLCLMCQKLVQPSELHPMACTHVLHKECIKFWAQTNTNDTCPFC
PTLK
```

sp|Q8NFR7|CC148_HUMAN Coiled−coil domain−containing pr
otein 148 OS=Homo sapiens GN=CCDC148 PE=2 SV=2 D 436 A

```
         10        20        30        40        50        60        70        80        90       100
MCAASASPDNLVFHMKNEMRNIKYKPVDYQQLRALTEAKKLASASAKLKIRKAMLTSKLSKEQTLIKQHKQVWWQEYQRLNEVRCKMESEIKSLLNEENI
        110       120       130       140       150       160       170       180       190       200
GNECLCDLTNFEQELSEQQCTYLKNVINPIQQLRADLKYRQHHTLQHSHPHIEFNSMKVLEEVDFVKKQLKTVFERLRLEQQRIENDLSDWSIKILDHSL
        210       220       230       240       250       260       270       280       290       300
EEKTNPLSELPIELESLECPYPDLKSSILSEFYKFTQKYQKKLQDFNLQLEDIYRNCQLSEEDHWIYQAILDQYPGDLFGRRTLYLDMLQRYFPHKSRHD
        310       320       330       340       350       360       370       380       390       400
LVEHEKYCDQYRFAIEQQNILISNWNKNKKDFIQKAVLTLTEACATHEMESMLAKDKKKQQELCADLKAKVRQWRAHQEEVARLEMEISARRREKEEEKE
        410       420       430       440       450       460       470       480       490       500
KLWKKKELLQRAEKKKKIKKYWAKKKQKWQEMEMRADLQRLEELKKLIAEQSLKDRERVKYRQELLERRLMEKKEVALQEAHEDKERARRLEALRKQVAV
        510       520       530       540       550       560       570       580
VAQFDPVRMMSDTMASKARMGIEIEEEFILQKPLFTLNTYNEQQIISDPRLRFELALREAGLHRTLYAKEILPKISPQKPPRKDMESTVFKI
```

sp|Q8NHQ1|CEP70_HUMAN Centrosomal protein of 70 kDa OS=Homo sapiens GN=CEP70 PE=1 SV=2 K 162 I

10 20 30 40 50 60 70 80 90 100
MFPVAPKPQDSSQPSDRLMTEKQQEEAEWESINVLLMMHGLKPLSLVKRTDLKDLIIFDKQSSQRMRQNLKLLVEETSCQQNMIQELIETNQQLRNELQL
110 120 130 140 150 160 170 180 190 200
EQSRAANQEQRANDLEQIMESVKSKIGELEDESLSRACHQQNKIKDLQKEQKTLQVKCQHYIKKKRTEQEETIASLQMEVCRLKKEEEDRIVTQNRVFAY
210 220 230 240 250 260 270 280 290 300
LCKRVPHTVLDRQLLCLIDYYESKIRKIHTQRQYKEDESQSEEENDYRNLDASPTYKGLLMSLQNQLKESKSKIDALSSEKLNLQKDLETRPTQHELRLY
310 320 330 340 350 360 370 380 390 400
KQQVKKLEKALKKNVKLQELINHKKAEDTEKKDEPSKYNQQQALIDQRYFQVLCSINSIIHNPRAPVIIYKQTKGGVQNFNKDLVQDCGFEHLVPVIEMW
410 420 430 440 450 460 470 480 490 500
ADQLTSLKDLYKSLKTLSAELVPWLNLKKQDENEGIKVEDLLFIVDTMLEEVENKEKDSNMPHFQTLQAIVSHFQKLFDVPSLNGVYPRMNEVYTRLGEM
510 520 530 540 550 560 570 580
NNAVRNLQELLELDSSSSLCVLVSTVGKLCRLINEDVNEQVMQVLGPEDLQSIIYKLEEHEEFFPAFQAFTNDLLEILEIDDLDAIVPAVKKLKVLSY

sp|Q96H55|MYO19_HUMAN Unconventional myosin−XIX OS=Homo sapiens GN=MYO19 PE=2 SV=2 K 118 R

```
         10        20        30        40        50        60        70        80        90       100
MLQQVNGHNPGSDGQAREYLREDLQEFLGGEVLLYKLDDLTRVNPVTLETVLRCLQARYMADTFYTNAGCTLVALNPFKPVPQLYSPELMREYHAAPQPQ
        110       120       130       140       150       160       170       180       190       200
KLKPHVFTVGEQTYRNVRKSLIEPVNQSIVVSGESGAGKTWTSRCLMKFYAVVATSPASWESHKIAERIEQRILNSNPVMEAFGNACTLRNNNSSRFGKF
        210       220       230       240       250       260       270       280       290       300
IQLQLNRAQQMTGAAVQTYLLEKTRVACQASSERNFHIFYQICKGASEDERLQWHLPEGAAFSWLPNPERSLEEDCFEVTREAMLHLGIDTPTQNNIFKV
        310       320       330       340       350       360       370       380       390       400
LAGLLHLGNIQFAASEDEAQPCQPMDDAKYSVRTAASLLGLPEDVLLEMVQIRTIRAGRQQQVFRKPCARAECDTRRDCLAKLIYARLFDWLVSVINSSI
        410       420       430       440       450       460       470       480       490       500
CADTDSWTTFIGLLDVYGFESFPDNSLEQLCINYANEKLQQHFVAHYLRAQQEEYAVEGLEWSFINYQDNQPCLDLIEGSPISICSLINEECRLNRPSSA
        510       520       530       540       550       560       570       580       590       600
AQLQTRIETALAGSPCLGHNKLSREPSFIVVHYAGPVRYHTAGLVEKNKDPIPPELTRLLQQSQDPLLMGLFPTNPKEKTQEEPPGQSRAPVLTVVSKFK
        610       620       630       640       650       660       670       680       690       700
ASLEQLLQVLHSTTPHYIRCIKPNSQGQAQTFLQEEVLSQLEACGLVETIHISAAGFPIRVSHRNFVERYKLLRRLHPCTSSGPDSPYPAKGLPEWCPHS
        710       720       730       740       750       760       770       780       790       800
EEATLEPLIQDILHTLPVLTQAAAITGDSAEAMPAPMHCGRTKVFMTDSMLELLECGRARVLEQCARCIQGGWRRHRHREQERQWRAVMLIQAAIRSWLT
        810       820       830       840       850       860       870       880       890       900
RKHIQRLHAAATVIKRAWQKWRIRMACLAAKELDGVEEKHFSQAPCSLSTSPLQTRLLEAIIRLWPLGLVLANTAMGVGSFQRKLVVWACLQLPRGSPSS
        910       920       930       940       950       960
YTVQTAQDQAGVTSIRALPQGSIKFHCRKSPLRYADICPEPSPYSITGFNQILLERHRLIHVTSSAFTGLG
```

sp|Q96LR5|UB2E2_HUMAN Ubiquitin−conjugating enzyme E2 E2 OS=Homo sapiens GN=UBE2E2 PE=1 SV=1 T 14 A

```
         10        20        30        40        50        60        70        80        90       100
MSTEAQRVDDSPSATSGGSSDGDQRESVQQEPEREQVQPKKKEGKISSKTAAKLSTSAKRIQKELAEITLDPPPNCSAGPKGDNIYEWRSTILGPPGSVY
        110       120       130       140       150       160       170       180       190
EGGVFFLDITFSPDYPFKPPKVTFRTRIYHCNINSQGVICLDILKDNWSPALTISKVLLSICSLLTDCNPADPLVGSIATQYMTNRAEHDRMARQWTKRY
AT
```

sp|Q96SZ5|AEDO_HUMAN 2−aminoethanethiol dioxygenase OS=Homo sapiens GN=ADO PE=1 SV=2 Q 158 H

```
         10        20        30        40        50        60        70        80        90       100
MPRDNMASLIQRIARQACLTFRGSGGGRGASDRDAASGPEAPMQPGFPENLSKLKSLLTQLRAEDLNIAPRKATLQPLPPNLPPVTYMHIYETDGFSLGV
        110       120       130       140       150       160       170       180       190       200
FLLKSGTSIPLHDHPGMHGMLKVLYGTVRISCMDKLDAGGGQRPRALPPEQQFEPPLHQPREREAVRPGVLRSRAEYTEASGPCILTPHRDNLHQIDAVE
        210       220       230       240       250       260
GPAAFLDILAPPYDPDDGRDCHYYRVLEPVRPKEASSSACDLPREVWLLETPQADDFWCEGEPYPGPKVFP
```

sp|Q9BQ39|DDX50_HUMAN ATP−dependent RNA helicase DDX50 OS=Homo sapiens GN=DDX50 PE=1 SV=1 R 689 Q

```
         10        20        30        40        50        60        70        80        90       100
MPGKLLWGDIMELEAPLEESESQKKERQKSDRRKSRHHYDSDEKSETRENGVTDDLDAPKAKKSKMKEKLNGDTEEGFNRLSDEFSKSHKSRRKDLPNGD
        110       120       130       140       150       160       170       180       190       200
IDEYEKKSKRVSSLDTSTHKSSDNKLEETLTREQKEGAFSNFPISEETIKLLKGRGVTYLFPIQVKTFGPVYEGKDLIAQARTGTGKTFSFAIPLIERLQ
        210       220       230       240       250       260       270       280       290       300
RNQETIKKSRSPKVLVLAPTRELANQVAKDFKDITRKLSVACFYGGTSYQSQINHIRNGIDILVGTPGRIKDHLQSGRLDLSKLRHVVLDEVDQMLDLGF
        310       320       330       340       350       360       370       380       390       400
AEQVEDIIHESYKTDSEDNPQTLLFSATCPQWVYKVAKKYMKSRYEQVDLVGKMTQKAATTVEHLAIQCHWSQRPAVIGDVLQVYSGSEGRAIIFCETKK
        410       420       430       440       450       460       470       480       490       500
NVTEMAMNPHIKQNAQCLHGDIAQSQREITLKGFREGSFKVLVATNVAARGLDIPEVDLVIQSSPPQDVESYIHRSGRTGRAGRTGICICFYQPRERGQL
        510       520       530       540       550       560       570       580       590       600
RYVEQKAGITFKRVGVPSTMDLVKSKSMDAIRSLASVSYAAVDFFRPSAQRLIEEKGAVDALAAALAHISGASSFEPRSLITSDKGFVTMTLESLEEIQD
        610       620       630       640       650       660       670       680       690       700
VSCAWKELNRKLSSNAVSQITRMCLLKGNMGVCFDVPTTESERLQAEWHDSDWILSVPAKLPEIEEYYDGNTSSNSRQRSGWSSGRSGQRSGRSGGRSGG
        710       720
RSGRQSRQGSRSGSRQDGRRRSGNRNRSRSGGHKRSFD
```

sp|Q9H0C8|ILKAP_HUMAN Integrin−linked kinase−associated serine/t
hreonine phosphatase 2C OS=Homo sapiens GN=ILKAP PE=1 SV=1 A 54 T

```
        10        20        30        40        50        60        70        80        90       100
MDLFGDLPEPERSPRPAAGKEAQKGPLLFDDLPPASSTDSGSGGPLLFDDLPPTASSGDSGSLATSISQMVKTEGKGAKRKTSEEEKNGSEELVEKKVCK
       110       120       130       140       150       160       170       180       190       200
ASSVIFGLKGYVAERKGEREEMQDAHVILNDITEECRPPSSLITRVSYFAVFDGHGGIRASKFAAQNLHQNLIRKFPKGDVISVEKTVKRCLLDTFKHTD
       210       220       230       240       250       260       270       280       290       300
EEFLKQASSQKPAWKDGSTATCVLAVDNILYIANLGDSRAILCRYNEESQKHAALSLSKEHNPTQYEERMRIQKAGGNVRDGRVLGVLEVSRSIGDGQYK
       310       320       330       340       350       360       370       380
RCGVTSVPDIRRCQLTPNDRFILLACDGLFKVFTPEEAVNFILSCLEDEKIQTREGKSAADARYEAACNRLANKAVQRGSADNVTVMVVRIGH
```

sp|Q9HB90|RRAGC_HUMAN Ras−related GTP−binding protein C OS=Homo sapiens GN=RRAGC PE=1 SV=1 T 96 A

```
          10        20        30        40        50        60        70        80        90       100
MSLQYGAEETPLAGSYGAADSFPKDFGYGVEEEEEEAAAAGGGVGAGAGGGCGPGGADSSKPRILLMGLRRSGKSSIQKVVFHKMSPNETLFLESATNKI
         110       120       130       140       150       160       170       180       190       200
YKDDISNSSFVNFQIWDFPGQMDFFDPTFDYEMIFRGTGALIYVIDAQDDYMEALTRLHITVSKAYKVNPDMNFEVFIHKVDGLSDDHKIETQRDIHQRA
         210       220       230       240       250       260       270       280       290       300
NDDLADAGLEKLHLSFYLTSIYDHSIFEAFSKVVQKLIPQLPTLENLLNIFISNSGIEKAFLFDVVSKIYIATDSSPVDMQSYELCCDMIDVVIDVSCIY
         310       320       330       340       350       360       370       380       390
GLKEDGSGSAYDKESMAIIKLNNTTVLYLKEVTKFLALVCILREESFERKGLIDYNFHCFRKAIHEVFEVGVTSHRSCGHQTSASSLKALTHNGTPRNAI
```

sp|Q9NW82|WDR70_HUMAN WD repeat−containing protein 70 OS=Homo sapiens GN=WDR70 PE=1 SV=1 S 450 R

```
          10        20        30        40        50        60        70        80        90       100
MERSGPSEVTGSDASGPDPQLAVTMGFTGFGKKARTFDLEAMFEQTRRTAVERSRKTLEAREKEEEMNREKELRRQNEDIEPTSSRSNVVRDCSKSSSRD
         110       120       130       140       150       160       170       180       190       200
TSSSESEQSSDSSDDELIGPPLPPKMVGKPVNFMEEDILGPLPPPLNEEEEEAEEEEEEEEEEENPVHKIPDSHEITLKHGTKTVSALGLDPSGARLVTG
         210       220       230       240       250       260       270       280       290       300
GYDYDVKFWDFAGMDASFKAFRSLQPCECHQIKSLQYSNTGDMILVVSGSSQAKVIDRDGFEVMECIKGDQYIVDMANTKGHTAMLHTGSWHPKIKGEFM
         310       320       330       340       350       360       370       380       390       400
TCSNDATVRTWEVENPKKQKSVFKPRTMQGKKVIPTTCTYSRDGNLIAAACQNGSIQIWDRNLTVHPKFHYKQAHDSGTDTSCVTFSYDGNVLASRGGDD
         410       420       430       440       450       460       470       480       490       500
SLKLWDIRQFNKPLFSASGLPTMFPMTDCCFSPDDKLIVTGTSIQRGCGRSGKLVFFERRTFQRVYEIDITDASVVRCLWHPKLNQIMVGTGNGLAKVYY
         510       520       530       540       550       560       570       580       590       600
DPNKSQRGAKLCVVKTQRKAKQAETLTQDYIITPHALPMFREPRQRSTRKQLEKDRLDPLKSHKPEPPVAGPGRGGRVGTHGGTLSSYIVKNIALDKTDD
         610       620       630       640
SNPREAILRHAKAAEDSPYWVSPAYSKTQPKTMFAQVESDDEEAKNEPEWKKRKI
```

sp|Q9NWQ8|PHAG1_HUMAN Phosphoprotein associated with glycosphingoli
pid−enriched microdomains 1 OS=Homo sapiens GN=PAG1 PE=1 SV=2 S 67 N

```
                 10        20        30        40        50        60        70        80        90       100
MGPAGSLLGSGQMQITLWGSLAAVAIFFVITFLIFLCSSCDREKKPRQHSGDHENLMNVPSDKEMFNSRSVTSLATDAPASSEQNGALTNGDILSEDSTL
                110       120       130       140       150       160       170       180       190       200
TCMQHYEEVQTSASDLLDSQDSTGKPKCHQSRELPRIPPESAVDTMLTARSVDGDQGLGMEGPYEVLKDSSSQENMVEDCLYETVKEIKEVAAAAHLEKG
                210       220       230       240       250       260       270       280       290       300
HSGKAKSTSASKELPGPQTEGKAEFAEYASVDRNKKCRQSVNVESILGNSCDPEEEAPPPVPVKLLDENENLQEKEGGEAEESATDTTSETNKRFSSLSY
                310       320       330       340       350       360       370       380       390       400
KSREEDPTLTEEEISAMYSSVNKPGQLVNKSGQSLTVPESTYTSIQGDPQRSPSSCNDLYATVKDFEKTPNSTLPPAGRPSEEPEPDYEAIQTLNREEEK
                410       420
ATLGTNGHHGLVPKENDYESISDLQQGRDITRL
```

sp|Q9UJU2|LEF1_HUMAN Lymphoid enhancer−binding factor 1 OS=Homo sapiens GN=LEF1 PE=1 SV=1 P 44 A

```
                 10        20        30        40        50        60        70        80        90       100
MPQLSGGGGGGGGDPELCATDEMIPFKDEGDPQKEKIFAEISHAPEEEGDLADIKSSLVNESEIIPASNGHEVARQAQTSQEPYHDKAREHPDDGKHPDG
                110       120       130       140       150       160       170       180       190       200
GLYNKGPSYSSYSGYIMMPNMNNDPYMSNGSLSPPIPRTSNKVPVVQPSHAVHPLTPLITYSDEHFSPGSHPSHIPSDVNSKQGMSRHPPAPDIPTFYPL
                210       220       230       240       250       260       270       280       290       300
SPGGVGQITPPLGWQGQPVYPITGGFRQPYPSSLSVDTSMSRFSHHMIPGPPGPHTTGIPHPAIVTPQVKQEHPHTDSDLMHVKPQHEQRKEQEPKRPHI
                310       320       330       340       350       360       370       380       390
KKPLNAFMLYMKEMRANVVAECTLKESAAINQILGRRWHALSREEQAKYYELARKERQLHMQLYPGWSARDNYGKKKKRKREKLQESASGTGPRMTAAYI
```

sp|Q9UQ88|CD11A_HUMAN Cyclin−dependent kinase 11A OS=Homo sapiens GN=CDK11A PE=1 SV=4 − 117 KK

10 20 30 40 50 60 70 80 90 100
MGDEKDSWKVKTLDEILQEKKRRKEQEEKAEIKRLKNSDDRDSKRDSLEEGELRDHCMEITIRNSPYRREDSMEDRGEEDDSLAIKPPQQMSRKEKVHHR

110 120 130 140 150 160 170 180 190 200
KDEKRKEKWKHARVKEKKREHERRKRHREEQDKARREWERQKRREMAREHSRRERDRLEQLERKRERERKMREQQKEQREQKERERRAEERRKEREARRE

210 220 230 240 250 260 270 280 290 300
VSAHHRTMREDYSDKVKASHWSRSPPRPPRERFELGDGRKPGEARPAPAQKPAQLKEEKMEERDLLSDLQDISDSERKTSSAESSSAESGSGSEEEEEEE

310 320 330 340 350 360 370 380 390 400
EEEEEEGSTSEESEEEEEEEEEEEEETGSNSEEASEQSAEEVSEEEMSEDEERENENHLLVVPESRFDRDSGESEEAEEEVGEGTPQSSALTEGDYVPDS

410 420 430 440 450 460 470 480 490 500
PALLPIELKQELPKYLPALQGCRSVEEFQCLNRIEEGTYGVVYRAKDKKTDEIVALKRLKMEKEKEGFPITSLREINTILKAQHPNIVTVREIVVGSNMD

510 520 530 540 550 560 570 580 590 600
KIYIVMNYVEHDLKSLMETMKQPFLPGEVKTLMIQLLRGVKHLHDNWILHRDLKTSNLLLSHAGILKVGDFGLAREYGSPLKAYTPVVVTQWYRAPELLL

610 620 630 640 650 660 670 680 690 700
GAKEYSTAVDMWSVGCIFGELLTQKPLFPGNSEIDQINKVFKELGTPSEKIWPGYSELPVVKKMTFSEHPYNNLRKRFGALLSDQGFDLMNKFLTYFPGR

710 720 730 740 750 760 770
RISAEDGLKHEYFRETPLPIDPSMFPTWPAKSEQQRVKRGTSPRPPEGGLGYSQLGDDDLKETGFHLTTTNQGASAAGPGFSLKF

sp|Q9Y2Q9|RT28_HUMAN 28S ribosomal protein S28, mito
chondrial OS=Homo sapiens GN=MRPS28 PE=1 SV=1 L 62 F

```
                10        20        30        40        50        60        70        80        90       100
MAALCRTRAVAAESHFLRVFLFFRPFRGVGTESGSESGSSNAKEPKTRAGGFASALERHSEFLLQKVEPLQKGSPKNVESFASMLRHSPLTQMGPAKDKL
               110       120       130       140       150       160       170
VIGRIFHIVENDLYIDFGGKFHCVCRRPEVDGEKYQKGTRVRLRLLDLELTSRFLGATTDTTVLEANAVLLGIQESKDSRSKEEHHEK
```

sp|Q9Y5B9|SP16H_HUMAN FACT complex subunit SPT16 OS=Homo sapiens GN=SUPT16H PE=1 SV=1 Q 336 L

10 20 30 40 50 60 70 80 90 100
MAVTLDKDAYYRRVKRLYSNWRKGEDEYANVDAIVVSVGVDEEIVYAKSTALQTWLFGYELTDTIMVFCDDKIIFMASKKKVEFLKQIANTKGNENANGA
110 120 130 140 150 160 170 180 190 200
PAITLLIREKNESNKSSFDKMIEAIKESKNGKKIGVFSKDKFPGEFMKSWNDCLNKEGFDKIDISAVVAYTIAVKEDGELNLMKKAASITSEVFNKFFKE
210 220 230 240 250 260 270 280 290 300
RVMEIVDADEKVRHSKLAESVEKAIEEKKYLAGADPSTVEMCYPPIIQSGGNYNLKFSVVSDKNHMHFGAITCAMGIRFKSYCSNLVRTLMVDPSQEVQE
310 320 330 340 350 360 370 380 390 400
NYNFLLQLQEELLKELRHGVKICDVYNAVMDVVKKLQKPELLNKITKNLGFGMGIEFREGSLVINSKNQYKLKKGMVFSINLGFSDLTNKEGKKPEEKTY
410 420 430 440 450 460 470 480 490 500
ALFIGDTVLVDEDGPATVLTSVKKKVKNVGIFLKNEDEEEEEEEKDEAEDLLGRGSRAALLTERTRNEMTAEEKRRAHQKELAAQLNEEAKRRLTEQKGE
510 520 530 540 550 560 570 580 590 600
QQIQKARKSNVSYKNPSLMPKEPHIREMKIYIDKKYETVIMPVFGIATPFHIATIKNISMSVEGDYTYLRINFYCPGSALGRNEGNIFPNPEATFVKEIT
610 620 630 640 650 660 670 680 690 700
YRASNIKAPGEQTVPALNLQNAFRIIKEVQKRYKTREAEEKEKEGIVKQDSLVINLNRSNPKLKDLYIRPNIAQKRMQGSLEAHVNGFRFTSVRGDKVDI
710 720 730 740 750 760 770 780 790 800
LYNNIKHALFQPCDGEMIIVLHFHLKNAIMFGKKRHTDVQFYTEVGEITTDLGKHQHMHDRDDLYAEQMEREMRHKLKTAFKNFIEKVEALTKEELEFEV
810 820 830 840 850 860 870 880 890 900
PFRDLGFNGAPYRSTCLLQPTSSALVNATEWPPFVVTLDEVELIHFERVQFHLKNFDMVIVYKDYSKKVTMINAIPVASLDPIKEWLNSCDLKYTEGVQS
910 920 930 940 950 960 970 980 990 1000
LNWTKIMKTIVDDPEGFFEQGGWSFLEPEGEGSDAEEGDSESEIEDETFNPSEDDYEEEEEDSDEDYSSEAEESDYSKESLGSEEESGKDWDELEEEARK
1010 1020 1030
ADRESRYEEEEEQSRSMSRKRKASVHSSGRGSNRGSRHSSAPPKKKRK

tr|A0A087WU55|A0A087WU55_HUMAN Unconventional myosin−XI
X (Fragment) OS=Homo sapiens GN=MYO19 PE=4 SV=2 K 118 R

```
                10        20        30        40        50        60        70        80        90       100
MLQQVNGHNPGSDGQAREYLREDLQEFLGGEVLLYKLDDLTRVNPVTLETVLRCLQARYMADTFYTNAGCTLVALNPFKPVPQLYSPELMREYHAAPQPQ
               110       120       130       140       150       160
KLKPHVFTVGEQTYRNVRKSLIEPVNQSIVVSGESGAGKTWTSRCLMKFYAVVATSPASWESHKIAERIEQRILNSN
```

tr|A0A087WV66|A0A087WV66_HUMAN Antigen KI−67 OS=Homo sapiens GN=MKI67 PE=1 SV=1 K 219 Q

MWPTRRLVTIKRSGVDGPHFPLSLSTCLFGRGIECDIRIQLPVVSKQHCKIEIHEQEAILHNFSSTNPTQVNGSVIDEPVRLKHGDVITIIDRSFRYENE 100
SLQNGRKSTEFPRKIREQEPARRVSRSSFSSDPDEKAQDSKAYSKITEGKVSGNPQVHIKNVKEDSTADDSKDSVAQGTTNVHSSEHAGRNGRNAADPIS 200
GDFKEISSVKLVSRYGELQKSVPTTQCLDNSKKNESPFWKLYESVKKELDVKSQKENVLQYCRKSGLQTDYATEKESADGLQGETQLLVSRKSRPKSGGS 300
GHAVAEPASPEQELDQNKGKGRDVESVQTPSKAVGASFPLYEPAKMKTPVQYSQQQNSPQKHKNKDLYTTGRRESVNLGKSEGFKAGDKTLTPRKLSTRN 400
RTPAKVEDAADSATKPENLSSKTRGSIPTDVEVLPTETEIHNEPFLTLWLTQVERKIQKDSLSKPEKLGTTAGQMCSGLPGLSSVDINNFGDSINESEGI 500
PLKRRRVSFGGHLRPELFDENLPPNTPLKRGEAPTKRKSLVMHTPPVLKKIIKEQPQPSGKQESGSEIHVEVKAQSLVISPPAPSPRKTPVASDQRRRSC 600
KTAPASSSKSQTEVPKRGGERVATCLQRVSISRSQHDILQMICSKRRSGASEANLIVAKSWADVVKLGAKQTQTKVIKHGPQRSMNKRQRRPATPKKPVG 700
EVHSQFSTGHANSPCTIIIGKAHTEKVHVPARPYRVLNNFISNQKMDFKEDLSGIAEMFKTPVKEQPQLTSTCHIAISNSENLLGKQFQGTDSGEEPLLP 800
TSESFGGNVFFSAQNAAKQPSDKCSASPPLRRQCIRENGNVAKTPRNTYKMTSLETKTSDTETEPSKTVSTANRSGRSTEFRNIQKLPVESKSEETNTEI 900
VECILKRGQKATLLQQRREGEMKEIERPFETYKENIELKENDEKMKAMKRSRTWGQKCAPMSDLTDLKSLPDTELMKDTARGQNLLQTQDHAKAPKSEKG 1000
KITKMPCQSLQPEPINTPTHTKQQLKASLGKVGVKEELLAVGKFTRTSGETTHTHREPAGDGKSIRTFKESPKQILDPAARVTGMKKWPRTPKEEAQSLE 1100
DLAGFKELFQTPGPSEESMTDEKTTKIACKSPPPESVDTPTSTKQWPKRSLRKADVEEEFLALRKLTPSAGKAMLTPKPAGGDEKDIKAFMGTPVQKLDL 1200
AGTLPGSKRQLQTPKEKAQALEDLAGFKELFQTPGHTEELVAAGKTTKIPCDSPQSDPVDTPTSTKQRPKRSIRKADVEGELLACRNLMPSAGKAMHTPK 1300
PSVGEEKDIIIFVGTPVQKLDLTENLTGSKRRPQTPKEEAQALEDLTGFKELFQTPGHTEEAVAAGKTTKMPCESSPPESADTPTSTRRQPKTPLEKRDV 1400
QKELSALKKLTQTSGETTHTDKVPGGEDKSINAFRETAKQKLDPAASVTGSKRHPKTKEKAQPLEDLAGLKELFQTPVCTDKPTTHEKTTKIACRSQPDP 1500
VDTPTSSKPQSKRSLRKVDVEEEFFALRKRTPSAGKAMHTPKPAVSGEKNIYAFMGTPVQKLDLTENLTGSKRRLQTPKEKAQALEDLAGFKELFQTRGH 1600
TEESMTNDKTAKVACKSSQPDPDKNPASSKRRLKTSLGKVGVKEELLAVGKLTQTSGETTHTHTEPTGDGKSMKAFMESPKQILDSAASLTGSKRQLRTP 1700
KGKSEVPEDLAGFIELFQTPSHTKESMTNEKTTKVSYRASQPDLVDTPTSSKPQPKRSLRKADTEEEFLAFRKQTPSAGKAMHTPKPAVGEEKDINTFLG 1800
TPVQKLDQPGNLPGSNRRLQTRKEKAQALEELTGFRELFQTPCTDNPTTDEKTTKKILCKSPQSDPADTPTNTKQRPKRSLKKADVEEEFLAFRKLTPSA 1900
GKAMHTPKAAVGEEKDINTFVGTPVEKLDLLGNLPGSKRRPQTPKEKAKALEDLAGFKELFQTPGHTEESMTDDKITEVSCKSPQPDPVKTPTSSKQRLK 2000
ISLGKVGVKEEVLPVGKLTQTSGKTTQTHRETAGDGKSIKAFKESAKQMLDPANYGTMERWPRTPKEEAQSLEDLAGFKELFQTPDHTEESTTDDKTTK 2100
IACKSPPPESMDTPTSTRRRPKTPLGKRDIVEELSALKQLTQTTHTDKVPGDEDKGINVFRETAKQKLDPAASVTGSKRQPRTPKGKAQPLEDLAGLKEL 2200
FQTPICTDKPTTHEKTTKIACRSPQPDPVGTPTIFKPQSKRSLRKADVEEESLALRKRTPSVGKAMDTPKPAGGDEKDMKAFMGTPVQKLDLPGNLPGSK 2300
RWPQTPKEKAQALEDLAGFKELFQTPGTDKPTTDEKTTKIACKSPQPDPVDTPASTKQRPKRNLRKADVEEEFLALRKRTPSAGKAMDTPKPAVSDEKNI 2400
NTFVETPVQKLDLLGNLPGSKRQPQTPKEKAEALEDLVGFKELFQTPGHTEESMTDDKITEVSCKSPQPESFKTSRSSKQRLKIPLVKVDMKEEPLAVSK 2500
LTRTSGETTQTHTEPTGDSKSIKAFKESPKQILDPAASVTGSRRQLRTRKEKARALEDLVDFKELFSAPGHTEESMTIDKNTKIPCKSPPPELTDTATST 2600
KRCPKTRPRKEVKEELSAVERLTQTSGQSTHTHKEPASGDEGIKVLKQRAKKKPNPVEEEPSRRRPRAPKEKAQPLEDLAGFTELSETSGHTQESLTAGK 2700
ATKIPCESPPLEVVDTTASTKRHLRTRVQKVQVKEEPSAVKFTQTSGETTDADKEPAGEDKGIKALKESAKQTPAPAASVTGSRRRPRAPRESAQAIEDL 2800
AGFKDPAAGHTEESMTDDKTTKIPCKSSPELEDTATSSKRRPRTRAQKVEVKEELLAVGKLTQTSGETTHTDKEPVGEGKGTKAFKQPAKRKLDAEDVIG 2900
SRRQPRAPKEKAQPLEDLASFQELSQTPGHTEELANGAADSFTSAPKQTPDSGKPLKISRRVLRAPKVEPVGDVVSTRDPVKSQSKSNTSLPPLPFKRGG 3000
GKDGSVTGTKRLRCMPAPEEIVEELPASKKQRVAPRARGKSSEPVVIMKRSLRTSAKRIEPAEELNSNDMKTNKEEHKLQDSVPENKGISLRSRRQNKTE 3100
AEQQITEVFVLAERIEINRNEKKPMKTSPEMDIQNPDDGARKPIPRDKVTENKRCLRSARQNESSQPKVAEESGGQKSAKVLMQNQKGKGEAGNSDSMCL 3200
RSRKTKSQPAASTLESKSVQRVTRSVKRCAENPKKAEDNVCVKKIRTRSHRDSEDI

tr|A0A087WX41|A0A087WX41_HUMAN Clathrin heavy chain 2 OS=Homo sapiens GN=CLTCL1 PE=1 SV=1 G 443 E

10 20 30 40 50 60 70 80 90 100
MAQILPVRFQEHFQLQNLGINPANIGFSTLTMESDKFICIREKVGEQAQVTIIDMSDPMAPIRRPISAESAIMNPASKVIALKAGKTLQIFNIEMKSKMK
110 120 130 140 150 160 170 180 190 200
AHTMAEEVIFWKWVSVNTVALVTETAVYHWSMEGDSQPMKMFDRHTSLVGCQVIHYRTDEYQKWLLLVGISAQQNRVVGAMQLYSVDRKVSQPIEGHAAA
210 220 230 240 250 260 270 280 290 300
FAEFKMEGNAKPATLFCFAVRNPTGGKLHIIEVGQPAAGNQPFVKKAVDVFFPPEAQNDFPVAMQIGAKHGVIYLITKYGYLHLYDLESGVCICMNRISA
310 320 330 340 350 360 370 380 390 400
DTIFVTAPHKPTSGIIGVNKKGQVLSVCVEEDNIVNYATNVLQNPDLGLRLAVRSNLAGAEKLFVRKFNTLFAQGSYAEAAKVAASAPKGILRTRETVQK
410 420 430 440 450 460 470 480 490 500
FQSIPAQSGQASPLLQYFGILLDQGQLNKLESLELCHLVLQQEGRKQLLEKWLKEDKLECSEELGDLVKTTDPMLALSVYLRANVPSKVIQCFAETGQFQ
510 520 530 540 550 560 570 580 590 600
KIVLYAKKVGYTPDWIFLLRGVMKISPEQGLQFSRMLVQDEEPLANISQIVDIFMENSLIQQCTSFLLDALKNNRPAEGLLQTWLLEMNLVHAPQVADAI
610 620 630 640 650 660 670 680 690 700
LGNKMFTHYDRAHIAQLCEKAGLLQQALEHYTDLYDIKRAVVHTHLLNPEWLVNFFGSLSVEDSVECLHAMLSANIRQNLQLCVQVASKYHEQLGTQALV
710 720 730 740 750 760 770 780 790 800
ELFESFKSYKGLFYFLGSIVNFSQDPDVHLKYIQAACKTGQIKEVERICRESSCYNPERVKNFLKEAKLTDQLPLIIVCDRFGFVHDLVLYLYRNNLQRY
810 820 830 840 850 860 870 880 890 900
IEIYVQKVNPSRTPAVIGGLLDVDCSEEVIKHLIMAVRGQFSTDELVAEVEKRNRLKLLLPWLESQIQEGCEEPATHNALAKIYIDSNNSPECFLRENAY
910 920 930 940 950 960 970 980 990 1000
YDSSVVGRYCEKRDPHLACVAYERGQCDLELIKVCNENSLFKSEARYLVCRKDPELWAHVLEETNPSRRQLIDQVVQTALSETRDPEEISVTVKAFMTAD
1010 1020 1030 1040 1050 1060 1070 1080 1090 1100
LPNELIELLEKIVLDNSVFSEHRNLQNLLILTAIKADRTRVMEYISRLDNYDALDIASIAVSSALYEEAFTVFHKFDMNASAIQVLIEHIGNLDRAYEFA
1110 1120 1130 1140 1150 1160 1170 1180 1190 1200
ERCNEPAVWSQLAQAQLQKDLVKEAINSYIRGDDPSSYLEVVQSASRSNNWEDLVKFLQMARKKGRESYIETELIFALAKTSRVSELEDFINGPNNAHIQ
1210 1220 1230 1240 1250
QVGDRCYEEGMYEAAKLLYSNVSNFARLASTLVHLGVLCLHGWTRVPLRTAVWSSHRHSCR

tr|A0A087WY49|A0A087WY49_HUMAN Unconventional myosin−XIX OS=Homo sapiens GN=MYO19 PE=4 SV=1 K 118 R

```
         10        20        30        40        50        60        70        80        90       100
MLQQVNGHNPGSDGQAREYLREDLQEFLGGEVLLYKLDDLTRVNPVTLETVLRCLQARYMADTFYTNAGCTLVALNPFKPVPQLYSPELMREYHAAPQPQ
         110       120       130       140       150       160       170
KLKPHVFTVGEQTYRNVRKSLIEPVNQSIVVSGESGAGKTWTSRCLMKFYAVVATSPASWESHKIAERIEQRILNSNPVMEAFGLSK
```

tr|A0A087WYA7|A0A087WYA7_HUMAN Putative methyltransferas
e C9orf114 OS=Homo sapiens GN=C9orf114 PE=1 SV=1 M 210 I

```
         10        20        30        40        50        60        70        80        90       100
MAERGRKRPCGPGEHGQRIEWRKWKQQKKEEKKKWKDLKLMKKLERQRAQEEQAKRLEEEEAAAEKEDRGRPYTLSVALPGSILTMLSRRSFAPTWPVRL
        110       120       130       140       150       160       170       180       190       200
PEPVPSSVWMRSWCLMRRARMPRLWRGNSQELEEGAGVRTAGPDPAVPECPQYLRKAFFPKHQDLQFAGLLNPLDSPHHMRQDEESEFREGIVVDRPTRP
        210       220       230       240       250       260       270       280       290       300
GHGSFVNCGIMKKEVKIDKNLEPGLRVTVRLNQQQHPDCKTYHGKVVSSQDPRTKAGLYWGYTVRLASCLSAVFAEAPFQDGYDLTIGTSERGSDVASAS
        310       320       330       340       350       360       370
FPTSGMLLWCSGASRVWKLERMLTPTWRWLNPVSSLTCTSIPVLARVAVPSARRKPSSSPWPPCSLASSRRVPGTPESSKGPRTSVKQQ
```

tr|A0A087WYS3|A0A087WYS3_HUMAN Protein kinase C−binding protein 1 OS=Homo sapiens GN=ZMYND8 PE=1 SV=1 S 1005 A

```
         10        20        30        40        50        60        70        80        90       100
MDISTRSKDPGSAERTAQKRKFPSPPHSSNGHSPQDTSTSPIKKKKKPGLLNSNNKEQSELRHGPFYYMKQPLTTDPVDVVPQDGRNDFYCWVCHREGQV
        110       120       130       140       150       160       170       180       190       200
LCCELCPRVYHAKCLRLTSEPEGDWFCPECEKITVAECIETQSKAMTMLTIEQLSYLLKFAIQKMKQPGTDAFQKPVPLEQHPDYAEYIFHPMDLCTLEK
        210       220       230       240       250       260       270       280       290       300
NAKKKMYGCTEAFLADAKWILHNCIIYNGGNHKLTQIAKVVIKICEHEMNEIEVCPECYLAACQKRDNWFCEPCSNPHPLVWAKLKGFPFWPAKALRDKD
        310       320       330       340       350       360       370       380       390       400
GQVDARFFGQHDRAWVPINNCYLMSKEIPFSVKKTKSIFNSAMQEMEVYVENIRRKFGVFNYSPFRTPYTPNSQYQMLLDPTNPSAGTAKIDKQEKVKLN
        410       420       430       440       450       460       470       480       490       500
FDMTASPKILMSKPVLSGGTGRRISLSDMPRSPMSTNSSVHTGSDVEQDAEKKATSSHFSASEESMDFLDKSTASPASTKTGQAGSLSGSPKPFSPQLSA
        510       520       530       540       550       560       570       580       590       600
PITTKTDKTSTTGSILNLNLDRSKAEMDLKELSESVQQQSTPVPLISPKRQIRSRFQLNLDKTIESCKAQLGINEISEDVYTAVEHSDSEDSEKSDSSDS
        610       620       630       640       650       660       670       680       690       700
EYISDDEQKSKNEPEDTEDKEGCQMDKEPSAVKKKPKPTNPVEIKEELKSTSPASEKADPGAVKDKASPEPEKDFSEKAKPSPHPIKDKLKGKDETDSPT
        710       720       730       740       750       760       770       780       790       800
VHLGLDSDSESELVIDLGEDHSGREGRKNKKEPKEPSPKQDAVQQKEITQSPSTSTITLVTSTQSSPLVTSSGSMSTLVSSVNADLPIATASADVAADIA
        810       820       830       840       850       860       870       880       890       900
KYTSKMMDAIKGTMTEIYNDLSKNTTGSTIAEIRRLRIEIEKLQWLHQQELSEMKHNLELTMAEMRQSLEQERDRLIAEVKKQLELEKQQAVDETKKKQW
        910       920       930       940       950       960       970       980       990      1000
CANCKKEAIFYCCWNTSYCDYPCQQAHWPEHMKSCTQSATAPQQEADAEVNTETLNKSSQGSSSSTQSAPSETASASKEKETSAEKSKESGSTLDLSGSR
       1010      1020      1030      1040      1050      1060      1070
ETPSASILLGSNQGSVSKRCDKQPAYAPTTTDHQPHPNYPAQKYHSRSNKSSWSSSDEKRGSTRSDHNTSTSTKSLLPKESRLDTFWD
```

tr|A0A087WZQ9|A0A087WZQ9_HUMAN Unconventional myosin−XI X (Fragment) OS=Homo sapiens GN=MYO19 PE=4 SV=1 K 118 R

10 20 30 40 50 60 70 80 90 100
MLQQVNGHNPGSDGQAREYLREDLQEFLGGEVLLYKLDDLTRVNPVTLETVLRCLQARYMADTFYTNAGCTLVALNPFKPVPQLYSPELMREYHAAPQPQ
110 120 130 140 150
KLKPHVFTVGEQTYRNVRKSLIEPVNQSIVVSGESGAGKTWTSRCLMKFYAVVATSPASWESH

tr|B5MCN0|B5MCN0_HUMAN Atlastin−2 OS=Homo sapiens GN=ATL2 PE=1 SV=1 K 225 E

MDTQGAFDSQSTIKDCATVFALSTMTSSVQVYNLSQNIQEDDLQHLQLFTEYGRLAMEEIYQKPFQTLMFLIRDWSYPYEHSYGLEGGKQFLEKRLQVKQ

NQHEELQNVRKHIHNCFSNLGCFLLPHPGLKVATNPSFDGRLKDIDEDFKRELRNLVPLLLAPENLVEKEISGSKVTCRDLVEYFKAYIKIYQGEELPHP

KSMLQATAEANNLAAVAGARDTYCEKSMEQVCGGDKPYIAPSDLERKHLDLKEVAIKQFRSVKKMGGDEFCRRYQDQLEAEIEETYANFIKHNDGKNIFY

AARTPATLFAVMFAMYIISGLTGFIGLNSIAVLCNLVMGLALIFLCTWAYVKYSGEFREIGTVIDQIAETLWEQRSPRKVFSKLFEVTRRRMVHRALSSA

QRQRLSSNNNKKKN

tr|B7Z6C8|B7Z6C8_HUMAN Solute carrier family 25 mem
ber 46 OS=Homo sapiens GN=SLC25A46 PE=2 SV=1 V 12 L

```
         10        20        30        40        50        60        70        80        90        100
MPFYSASLIETLVQSEIIRDNTGILECVKEGIGRVIGMGVPHSKRLLPLLSLIFPTVLHGVLHYIISSVIQKFVLLILKRKTYNSHLAESTSPVQSMLDA
         110       120       130       140       150       160       170       180       190
YFPELIANFAASLCSDVILYPLETVLHRLHIQGTRTIIDNTDLGYEVLPINTQYEGMRDCINTIRQEEGVFGFYKGFGAVIIQYTLHAAVLQITKIIYST
LLQNNI
```

tr|C6GKH1|C6GKH1_HUMAN Interleukin−32 OS=Homo sapiens GN=IL32 PE=1 SV=1 D 10 N

```
         10        20        30        40        50        60        70        80        90       100
MCFPKVLSDNDMKKLKARMHQAIERFYDKMQNAESGRGQVMSSLAELEELTPLLEKERDGLRCRGNRSPVPDVEDPATEEPGESFCDKVMRWFQAMLQRL
        110       120       130       140       150
QTWWHGVLAWVKEKVVALVHAVQALWKQFQSFCCSLSELFMSSFQSYGAPRGDKEELTPQKCSEPQSSK
```

tr|C9J180|C9J180_HUMAN Ubiquitin−conjugating enzyme E2
E2 (Fragment) OS=Homo sapiens GN=UBE2E2 PE=1 SV=1 T 14 A

```
         10        20        30        40        50        60        70        80        90       100
MSTEAQRVDDSPSATSGGSSDGDQRESVQQEPEREQVQPKKKEGKISSKTAAKLSTSAKRIQKELAEITLDPPPNCSTNITLKGNAHSILNWIFGFWMPT
        110       120       130       140       150
SAGPKGDNIYEWRSTILGPPGSVYEGGVFFLDITFSPDYPFKPPKVTFRTRIYHCNINSQGVICL
```

tr|C9J8D4|C9J8D4_HUMAN Mannosyl−oligosaccharide glucosid
ase (Fragment) OS=Homo sapiens GN=MOGS PE=1 SV=4 R 400 Q

```
         10        20        30        40        50        60        70        80        90       100
MKTRSPKPLLTGLMWAQQGTTPGTPKLRHTCEQGDGVGPYGWEFHDGLSFGRQHIQDGALRLTTEFVKRPGGQHGGDWSWRVTVEPQDSGTSALPLVSLF
        110       120       130       140       150       160       170       180       190       200
FYVVTDGKEVLLPEVGAKGQLKFISGHTSELGDFRFTLLPPTSPGDTAPKYGSYNVFWTSNPGLPLLTEMVKSRLNSWFQHRPPGAPPERYLGLPGSLKW
        210       220       230       240       250       260       270       280       290       300
EDRGPSGQGQGQFLIQQVTLKIPISIEFVFESGSAQAGGNQALPRLAGSLLTQALESHAEGFRERFEKTFQLKEKGLSSGEQVLGQAALSGLLGGIGYFY
        310       320       330       340       350       360       370       380       390       400
GQGLVLPDIGVEGSEQKVDPALFPPVPLFTAVPSRSFFPRGFLWDEGFHQLVVQRWDPSLTREALGHWLGLLNADGWIGREQILGDEARARVPPEFLVQQ
        410       420       430       440       450       460       470       480
RAVHANPPTLLLPVAHMLEVGDPDDLAFLRKALPRLHAWFSWLHQSQAGPLPLSYRWRGRDPALPTLLNPKTLPSGLDDYPRASHPSVTERH
```

tr|E5RGC7|E5RGC7_HUMAN 28S ribosomal protein S28, mitochon
drial (Fragment) OS=Homo sapiens GN=MRPS28 PE=1 SV=1 L 62 F

10 20 30 40 50 60 70 80 90

MLRHSPLTQMGPAKDKLVIGRIFHIVENDLYIDFGGKFHCVCRRPEVDGEKYQKGTRVRLRFLLDLELTSRFLGATTDTTVLEANAVLLGIQESKDSRSK
EEHH

tr|E9PC05|E9PC05_HUMAN Integrin−linked kinase−associated serine/threon
ine phosphatase 2C (Fragment) OS=Homo sapiens GN=ILKAP PE=1 SV=1 A 54 T

10 20 30 40 50 60 70 80 90 100
MDLFGDLPEPERSPRPAAGKEAQKGPLLFDDLPPASSTDSGSGGPLLFDDLPPTASSGDSGSLATSISQMVKTEGKGAKRKTSEEEKNGSEELVEKKVSS
110 120 130 140 150 160 170 180 190 200
SVIFGLKGYVAERKGEREEMQDAHVILNDITEECRPPSSLITRVSYFAVFDGHGGIRASKFAAQNLHQNLIRKFPKGDVISVEKTVKRCLLDTFKHTDEE
FLKQASSQKPAWK

tr|E9PKH2|E9PKH2_HUMAN Serpin H1 OS=Homo sapiens GN=SERPINH1 PE=1 SV=1 K 163 R

```
          10        20        30        40        50        60        70        80        90       100
MVDNRGFMVTRSYTVGVMMMHRTGLYNYYDDEKEKLQIVEMPLAHKLSSLIILMPHHVEPLERLEKLLTKEQLKIWMGKMQKKAVAISLPKGVVEVTHDL
         110       120       130       140       150       160       170       180       190
QKHLAGLGLTEAIDKNKADLSRMSGKKDLYLASVFHATAFELDTDGNPFDQDIYGREELRSPRKLFYADHPFIFLVRDTQSGSLLFIGRLVRPKGDKMRD
EL
```

tr|E9PMV1|E9PMV1_HUMAN Plectin (Fragment) OS=Homo sapiens GN=PLEC PE=1 SV=1 A 396 V

```
                 10        20        30        40        50        60        70        80        90       100
MSGEDAEVRAVSEDVSNGSSGSPSPGDTLPWNLGKTQRSRRSGGGAGSNGSVLDPAERAVIRIADERDRVQKKTFTKWVNKHLIKHWRAEAQRHISDLYE
                110       120       130       140       150       160       170       180       190       200
DLRDGHNLISLLEVLSGDSLPRERDVSRSSRLPREKGRMRFHKLQNVQIALDYLRHRQVKLVNIRNDDIADGNPKLTLGLIWTIILHFQISDIQVSGQSE
                210       220       230       240       250       260       270       280       290       300
DMTAKEKLLLWSQRMVEGYQGLRCDNFTSSWRDGRLFNAIIHRHKPLLIDMNKVYRQTNLENLDQAFSVAERDLGVTRLLDPEDVDVPQPDEKSIITYVS
                310       320       330       340       350       360       370       380       390       400
SLYDAMPRVPDVQDGVRANELQLRWQEYRELVLLLLQWMRHHTAAFEERRFPSSFEEIEILWSQFLKFKEMELPAKEADKNRSKGIYQSLEGAVQVAGQL
                410       420       430       440       450       460       470       480       490       500
KVPPGYHPLDVEKEWGKLHVAILEREKQLRSEFERLECLQRIVTKLQMEAGLCEEQLNQADALLQSDVRLLAAGKVPQRAGEVERDLDKADSMIRLLFND
                510       520       530       540       550       560       570       580       590       600
VQTLKDGRHPQGEQMYRRVYRLHERLVAIRTEYNLRLKAGVAAPATQVAQVTLQSVQRRPELEDSTLRYLQDLLAWVEENQHRVDGAEWGVDLPSVEAQL
                610       620       630       640       650       660       670       680       690
GSHRGLHQSIEEFRAKIERARSDEGQLSPATRGAYRDCLGRLDLQYAKLLNSSKARLRSLESLHSFVAAATKELMWLNEKEEEEVGFDWSDRNTNMTAKK
ESYS
```

tr|E9PRV7|E9PRV7_HUMAN Beta−adrenergic receptor kinase 1 OS=Homo sapiens GN=ADRBK1 PE=1 SV=1 S 406 R

10 20 30 40 50 60 70 80 90 100
MADLEAVLADVSYLMAMEKSKATPAARASKKILLPEPSIRSVMQKYLEDRGEVTFEKIFSQKLGYLLFRDFCLNHLEEARPLVEFYEEIKKYEKLETEEE

110 120 130 140 150 160 170 180 190 200
RVARSREIFDSYIMKELLACSHPFSKSATEHVQGHLGKKQVPPDLFQPYIEEICQNLRGDVFQKFIESDKFTRFCQWKNVELNIHLTMNDFSVHRIIGRG

210 220 230 240 250 260 270 280 290 300
GFGEVYGCRKADTGKMYAMKCLDKKRIKMKQGETLALNERIMLSLVSTGDCPFIVCMSYAFHTPDKLSFILDLMNGGDLHYHLSQHGVFSEADMRFYAAE

310 320 330 340 350 360 370 380 390
IILGLEHMHNRFVVYRDLKPANILLDEHGHVRISDLGLACDFSKKKPHASVGTHGYMAPEVLQKGSRCLCGVAPSPPRGWVGAPSPPVYSFPGAFLCRFL

NVILKRSEK

tr|F2Z2W7|F2Z2W7_HUMAN tRNA (uracil−5−)−methyltransfera
se homolog A OS=Homo sapiens GN=TRMT2A PE=1 SV=1 G 399 R

```
                 10        20        30        40        50        60        70        80        90       100
MSENLDNEGPKPMESCGQESSSALSCPTVSVPPAAPAALEEVEKEGAGAATGPGPQPGLYSYIRDDLFTSEIFKLELQNVPRHASFSDVRRFLGRFGLQP
                110       120       130       140       150       160       170       180       190       200
HKTKLFGQPPCAFVTFRSAAERDKALRVLHGALWKGRPLSVRLARPKADPMARRRRQEGESEPPVTRVADVVTPLWTVPYAEQLERKQLECEQVLQKLAK
                210       220       230       240       250       260       270       280       290       300
EIGSTNRALLPWLLEQRHKHNKACCPLEGVRPSPQQTEYRNKCEFLVGVGVDGEDNTVGCRLGKYKGGTCAVAAPFDTVHIPEATKQVVKAFQEFIRSTP
                310       320       330       340       350       360       370       380       390       400
YSAYDPETYTGHWKQLTVRTSRRHQAMAIAYFHPQKLSPEELAELKTSLAQHFTAGPGRASGVTCLYFVEEGQRKTPSQEGLPLEHVAGDRCIHEDLLRG
                410       420       430       440       450       460       470       480       490       500
LTFRISPHAFFQVNTPAAEVLYTVIQDWAQLDAGSMVLDVCCGTGTIGLALARGPMYSPPWVGRHHAFLFQKVKRVIGVELCPEAVEDARVNAQDNELSN
                510       520       530       540       550       560       570       580       590       600
VEFHCGRAEDLVPTLVSRLASQHLVAILDPPRAGLHSKVILAIRRAKNLRRLLYVSCNPRAAMGNFVDLCRAPSNRVKGIPFRPVKAVAVDLFPQTPHCE
                610       620       630
MLILFERVEHPNGTGVLGPHSPPAQPTPGPPDNTLQETGTFPSS
```

tr|F5GXQ0|F5GXQ0_HUMAN BRO1 domain−containing protein BROX OS=Homo sapiens GN=BROX PE=1 SV=1 Y 249 H

10 20 30 40 50 60 70 80 90 100
MTHWFHRNPLKATAPVSFNYYGVVTGPSASKICNDLRSSRARLLELFTDLSCNPEMMKNAADSYFSLLQGFINSLDESTQESKLRYIQNFKWTDTLQGQV
110 120 130 140 150 160 170 180 190 200
PSAQQDAVFELISMGFNVALWYTKYASRLAGKENITEDEAKEVHRSLKIAAGIFKHLKESHLPKLITPAEKGRDLESRLIEAYVIQCQAEAQEVTIARAI
210 220 230 240 250 260 270 280 290 300
ELKHAPGLIAALAYETANFYQKADHTLSSLEPAYSAKWRKYLHLKMCFHYTAYAYCYHGETLLASDKCGEAIRSLQEAEKLYAKAEALCKEYGETKGPGP
310 320
TVKPSGHLFFRKLGNLVKNTLEKCQRENGFILNPNQKKK

tr|F8VVN4|F8VVN4_HUMAN Interleukin−32 OS=Homo sapiens GN=IL32 PE=4 SV=1 D 10 N

```
         10        20        30        40        50        60        70        80        90       100
MCFPKVLSDNDMKKLKARMVMLLPTSAQGLGAWVSACDTEDTVGHLGPWRDKDPALWCQLCLSSQHQAIERFYDKMQNAESGRGQDDFKEGYLETVAAYY
        110       120       130       140       150       160       170       180       190       200
EEQHPELTPLLEKERDGLRCRGNRSPVPDVEDPATEEPGESFCDKVMRWFQAMLQRLQTWWHGVLAWVKEKVVALVHAVQALWKQFQSFCCSLSELFMSS
        210
FQSYGAPRGDKEELTPQKCSEPQSSK
```

tr|G5E965|G5E965_HUMAN Forkhead box P1, isoform CRA_f OS=Homo sapiens GN=FOXP1 PE=4 SV=1 L 158 P

```
                 10        20        30        40        50        60        70        80        90       100
MMQESGTETKSNGSAIQNGSGGSNHLLECGGLREGRSNGETPAVDIGAADLAHAQQQQQQALQVARQLLLQQQQQQVSGLKSPKRNDKQPALQVPVSVA
                110       120       130       140       150       160       170       180       190       200
MMTPQVITPQQMQQILQQQVLSPQQLQVLLQQQQALMLQQQQLQEFYKKQQEQLQLQPLLQQQHAGKQPKEQQQVATQQLAFQQQLLQMQQLQQQHLLSL
                210       220       230       240       250       260       270       280       290       300
QRQGLLTIQPGQPALPLQPLAQGMIPTELQQLWKEVTSAHTAEETTGNNHSSLDLTTTCVSSSAPSKTSLIMNPHASTNGQLSVHTPKRESLSHEEHPHS
                310       320       330       340       350       360       370       380       390       400
HPLYGHGVCKWPGCEAVCEDFQSFLKHLNSEHALDDRSTAQCRVQMQVVQQLELQLAKDKERLQAMMTHLHVKSTEPKAAPQPLNLVSSVTLSKSASEAS
                410       420       430       440       450       460       470       480       490       500
PQSLPHTPTTPTAPLTPVTQGPSVITTTSMHTVGPIRRRYSDKYNVPISSADIAQNQEFYKNAEVRPPFTYASLIRQAILESPEKQLTLNEIYNWFTRMF
                510       520       530       540       550
AYFRRNAATWKNAVRHNLSLHKCFVRVENVKGAVWTVDEVEFQKRRPQKISGVPFAPTSHCISALSE
```

tr|G8JLG1|G8JLG1_HUMAN Structural maintenance of chromo
somes protein OS=Homo sapiens GN=SMC1A PE=1 SV=2 N 163 S

```
10 20 30 40 50 60 70 80 90 100
MLEVSIPTPHRYVRGKSNLMDAISFVLGEKTSNLRVKTLRDLIHGAPVGKPAANRAFVSMVYSEEGAEDRTFARVIVGGSSEYKINNKVVQLHEYSEELE
110 120 130 140 150 160 170 180 190 200
KLGILIKARNFLVFQGAVESIAMKNPKERTALFEEISRSGELAQEYDKRKKEMVKAEEDTQFSNYHRKKNIAAERKEAKQEKEEADRYQRLKDEVVRAQV
210 220 230 240 250 260 270 280 290 300
QLQLFKLYHNEVEIEKLNKELASKNKEIEKDKKRMDKVEDELKEKKKELGKMMREQQQIEKEIKEKDSELNQKRPQYIKAKENTSHKIKKLEAAKKSLQN
310 320 330 340 350 360 370 380 390 400
AQKHYKKRKGDMDELEKEMLSVEKARQEFEERMEEESQSQGRDLTLEENQVKKYHRLKEEASKRAATLAQELEKFNRDQKADQDRLDLEERKKVETEAKI
410 420 430 440 450 460 470 480 490 500
KQKLREIEENQKRIEKLEEYITTSKQSLEEQKKLEGELTEEVEMAKRRIDEINKELNQVMEQLGDARIDRQESSRQQRKAEIMESIKRLYPGSVYGRLID
510 520 530 540 550 560 570 580 590 600
LCQPTQKKYQIAVTKVLGKNMDAIIVDSEKTGRDCIQYIKEQRGEPETFLPLDYLEVKPTDEKLRELKGAKLVIDVIRYEPPHIKKALQYACGNALVCDN
610 620 630 640 650 660 670 680 690 700
VEDARRIAFGGHQRHKTVALDGTLFQKSGVISGGASDLKAKARRWDEKAVDKLKEKKERLTEELKEQMKAKRKEAELRQVQSQAHGLQMRLKYSQSDLEQ
710 720 730 740 750 760 770 780 790 800
TKTRHLALNLQEKSKLESELANFGPRINDIKRIIQSREREMKDLKEKMNQVEDEVFEEFCREIGVRNIREFEEEKVKRQNEIAKKRLEFENQKTRLGIQL
810 820 830 840 850 860 870 880 890 900
DFEKNQLKEDQDKVHMWEQTVKKDENEIEKLKKEEQRHMKIIDETMAQLQDLKNQHLAKKSEVNDKNHEMEEIRKKLGGANKEMTHLQKEVTAIETKLEQ
910 920 930 940 950 960 970 980 990 1000
KRSDRHNLLQACKMQDIKLPLSKGTMDDISQEEGSSQGEDSVSGSQRISSIYAREALIEIDYGDLCEDLKDAQAEEEIKQEMNTLQQKLNEQQSVLQRIA
1010 1020 1030 1040 1050 1060 1070 1080 1090 1100
APNMKAMEKLESVRDKFQETSDEFEAARKRAKKAKQAFEQIKKERFDRFNACFESVATNIDEIYKALSRNSSAQAFLGPENPEEPYLDGINYNCVAPGKR
1110 1120 1130 1140 1150 1160 1170 1180 1190 1200
FRPMDNLSGGEKTVAALALLFAIHSYKPAPFFVLDEIDAALDNTNIGKVANYIKEQSTCNFQAIVISLKEEFYTKAESLIGVYPEQGDCVISKVLTFDLT
KYPDANPNPNEQ
```

tr|H0Y7K8|H0Y7K8_HUMAN Structural maintenance of chromosomes p
rotein 1A (Fragment) OS=Homo sapiens GN=SMC1A PE=1 SV=2 N 163 S

```
         10        20        30        40        50        60        70        80        90       100
MLEVSIPTPHRYVRGKSNLMDAISFVLGEKTSNLRVKTLRDLIHGAPVGKPAANRAFVSMVYSEEGAEDRTFARVIVGGSSEYKINNKVVQLHEYSEELE
        110       120       130       140       150       160       170       180       190       200
KLGILIKARNFLVFQGAVESIAMKNPKERTALFEEISRSGELAQEYDKRKKEMVKAEEDTQFSNYHRKKNIAAERKEAKQEKEEADRYQRLKDEVVRAQV
        210       220       230       240       250
QLQLFKLYHNEVEIEKLNKELASKNKEIEKDKKRMDKVEDELKEKKKELGKMMREQQQIEKEI
```

tr|H0Y903|H0Y903_HUMAN Methionine aminopeptidase 1 (Fragment) OS=Homo sapiens GN=METAP1 PE=4 SV=1 R 103 P

```
         10        20        30        40        50        60        70        80        90        100
LYRNGYHGDLNETFFVGEVDDGARKLVQTTYECLMQAIDAENKAVGVMKSGHVFTIEPMICEGGWQDETWPDGWTAVTRDGKRSAQFEHTLLVTDTGCEI
LTPRRLDSARPHFMSQF
```

tr|H0YBB3|H0YBB3_HUMAN Epithelial−splicing regulatory prote
in 1 (Fragment) OS=Homo sapiens GN=ESRP1 PE=4 SV=1 VL 234 V

```
         10        20        30        40        50        60        70        80        90       100
EFKKCCPGSPDIDKLDVATMTEYLNFEKSSSVSRYGASQVEDMGNIILAMISEPYNHRFSDPERVNYKFESGTCSKMELIDDNTVVRARGLPWQSSDQDI
        110       120       130       140       150       160       170       180       190       200
ARFFKGLNIAKGGAALCLNAQGRRNGEALVRFVSEEHRDLALQRHKHHMGTRYIEVYKATGEDFLKIAGGTSNEVAQFLSKENQVIVRMRGLPFTATAEE
        210       220       230       240       250       260       270       280       290       300
VVAFFGQHCPITGGKEGILFVTYPDGRPTGDAFVLFACEEYAQNALRKHKDLLGKRYIELFRSTAAEVQQVLNRFSSAPLIPLPTPPIIPVLPQQFVPPT
        310       320       330       340       350       360       370       380       390       400
NVRDCIRLRGLPYAATIEDILDFLGEFATDIRTHGVHMVLNHQGRPSGDAFIQMKSADRAFMAAQKCHKKNMKDRYVEVFQCSAEEMNFVLMGGTLNRNG
        410       420       430       440       450       460       470       480       490       500
LSPPPCKLPCLSPPSYTFPAPAAVIPTEAAIYQPSVILNPRALQPSTAYYPAGTQLFMNYTAYYPSPPGSPNSLGYFPTAANLSGVPPQPGTVVRMQGLA
        510
YNTGVKEILNFFQGYQCLKDVW
```

tr|H0YIK5|H0YIK5_HUMAN Rac GTPase−activating protein 1 (Fragment) OS=Homo sapiens GN=RACGAP1 PE=1 SV=1 G 199 S

```
         10        20        30        40        50        60        70        80        90       100
QGNESIVAKTTVTVPNDGGPIEAVSTIETVPYWTRSRRKTGTLQPWNSDSTLNSRQLEPRTETDSVGTPQSNGGMRLHDFVSKTGMLADFVSQTSPMIPS
        110       120       130       140       150       160       170       180       190       200
IVVHCVNEIEQRGLTETGLYRISGCDRTVKELKEKFLRVKTVPLLSKVDDIHAICSLLKDFLRNLKEPLLTFRLNRAFMEAAEITDEDNSIAAMYQAVSG
        210       220       230       240       250       260       270
ELPQANRDTLAFLMIHLQRVAQSPHTKMDVANLAKVFGPTIVAHAVPNPDPVTMLQDIKRQPKVVERLLSLPLEYWSQFMM
```

tr|H7C4X9|H7C4X9_HUMAN Protein kinase C−binding protein 1 (Fragment) OS=Homo sapiens GN=ZMYND8 PE=1 SV=2 S 1005 A

```
10 20 30 40 50 60 70 80 90 100
XTDPVDVVPQDGRNDFYCWVCHREGQVLCCELCPRVYHAKCLRLTSEPEGDWFCPECEKITVAECIETQSKAMTMLTIEQLSYLLKFAIQKMKQPGTDAF
110 120 130 140 150 160 170 180 190 200
QKPVPLEQHPDYAEYIFHPMDLCTLEKNAKKKMYGCTEAFLADAKWILHNCIIYNGGNHKLTQIAKVVIKICEHEMNEIEVCPECYLAACQKRDNWFCEP
210 220 230 240 250 260 270 280 290 300
CSNPHPLVWAKLKGFPFWPAKALRDKDGQVDARFFGQHDRAWVPINNCYLMSKEIPFSVKKTKSIFNSAMQEMEVYVENIRRKFGVFNYSPFRTPYTPNS
310 320 330 340 350 360 370 380 390 400
QYQMLLDPTNPSAGTAKIDKQEKVKLNFDMTASPKILMSKPVLSGGTGRRISLSDMPRSPMSTNSSVHTGSDVEQDAEKKATSSHFSASEESMDFLDKST
410 420 430 440 450 460 470 480 490 500
ASPASTKTGQAGSLSGSPKPFSPQLSAPITTKTDKTSTTGSILNLNLDRSKAEMDLKELSESVQQQSTPVPLISPKRQIRSRFQLNLDKTIESCKAQLGI
510 520 530 540 550 560 570 580 590 600
NEISEDVYTAVEHSDSEDSEKSDSSDSEYISDDEQKSKNEPEDTEDKEGCQMDKEPSAVKKKPKPTNPVEIKEELKSTSPASEKADPGAVKDKASPEPEK
610 620 630 640 650 660 670 680 690 700
DFSEKAKPSPHPIKDKLKGKDETDSPTVHLGLDSDSESELVIDLGEDHSGREGRKNKKEPKEPSPKQDVVGKTPPSTTVGSHSPPETPVLTRSSAQTSAA
710 720 730 740 750 760 770 780 790 800
GATATTSTSSTVTVTAPAPAATGSPVKKQRPLLPKETAPAVQRVVWNSSTVQQKEITQSPSTSTITLVTSTQSSPLVTSSGSMSTLVSSVNADLPIATAS
810 820 830 840 850 860 870 880 890 900
ADVAADIAKYTSKMMDAIKGTMTEIYNDLSKNTTGSTIAEIRRLRIEIEKLQWLHQQELSEMKHNLELTMAEMRQSLEQERDRLIAEVKKQLELEKQQAV
910 920 930 940 950 960 970 980 990 1000
DETKKKQWCANCKKEAIFYCCWNTSYCDYPCQQAHWPEHMKSCTQSATAPQQEADAEVNTETLNKSSQGSSSSTQSAPSETASASKEKETSAEKSKESGS
1010 1020 1030 1040 1050 1060 1070 1080
TLDLASGSRETPSSILLGSNQGSVSKRCDKQPAYAPTTTDHQPHPNYPAQKYHSRSNKSSWSSSDEKRGSTRSDHNTSTSTKSLLPKESRLDTFWD
```

tr|M0QX73|M0QX73_HUMAN Glioma tumor suppressor candidate region gen
e 2 protein (Fragment) OS=Homo sapiens GN=GLTSCR2 PE=4 SV=1 R 246 C

```
          10        20        30        40        50        60        70        80        90       100
XLTKKRTKVQKKSLLLKKPLRVDLILENTSKVPAPKDVLAHQVPNAKKLRRKEQLWEKLAKQGELPREVRRAQARLLNPSATRAKPGPQDTVERPFYDLW
         110       120       130       140       150       160       170       180       190       200
ASDNPLDRPLVGQDEFFLEQTKKKGVKRPARLHTKPSQAPAVEVAPAGASYNPSFEDHQTLLSAAHEVELQRQKEAEKLERQLALPATEQAATQESTFQE
         210       220       230       240       250
LCEGLLEESDAELGADRLAQDPEARGQHPSRPVQELPEEEYDRASCRESQVQTQVQGEAGGEAGVP
```

tr|Q4VXL4|Q4VXL4_HUMAN HCG41426, isoform CRA_c OS=Homo sapiens GN=TACC2 PE=4 SV=1 P 145 L

```
                 10        20        30        40        50        60        70        80        90       100
MPLRRPKMKKTPEKLDNTPASPPRSPAEPNDIPIAKGTYTFDIDKWDDPNFNPFSSTSKMQESPKLPQQSYNFDPDTCDESVDPFKTSSKTPSSPSKSPA
                110       120       130       140       150       160       170       180       190       200
SFEIPASAMEANGVDGDGLNKPAKKKKTPLKTDTFRVKKSPKRSLPLSDPPSQDPTPAATPETPPVISAVVHATDEEKLAVTNQKWTCMTVDLEADKQDY
                210       220       230       240       250       260       270       280       290       300
PQPSDLSTFVNETKFSSPTEELDYRNSYEIEYMEKIGSSLPQDDDAPKKQALYLMFDTSQESPVKSSPVRMSESPTPCSGSSFEETEALVNTAAKNQHPV
                310       320       330       340       350       360       370       380       390       400
PRGLAPNQESHLQVPEKSSQKELEAMGLGTPSEAIEIREAAHPTDVSISKTALYSRIGTAEVEKPAGLLFQQPDLDSALQIARAEIITKEREVSEWKDKY
                410       420       430       440       450       460       470       480       490       500
EESRREVMEMRKIVAEYEKTIAQMIEDEQREKSVSHQTVQQLVLEKEQALADLNSVEKSLADLFRRYEKMKEVLEGFRKNEEVLKRCAQEYLSRVKKEEQ
                510       520       530       540       550       560
RYQALKVHAEEKLDRANAEIAQVRGKAQQEQAAHQASLRKEQLRVDALERTLEQKNKEIEELTKICDELIAKMGKS
```

tr|Q5QPR3|Q5QPR3_HUMAN Cyclin−dependent kinase 11A OS=Homo sapiens GN=CDK11A PE=4 SV=1 − 117 KK

```
         10        20        30        40        50        60        70        80        90        100
MGDEKDSWKVKTLDEILQEKKRRKEQEEKAEIKRLKNSDDRDSKRDSLEEGELRDHCMEITIRNSPYRREDSMEDRGEEDDSLAIKPPQQMSRKEKVHHR
         110       120       130       140       150       160       170       180       190       200
KDEKRKEKWKHARVKEKKREHERRKRHREEQDKARREWERQKRREMAREHSRRERGNDGVCLFRDRLEQLERKRERERKMREQQKEQREQKERERRAEER
         210       220       230       240       250       260       270       280       290       300
RKEREARREVSAHHRTMREDYSDKVKASHWSRSPPRPPRERFELGDGRKPVKEEKMEERDLLSDLQDISDSERKTSSAESSSAESGSGSEEEEEEEEEEE
         310       320       330       340       350       360       370       380       390       400
EEGSTSEESEEEEEEEEEEEEETGSNSEEASEQSAEEVSEEEMSEDEERENENHLLVVPESRFDRDSGESEEAEEEVGEGTPQSSALTEGDYVPDSPALL
         410       420       430       440       450       460       470       480       490       500
PIELKQELPKYLPALQGCRSVEEFQCLNRIEEGTYGVVYRAKDKKTDEIVALKRLKMEKEKEGFPITSLREINTILKAQHPNIVTVREIVVGSNMDKIYI
         510       520       530       540       550       560       570       580       590       600
VMNYVEHDLKSLMETMKQPFLPGEVKTLMIQLLRGVKHLHDNWILHRDLKTSNLLLSHAGILKVGDFGLAREYGSPLKAYTPVVVTQWYRAPELLLGAKE
         610       620       630       640       650       660       670       680       690       700
YSTAVDMWSVGCIFGELLTQKPLFPGNSEIDQINKVFKELGTPSEKIWPGYSELPVVKKMTFSEHPYNNLRKRFGALLSDQGFDLMNKFLTYFPGRRISA
         710       720       730       740       750       760       770
EDGLKHEYFRETPLPIDPSMFPTWPAKSEQQRVKRGTSPRPPEGGLGYSQLGDDDLKETGFHLTTTNQGASAAGPGFSLKF
```

tr|Q5QPR4|Q5QPR4_HUMAN Cyclin−dependent kinase 11A OS=Homo sapiens GN=CDK11A PE=4 SV=1 − 117 KK

```
         10        20        30        40        50        60        70        80        90       100
MSQSDDRDSKRDSLEEGELRDHCMEITIRNSPYRREDSMEDRGEEDDSLAIKPPQQMSRKEKVHHRKDEKRKEKCRHHSHSAEGGKHARVKEREHERRKR
        110       120       130       140       150       160       170       180       190       200
HREEQDKARREWERQKKKRREMAREHSRRERDRLEQLERKRERERKMREQQKEQREQKERERRAEERRKEREARREVSAHHRTMREDYSDKVKASHWSRS
        210       220       230       240       250       260       270       280       290       300
PPRPPRERFELGDGRKPVKEEKMEERDLLSDLQDISDSERKTSSAESSSAESGSGSEEEEEEEEEEEEEEGSTSEESEEEEEEEEEEEETGSNSEEASEQ
        310       320       330       340       350       360       370       380       390       400
SAEEVSEEEMSEDEERENENHLLVVPESRFDRDSGESEEAEEEVGEGTPQSSALTEGDYVPDSPALLPIELKQELPKYLPALQGCRSVEEFQCLNRIEEG
        410       420       430       440       450       460       470       480       490       500
TYGVVYRAKDKKTDEIVALKRLKMEKEKEGFPITSLREINTILKAQHPNIVTVREIVVGSNMDKIYIVMNYVEHDLKSLMETMKQPFLPGEVKTLMIQLL
        510       520       530       540       550       560       570       580       590       600
RGVKHLHDNWILHRDLKTSNLLLSHAGILKVGDFGLAREYGSPLKAYTPVVVTQWYRAPELLLGAKEYSTAVDMWSVGCIFGELLTQKPLFPGNSEIDQI
        610       620       630       640       650       660       670       680       690       700
NKVFKELGTPSEKIWPGYSELPVVKKMTFSEHPYNNLRKRFGALLSDQGFDLMNKFLTYFPGRRISAEDGLKHEYFRETPLPIDPSMFPTWPAKSEQQRV
        710       720       730
KRGTSPRPPEGGLGYSQLGDDDLKETGFHLTTTNQGASAAGPGFSLKF
```
